# A novel dataset on legal traditions, their determinants, and their economic role in 155 transplants

**DOI:** 10.1016/j.dib.2016.05.049

**Published:** 2016-05-31

**Authors:** Carmine Guerriero

**Affiliations:** ACLE, University of Amsterdam, Netherlands

## Abstract

The law and the economy are deeply influenced by the legal tradition or origin, which is the bundle of institutions shaping lawmaking and dispute adjudication. The two principal legal traditions, common law and civil law, have been transplanted through colonization and occupation to the vast majority of the jurisdictions in the world by a group of European countries. Here, I illustrate a novel dataset recording the lawmaking institution employed by 155 of these jurisdictions at independence and in 2000 and four discretion-curbing adjudication institutions adopted by 99 of these “transplants” at the same two points in time. Contrary to the “legal origins” scholars׳ assumption, 25 transplants changed the transplanted lawmaking institution and 95 modified at least one of the transplanted lawmaking and adjudication rules. In “Endogenous Legal Traditions” (Guerriero, 2016a) [12], I document that these reforms are consistent with a model of the design of legal institutions by societies heterogeneous in their endowment of both the extent of cultural heterogeneity and the quality of the political process. In “Endogenous Legal Traditions and Economic Outcomes” (Guerriero, 2016b) [13] moreover, I show the relevance of considering legal evolution and the endogeneity between legal traditions and economics outcomes. The data illustrated here also include the proxies for the determinants of legal evolution I use in “Endogenous Legal Traditions” (Guerriero, 2016a) [12] and the novel measure of economic outcomes I employ in “Endogenous Legal Traditions and Economic Outcomes” (Guerriero, 2016b) [13].

Specifications Table

**Table:** 

Subject area	*Economics.*
More specific subject area	*Law and Economics; Institutional Economics.*
Type of data	*Excel, PDF*
How data was acquired	*Codifying the measures of legal institutions starting from the history of each country legal order and collecting the other data from primary sources.*
Data format	*Raw, processed*
Experimental factors	*Countries with incomplete data have been discarded; legal institutions have been measured with dummies and discrete indices; the measure of legal traditions in 2000 employed in*[13] a*s well as the other reported variables are continuous.*
Experimental features	*Legal institutions are measured for the year of independence and 2000; all the other variables are measured at different points in time preceding 2000.*
Data source location	*155 countries that received their initial legal tradition externally.*
Data accessibility	*Data are with this article.*

Value of the data

• The data reveal that legal traditions are not fixed in the original transplanted form as assumed by the legal origins scholars [14], but they evolve. Hence, more theoretical and empirical research on the determinants of this evolution, as in [12], is necessary.
• The data allow to study the impact of endogenous legal traditions on the economy as in [13].
• The data are key to draw policy implications relevant for the current process of international legal harmonization.

## 1 Data

The dataset consists of cross-sectional observations on 155 countries that received their initial legal tradition exogenously mainly via colonization or occupation, i.e., transplants. For this sample, I report in the excel file “OIL_W” the lawmaking and adjudication institutions at independence and in 2000, the proxies for the determinants of their evolution I discuss in [12], and both the continuous measure of legal traditions and a measure of social welfare I employ in [13]. While the lawmaking institution determines the identity of the lawmaker – i.e., the government, the legislature, or the president under statute law and appellate judges under case law, adjudication institutions modulate the discretion allowed by the legal system to lower adjudicating courts [5], [10], [20], [22]. The drivers of the evolution of legal traditions are the extent of cultural diversity and the quality of the political process.

## 2 Experimental design, materials and methods

### 2.1 Legal traditions

The sources I use to code lawmaking institutions and identify the transplanted adjudication institutions are the first volume of the *International Encyclopedia of Comparative Law* [8] and the appendix accompanying [9], i.e., [1]. They illustrate in sufficient details the history of the legal order of the 155 transplants for which I also observe cultural diversity and/or the quality of political institutions. I exclude from this group Andorra, Guinea-Bissau, Mongolia, Mozambique, Netherlands Antilles, San Marino, and Suriname lacking sufficient information and Afghanistan, Austria, Bhutan, Denmark, France, Germany, Iraq, Maldives, Oman, Saudi Arabia, Soviet Union, Sweden, Switzerland, and the United Kingdom because origins, i.e., they developed their legal tradition internally. [3] treat also Finland, Norway, and the USA as origins but acknowledge that they could also be considered transplants [3, pp. 186–187]. I follow this second view since the post-independence legal systems of these countries were designed by Swedish, Danish, and English lawyers to resemble those of their native countries [8, p. F-39, N-76, and U-141]. The case of the Soviet Union was instead more autonomous and shaped by the desire to implement the Marxist ideal of a stable and certain legal order [8, p. U-26].

At each point in time, the lawmaking institution is either case law or statute law. To illustrate, the dummy *Case* equals one if the transplant used case law in 2000, i.e., if the decisions of a subset of appellate courts, but not just those of the Constitutional one, were considered a source of private law and treated as binding for future rulings by lower courts in 2000. Similarly, I define the dummy *Case-I* for the independence year. In the cases of multiple transplantations – e.g., Grenada, Israel, Rwanda, St. Vincent, Swaziland, Tunisia, and the ex-Eastern Bloc countries, I always focus on the most recent episode determining legal institutions at independence. To illustrate, in cases like Bulgaria and Romania, the most recent transplantation episode did not modify the legal institutions in place, which thus survived after independence [1, p. 107 and p. 660]. The dummy *Statute* turns on when *Case* equals 0 and the dummy *Statute-I* equals 1 when *Case-I* is 0. I treat Denmark, Germany, Sweden, Switzerland, and the United Kingdom as exporters of case law. This choice is consistent with a legacy of comparative law [5], [10], [20], [22]. All in all, 10 transplants switched from statute law to case law, 15 abandoned case law to embrace statute law, 69 kept statute law, and the remainder maintained case law.

For 99 of the aforementioned 155 transplants, [9] analyze the procedural rules governing the adjudication of two ubiquitous legal disputes: the collection of a bounced check and the eviction of a non-paying tenant. From this information, I obtain four variables measured in the year 2000: 1. *Comprehensive-Appeal*, which is one if issues of both law and fact can be reviewed in appeal and zero if only new evidence or issues of law can be reviewed, or if there is no appeal; 2. *Judgment-Law*, which is one if judgments must be on law only, and zero when they may be based on equity grounds; 3. *Inquisitorial*, which is 1 if judges can request or take evidence that has not been introduced by the parties and can refuse to collect or admit requested evidence, 0.5 if they have only one of the two prerogatives, and 0 otherwise; 4. *Written-Evidence*, which is one if the evidence is mostly submitted to the court in written form and zero otherwise. An important body of comparative law claims that all four variables equal one in a pure civil law jurisdiction and zero in a pure common law legal system [15, p. 52, pp. 123–127; 22, p. 272].

To measure the evolution of legal traditions, I operate as follows. First, for each adjudication institution I stack one over the other the values for the collection of a bounced check and the eviction of a non-paying tenant. Second, I build on [5], [7], [10], [20], and [22], and I obtain for the 99 transplants the four adjudication institutions at independence, i.e., *Comprehensive-Appeal-I*, *Judgment-Law-I*, *Inquisitorial-I*, and *Written-Evidence-I*. The United Kingdom and France transplanted respectively a pure common law and a pure civil law tradition, whereas Austria and the Soviet Union (Denmark, Germany, Sweden, and Switzerland) exported statute (case) law together with an intermediate level of discretion in adjudication. Finally, I construct: 1. *Common*, which is the sum of *Comprehensive-Appeal-I*, *Judgment-Law-I*, *Inquisitorial-I*, *Written-Evidence-I*, and *Statute-I* minus the sum of *Comprehensive-Appeal*, *Judgment-Law*, *Inquisitorial*, *Written-Evidence*, and *Statute* in the sample of transplants that received statute law; 2. *Civil*, which is the sum of *Comprehensive-Appeal*, *Judgment-Law*, *Inquisitorial*, *Written-Evidence*, and *Statute* minus the sum of *Comprehensive*-*Appeal*-*I*, *Judgment-Law-I*, *Inquisitorial-I*, *Written-Evidence-I*, and *Statute-I* in the sample of transplants that received case law. Thus, positive values of *Common* (*Civil*) correspond to a move toward a pure common (civil) law tradition with higher values implying deeper reforms. Estimating through a two-parameter logistic item response model the factor loadings of the five institutions characterizing a legal tradition reveals that they do not differ significantly from one whether measured at independence or in 2000, and thus they are equally important in capturing the latent “legal tradition” construct [18]. In [12], I also propose two alternative measures of the evolution of legal traditions: the dummy *Common-D* (*Civil-D*), which equals one when *Common* (*Civil*) is strictly greater than one, i.e., when relative to the bundle prevailing at independence at least one more institution typical of the pure common (civil) law tradition has been embraced. The pdf file “Measuring_LT” reports all the details of the codification exercise just explained.

In [13], I proxy the extent to which a transplant was nearer to a pure common law tradition in 2000 through the variable *Common-Law*, which is the first principal component normalized in order to range between 0 and 1 and extracted from *Case-Law* and 1 minus each of the following four aforementioned indicators for the eviction of a non-paying tenant, i.e., *Comprehensive-Appeal*, *Judgment-Law*, *Inquisitorial*, and *Written*. This choice is justified by the aforementioned evidence from two-parameter logistic item response model estimates [18].

### 2.2 Drivers of the evolution of legal traditions

In [12], I use two proxies for cultural heterogeneity. While one measures the cultural diversity in the transplant, the other gauges the cultural distance between the plurality ethnic group in the country that chose the legal tradition and the plurality ethnic group in the transplant. Cavalli-Sforza (1994) [6] suggest that the coancestry coefficient is an appropriate measure of the cultural distance between populations since it summarizes “the degree of genealogical relatedness of different populations over time. Thus, it can be interpreted as a general metrics for average differences in characteristics transmitted across generations” [19, p. 473] like preferences for punishment. When two populations split apart, their genes start to change due to random genetic drift, natural selection, and migration. The coancestry coefficient is the sum of the random drift-driven differences in the frequencies of DNA polymorphisms – i.e., situations in which a DNA sequence exists in at least two different alleles – between indigenous populations in place before 1500. Since [6] calculate the coancestry coefficient for macro-populations, I embrace the strategy proposed by Spolaore (2009) [19]. First, I identify the plurality ethnic group in each country by using ethnic composition data from [2] and information from [4]. Next, I match the plurality ethnic groups to the macro-populations reported in [6]. Finally, I normalize the resulting coancestry coefficients to make them range between 0 and 1, i.e., *Genetic-Distance*. Larger values of *Genetic-Distance* reveal a longer separation and, in turn, a larger cultural distance between populations. Cavalli-Sforza (1994) [6] also highlight the strong link between genetic pools and ethnolinguistic roots. This relationship, which is driven by the diverse cultural transmission instruments of populations that are increasingly distant from the common ancestors, makes the ethnolinguistic fractionalization in a country a natural proxy for the “within” cultural diversity. The measure of ethnolinguistic fractionalization *ELF* can be interpreted as the probability that two individuals randomly drawn from the population belong to different ethnolinguistic groups. *ELF* is based on data from the Narodov Mira atlas and [11]. Lower ethnolinguistic cohesion corresponds to higher values of *ELF*, and therefore a value of 0 (1) identifies a completely homogeneous (heterogeneous) country. In [12], I also experiment with the natural logarithm of the number of languages [16] and the ethnic fractionalization [2].

Following [17], in [12] I proxy the quality of the political process with the normalized – to range between 0 and 1 – first principal component extracted from the constraints on the executive authority score and *Polity* score collected from the POLITY IV dataset at http://www.systemicpeace.org/polity/ and averaged between independence and 2000, *Pc-Institutions*.

### 2.3 A measure of social welfare

In [13], I construct a proxy capturing simultaneously the long-run technological efficiency of the law and society׳s cultural satisfaction with legal rules building on data from the World Business Environment Survey [21]. This firm-level survey asked in 2000 managers to rate on a 6-point scale how much they believe the legal system will uphold contracts and property rights in a business dispute and how much they believe it is “fair and impartial.” While the former aspect relates to the ability of the legal system to hit its efficiency targets – i.e., expanding trade and sheltering private investment from expropriation, the latter substantiates society׳s cultural satisfaction with the law. To capture both aspects, I consider the normalized first principal component extracted from the country average of each question, i.e., *Social-Welfare*.

## References

[1] Acartürk Burcu, et al., Courts: The Lex Mundi Project. Civil Procedures Around the World, 2005, Unpublished.

[2] Alesina Alberto, Devleeschauwer Arnaud, Easterly William, Kurlat Sergio, Wacziarg Romain (2003). Fractionalization. J. Econ. Growth.

[3] Berkowitz Daniel, Pistor Katharina, Richard Jean-Francois (2003). Economic development, legality, and the transplant effect. Eur. Econ. Rev..

[4] Britannica (Encyclopedia), Encyclopedia Britannica, 〈http://www.britannica.com〉,2015.

[5] Campbell Christian, Dennis Campbell, International Civil Procedures, Lloyd׳s of London Press Ltd., London, 1995.

[6] Cavalli-Sforza Luca L., Menozzi Paolo, Piazza Alberto (1994). The History and Geography of Human Genes.

[7] Damaška Mirjan R. (1986). The Faces of Justice and State Authority.

[8] David René et al., (Ed.), International Encyclopedia of Comparative Law, Volume 1, National Reports, Oceana Publications Incorporation, New York, 1995.

[9] Djankov Simeon, La Porta Rafael, Lopez-de-Silanes Florencio, Shleifer Andrei (2003). Courts. Q. J. Econ..

[10] Engelmann Arthur (1927). A History of Continental Civil Procedure.

[11] Fearon James, Laitin David (2003). Ethnicity, insurgency and civil war. Am. Polit. Sci. Rev..

[12] Guerriero Carmine (2016). Endogenous legal traditions. Int. Rev. Law Econ..

[13] Guerriero Carmine (2016). Endogenous legal traditions and economic outcomes. J. Comp. Econ..

[14] La Porta Rafael, Lopez-de-Silanes Florencio, Shleifer Andrei (2008). The economic consequences of legal origins. J. Econ. Lit..

[15] Merryman John H. (1969). The Civil Law Tradition.

[16] Michalopoulos Stelios (2012). The origins of ethnolinguistic diversity: theory and evidence. Am. Econ. Rev..

[17] Persson Torsten, Tabellini Guido (2009). Democratic capital: the nexus of political and economic change. Am. Econ. J.: Macroecon..

[18] Rosenthal Howard, Voeten Erik (2007). Measuring legal systems. J. Comp. Econ..

[19] Spolaore Enrico, Wacziarg Romain (2009). The diffusion of development. Q. J. Econ..

[20] Ward, Richard, Amanda Wragg, English Legal System, 9th ed., Oxford University Press, Oxford-New York, 2015.

[21] World Bank (2000). World Business Environment Survey.

[22] Zweigert, Konrad, Hein Kötz, Introduction to Comparative Law, 3rd ed., Oxford University Press, Oxford-New York, 1998.

