# Supplementary material for: A novel dataset on legal traditions, their determinants, and their economic role in 155 transplants
Source: Data Brief. 2016 May 31;8:394–8. doi: 10.1016/j.dib.2016.05.049 (PMC4908268; doi:10.1016/j.dib.2016.05.049)
Supplement: Supplementary file 1 — Supplementary material [file mmc1.pdf]

Measuring Legal Traditions: Appendix Accompanying "Endogenous Legal Traditions."

Carmine Guerriero

ACLE, University of Amsterdam

April 27, 2016

Contents

1 Origins **10**

1.1 Origins that Selected Statute Law 10

1.1.1 Austria 10

1.1.2 France 11

1.1.3 USSR 13

1.2 Origins that Selected Case Law 15

1.2.1 Denmark 15

1.2.2 Germany 16

1.2.3 Sweden 18

1.2.4 Switzerland 19

1.2.5 United Kingdom 21

2 Transplants **27**

2.1 Transplants to which Statute Law was Transplanted 27

2.1.1 Albania 27

2.1.2 Algeria 27

2.1.3 Argentina 27

2.1.4 Belgium 28

2.1.5 Benin 29

2.1.6 Bolivia 29

2.1.7 Brazil 29

2.1.8 Bulgaria 30

2.1.9 Burkina Faso 30
2.1.10 Cameroon 31
2.1.11 Central African Republic 31
2.1.12 Chad 31
2.1.13 Chile 32
2.1.14 Colombia 32
2.1.15 Comoro 33
2.1.16 Congo, Republic of the 33
2.1.17 Costa Rica 33
2.1.18 Cote D'Ivoire 34
2.1.19 Croatia 34
2.1.20 Cuba 35
2.1.21 Czech Republic 35
2.1.22 Dominican Republic 36
2.1.23 Ecuador 36
2.1.24 Egypt 37
2.1.25 El Salvador 38
2.1.26 Equatorial Guinea 38
2.1.27 Estonia 39
2.1.28 Gabon 39
2.1.29 Georgia 39
2.1.30 Guatemala 40
2.1.31 Guinea 41

2.1.32 Haiti 41

2.1.33 Honduras 41

2.1.34 Hungary 42

2.1.35 Indonesia 42

2.1.36 Iran 43

2.1.37 Italy 44

2.1.38 Kazakhstan 44

2.1.39 Khmer Republic 45

2.1.40 Laos 45

2.1.41 Latvia 45

2.1.42 Libya 45

2.1.43 Liechtenstein 46

2.1.44 Lithuania 46

2.1.45 Luxembourg 46

2.1.46 Madagascar 47

2.1.47 Mali 47

2.1.48 Malta 47

2.1.49 Mauritania 48

2.1.50 Mauritius 48

2.1.51 Mexico 49

2.1.52 Monaco 49

2.1.53 Morocco 50

2.1.54 Netherlands 50

2.1.55 Nicaragua 51

2.1.56 Niger 51

2.1.57 Panama 52

2.1.58 Paraguay 52

2.1.59 Peru 53

2.1.60 Philippines 53

2.1.61 Poland 54

2.1.62 Portugal 55

2.1.63 Rwanda 55

2.1.64 Senegal 56

2.1.65 Seychelles 56

2.1.66 Slovenia 57

2.1.67 Somalia 57

2.1.68 Spain 57

2.1.69 St. Lucia 58

2.1.70 Swaziland 58

2.1.71 Syria 59

2.1.72 Togo 59

2.1.73 Tunisia 59

2.1.74 Ukraine 60

2.1.75 Uruguay 60

2.1.76 Vatican 61

2.1.77 Venezuela 61

2.1.78 Vietnam 62

2.1.79 Zaire 63

2.2 Transplants to which Case Law was Transplanted 64

2.2.1 Anguilla 64

2.2.2 Antigua 64

2.2.3 Australia 64

2.2.4 Bahrain 65

2.2.5 Bangladesh 65

2.2.6 Barbados 66

2.2.7 Belize 66

2.2.8 Bermuda 67

2.2.9 Botswana 67

2.2.10 British Virgin Island 68

2.2.11 Brunei 68

2.2.12 Burundi 69

2.2.13 Canada 69

2.2.14 Cayman 69

2.2.15 China 70

2.2.16 Cyprus 70

2.2.17 Dominica 71

2.2.18 Ethiopia 71

2.2.19 Falkland Islands 71

2.2.20 Fiji 71

2.2.21 Finland 71
2.2.22 Gambia 72
2.2.23 Ghana 72
2.2.24 Gibraltar 73
2.2.25 Greece 73
2.2.26 Grenada 74
2.2.27 Guyana 74
2.2.28 Hong Kong 75
2.2.29 Iceland 75
2.2.30 India 76
2.2.31 Ireland 76
2.2.32 Israel 77
2.2.33 Jamaica 78
2.2.34 Japan 78
2.2.35 Jordan 79
2.2.36 Kenya 80
2.2.37 Korea Republic 80
2.2.38 Kuwait 81
2.2.39 Lebanon 82
2.2.40 Lesotho 82
2.2.41 Liberia 83
2.2.42 Malawi 83
2.2.43 Malaysia 83

2.2.44 Montserrat . . . 84
2.2.45 Myanmar . . . 84
2.2.46 Namibia . . . 84
2.2.47 Nauru . . . 85
2.2.48 Nepal . . . 85
2.2.49 New Zealand . . . 86
2.2.50 Nigeria . . . 86
2.2.51 Norway . . . 87
2.2.52 Pakistan . . . 87
2.2.53 Papua New Guinea . . . 88
2.2.54 Quatar . . . 88
2.2.55 Romania . . . 89
2.2.56 Sierra Leone . . . 89
2.2.57 Singapore . . . 90
2.2.58 Solomon Island . . . 90
2.2.59 South Africa . . . 90
2.2.60 Sri Lanka . . . 91
2.2.61 St. Kitts and Nevis . . . 92
2.2.62 St. Vincent . . . 92
2.2.63 Sudan . . . 93
2.2.64 Taiwan . . . 93
2.2.65 Tanzania . . . 94
2.2.66 Thailand . . . 95

2.2.67 Tonga 95
2.2.68 Trinidad and Tobago 95
2.2.69 Turkey 96
2.2.70 Turks and Caicos 97
2.2.71 Uganda 97
2.2.72 United Arab Emirates 98
2.2.73 United States of America 99
2.2.74 Western Samoa 99
2.2.75 Zambia 100
2.2.76 Zimbabwe 100

3 References **102**

1 Origins

1.1 Origins that Selected Statute Law

1.1.1 Austria

Historical Background

"The third of the great codes which came into force in Europe at the end of the eighteenth and the beginning of the nineteenth century is the Austrian General Civil Code [ABGB] of 1811" [Zweigert and Kötz 1998, p. 158].

Lawmaking Institutions

Statute-I $= 1$: "Court decisions and writing are not considered to be source of law" [David et al. 1995, p. A-72]. Yet, they can be used to interpret the intention and to consider the analogy of statutes (ABGB §6 and §7).

Adjudication Institutions

Provisions on civil procedure were introduced by the Code of Civil procedure together with its Introductory Statute (1895), the Code on Enforcement of Judgments together with its Introductory Statute (1896), and the Statute on the Organization of the Courts (1896).

Comprehensive-Appeal-I $= 0$: "Appeal is the means provided for reviewing judgments of first instance. Here the Roman "appellation," with its unrestricted right of the party to advance new allegation and proof in the upper court has found no acceptance. Allegation and proof not appearing in the record of the court below may be received, but only to "explain or refute the points of appeal" [Engelmann 1928, p. 641].

Judgment-Law-I $= 1$: "§876 [...] provides that rules on error, deceit, and duress are to be applied "to other expressions of will requiring communications" [Zweigert and Kötz 1998, p. 164]. Yet, judges do not rule on this general equity principle (Zweigert and Kötz, 1998).

Inquisitorial-I $= 1$: "The Code has sought to widen the powers of the court with reference to the ascertainment of the truth. Accordingly [...], it is made the duty of the court, at the hearing, "by questioning or otherwise," so to proceed that all relevant allegations be brought forward and all relevant facts disclosed. And, except in the case of united objection from the parties, it may, of its motion, call for the production of documents referred by one or

the other and remaining in their possession or in that of a public official or notary, and at all times summon witnesses not named by the parties" [Engelmann 1928, p. 636].

Written-Evidence-I = 0: "Parties shall be heard orally, but [when] representation by counsel is required, there shall be preparatory written pleadings. The reading of written statements in lieu of oral declaration at the hearing is not permitted. And what is termed "the indispensable principle of an oral procedure" is expressed in the permitting of new allegation and proof down to the close of the hearing" [Engelmann 1928, p. 641].

1.1.2 France

Historical Background

"One of the most important aims of the [1789] Revolution from the very beginning was to unify the private law. The Assemblée Constituante itself had decreed: "A code of civil law common to the whole kingdom will be drawn up." [...] [T]he protracted consideration of the [Cambacérès'] draft was rudely interrupted by Napoleon's taking power at the end of 1799" [Zweigert and Kötz 1998, p. 82]. Purged the legislative bodies of all his opponents, Napoleon supervised over the work of the two experts of the droit coutumier—the common law of the northern part of France—and the two experts of the droit écrit—the adapted Roman Law tradition. "In 1803 and 1804 thirty-six separate statutes were passed without opposition; they were finally consolidated by the law of 31 March 1804 and brought into force under the title "Code civil des Française" [Zweigert and Kötz 1998, p. 83].

Lawmaking Institutions

Statute-I = 1: "In the years immediately following the enactment of the Code, legal writing went through an unfruitful and positivistic stage, merely, expounding the text of the Code, grammatically and logically, and ignoring judicial decision entirely (école de l'exégèse). The picture changed, however towards the end of the nineteenth century. [...] Gény's new school, called the "école de la libre recherche scientifique," allowed the judge a greater freedom with the text of the code and encouraged him to construe the Code not simply logically and systematically, but in the requirements of society as it developed" [Zweigert and Kötz 1998, p. 96]. Judicial interpretation however needs to be "as close as possible to enacted law and, in the nineteenth century, it was admitted that only exegetic methods, grammatical, logical

and psychological interpretation, must be used" [David et al. 1995, p. F-61]. The "principle is clear [...] that each court related only to the case in question (CC art. 5 expressly forbids the judge to make a general and regulatory decision)" [David et al. 1995, p. F-61].

Adjudication Institutions

The Code of Civil Procedure of 1806 is an improved version of the Ordinance of 1667 and has been reformed only in 1975 with the adoption of the New Code of Civil Procedure. Since all French colonies in the sample gained their independence between 1844 and 1975, the transplanted bright line rules are those contained in the Code of Civil Procedure of 1806.

Comprehensive-Appeal-I = 1: "The principle adopted by the Constituent Assembly and since maintained is that, for any cause, there shall not be more than two degrees of jurisdiction. [...]. The appeal is always devolutive, that is to say, it takes the cause or the part of it in question out of the hands of the lower court into those of the upper, which substitute its own judgment for the one attacked. [...] [Its] course is [...] subject to the general rule—which, nevertheless, under the holding of the courts, may be waived by the parties—that non "new demands" are to be introduced, new facts may be alleged and proved to the same extent as in the lower court" [Engelmann at al. 1928, p. 775 and 776]. In lower courts both matters of fact and law were always analyzed (see Engelmann at al., [1928]).

Judgment-Law-I = 1: "General principles of law [express] the superior requirements of a social and moral order which is supposed to be the inspiration of the legal order. The role of these principles is especially important on occasions where the legislative structure presents serious gaps. Administrative law provides a good example of this [...]. In private law, on the contrary, these principles are used less to supplement the law than to correct it as judges appeal to them to prevent the application in the case before them of a particular rule which they consider inopportune. This reformative function of general principles as it might be called appears more clearly on examining their content [...]. Some are sufficiently precise to be regarded directly as rules of law, e.g., the principles of unjust enrichment or monetary nominalism [...]. Other principles remain more flexible leaving a wider power of interpretation to the judge in each particular case, e.g., the principle of the relatively of rights. In the extreme case they seem to be reduced to a single principle according to which all disputes must be resolved in the light of the, sometimes contradictory, requirement of

society [...] and of equity. But this means that the judge is faced with a simple directive which bears directly on his task of interpreting the legal rule. At this point the new problem arises deciding whether or not the judge himself in exercising his interpretative function is to be considered, if not as a primary source, at least as a secondary or derivative source of law" [David et al. 1995, p. F-60 and F-61], which as seen above is not the case.

Inquisitorial-I = 1: Questions about the validity of documents relied upon by the parties is under "medial judgment of the court itself [...]. [The rejection to collect or admit evidence requested by the parties] may, however take the form of an independent action" [Engelmann 1928, p. 759] of the judge himself. "The court, by medial judgment, [...] designate the facts to be proved, orders the "enquête" [request of evidence] and names a judge-delegate to conduct it. [...] Objection to the competency of witnesses are made before the judge-delegate and noted by him [and] [...] they are decide by medial judgment. [...] Where the opponent desires to call witnesses in proof of affirmative facts, then he is required to obtain a judgment ordering a separate "enquête" [Engelmann 1928, p. 760]. Finally, order of appearance of new witnesses may be ordered by the court ex-officio [Engelmann 1928, p. 760 and 761], and probatory oaths and expert testimony can be requested by the judge lacking agreement of the party or refused when requested [Engelmann 1928, p. 761 and 762].

Written-Evidence-I = 1: "The general theory of proof is laid down [...] in the Civil Code [...]. [T]he normal form of proof is documentary evidence, that is to say, public documents ("actes authentiques") and private documents ("actes sous seing privè"). Following the principle [...] incorporated in the Ordinance of 1667, the Civil code prohibits testimonial evidence in any case involving more than one hundred fifty francs, where it would have been possible for the parties to bind themselves in writing" [Engelmann 1928, p. 758]. Other recognized forms of proof were probatory oaths, judicial inspections, and expert reports, all of which require a heavy amount of written work [see Engelmann 1928, p. 758 to 764].

1.1.3 USSR

Historical Background

"The history of Russian law can be traced back to the 10th century when Christianity was spread on Kievan Rus (present Ukraine) and the canon of the Orthodox Church became a

major legal source. During the 10th-13th centuries, Roman law was also introduced indirectly though the Bulgarian codes. Peter the Great initiated the first steps towards a legal reform between 1700 and 1720 when European influences were widely spread in Russia. The 19th century marked the beginning of the Russian legal system, influenced mostly by the German revival of Roman law. In 1835, a "Civil law" came into force as Volume 10 of the organized digest ("Svod zakonov"), which became the first Russian civil code [...]. After the 1917 revolution, Russia isolated itself from the Western cultures and influences. This resulted in an entirely new body of drafted legislation based on Marxist political doctrine. The new codifications were the Civil Code of 1922 and the Civil Procedure Code of 1923" [Acartürk et al. 2005, p. 670]. While the former was in force in the ex-soviet Union territories in the aftermath of the collapse of Eastern Bloc, the latter was reformed only by the 1961 Fundamentals of Civil Procedure and the 1964 Codes of civil procedures (David et al., 1995).

Lawmaking Institutions

Statute-I = 1: "Court decisions are not considered as a source of Soviet law. The rule is stringently applied and is dictated by the demands of the strictest observance of socialist legality, [...] stability and the precision of the legal system" [David et al. 1995, p. U-26].

Adjudication Institutions

Comprehensive-Appeal-I = 1; *Judgment-Law-I* = 1; *Inquisitorial-I* = 0.5; and *Written-Evidence-I* = 0: The Codes of Civil Procedure of 1964 states that "at an oral hearing [judges] hear directly all declarations and claims of persons who have an interest in the case [...]. During the proceedings the parties have freedom in the choice and presentation to the court of evidence at their disposal [...]. [Yet,] there must be stressed the active role of the Soviet court in decision of a dispute between parties; this is a most important principle of civil procedure. [...] Civil Procedure art. 16 lays down the rule that the court is obliged not only to limit itself to the evidence and explanations submitted but to take all measures prescribed by law for a comprehensive, full and objective elucidation of the actual circumstances of the case and the rights and the duties of the parties (the principle of objective truth). [...] Civil procedure goes regularly through two stages—the procedure in the court of first instance, which examines the essence of the case, and in the court of second instance, which on appeals from the parties (or protests by the procurator) examines the decision of the lower court [...].

[T]he higher court examines the case and validity of the appeal or protest, and if it finds it unjustified and the decision lawful, then it upholds the decision. Alternatively the higher court quashes the decision and it returns the case to the court of first instance for retrial or having quashed the decision dismisses the case or it amends the decision, and in individual cases it makes a new decision without passing the case for a new hearing, provided there is no necessity to collect and verify new evidence" [David et al. 1995, p. U-43 and U-44]. This analysis applies to all the countries of the ex-Eastern Bloc except Bulgaria (see below).

1.2 Origins that Selected Case Law

1.2.1 Denmark

Historical Background

"Prior to the 17th century, the first Danish codifications were developed on the basis of unwritten customary laws with inspiration from the common law. However, the fundamental principles of the Danish law were established in the Middle Age. In 1683, King Christian V disseminated the "Danish Law," a general code of law, which virtually incorporated the preexisting legislation. The code became the most significant source of law for the next 200 years. In 1849, Denmark adopted its first constitution abrogating the absolute monarchy and constituting a constitutional democracy. Since then, Danish law instead of modifying the code itself has been entirely altered through a series of separate laws. To a large extent, the 19th-century law was affected by German jurisprudence and the other Scandinavian legal systems. On the other hand, the modern Danish legal system is less influenced by any European systems and embraces the concept of the Scandinavian legal tradition. Its specific feature is the absence of civil and commercial law codifications [...]. The Code of Civil Procedure was enacted in 1916 and [...] amended in 1999" [Acartürk et al. 2005, p. 209].

Lawmaking Institutions

Statute-I = 0: "The principal source of law is legislation; yet, there exist no general codification of Danish law. [Where] there is no or very little legislation [...] judge-made law is the source of law. [...] Danish courts do not recognize the absolute authority of precedents. A judge, however, will not lightly disregard a prior decision, and though it is not usual to indicate in the judgment whether it has been made the reliance upon older

decisions, precedents are discussed in court" [David et al. 1995, p. D-25].

Adjudication Institutions

Procedural rules are all found in the Administration of Justice act of 1916.

Comprehensive-Appeal-I = 1: "The Court of Appeal is freely entitled to decide the matter both with respect to facts and law. The only boundaries are those imposed by the parties when preparing the case" [Campbell and Campbell 1995, p. 110].

Judgment-Law-I = 0: Under §36 of the "Law of Contracts and other legal transactions in the law of property and obligations," which came into force in Denmark, Norway, and Sweden between 1915 and 1918 and in Finland in 1929 "a court may invalidate or modify a term of a contract if, given the subject-matter of the transaction and the attendant or supervising circumstances, it can be held to be "unfair" [and] this applies in principle to commercial and non commercial transaction alike" [Zweigert and Kötz 1998, p. 282].

Inquisitorial-I = 0.5: "The court might [...] reject new claims, allegations or evidence, even if the other party has not made objections" [Campbell and Campbell 1995, p. 110].

Written-Evidence-I = 0: Because of the principal of direct evaluation, "any important explanations and statements from parties, witnesses and experts witnesses should be made or repeated orally during the hearing in court" [Campbell and Campbell 1995, p. 110].

1.2.2 Germany

Historical Background

"Under the Imperial Constitution central legislative competence in private law was limited to the law of obligations, commerce, and negotiable instruments, but in 1873 [...] this was extended to the whole private law; this was the signal for work to start on the codification of German private law [...]. In 1874 a First Commission was appointed and [...] in 1887 [it] published a First Draft with supporting "Motives." A storm of criticism broke out. [...] The Second Draft with its "Protokolle" was published in 1895, [...] the date of entry into force was set for 1 Jan. 1900" [Zweigert and Kötz 1998, p. 141 and 142].

Lawmaking Institutions

Statute-I = 0: Given that the 1895 German Civil Code—BGB, thereafter—is "impregnated with the legal values of bourgeois liberalism and is thus, to use an expression of

Radbruch, "more credence of the nineteenth century than the upbeat of the twentieth," how has it been able, without fundamental revision, to survive the political, economic, and social crises to catastrophes of recent German history, including the period of total perversion of law under Hitler? [...] Areas of law which were developing very rapidly [...] have been greatly altered and modernized by intervening legislation. Still, the maintenance of the general structure of the BGB is really the work of courts. [...] the courts in Germany have relied above all on the general clauses of §138, §157, §242, and §826 BGB" [Zweigert and Kötz 1998, p. 152 and 153]. The "so-called general clauses (e.g., the provision imposing requirements of good faith and adherence to public policy in private transactions) have provided an important stimulus and starting point for these contributions" [David et al. 1995, p. F-4] greatly differentiating in flexibility the German from the French Civil Code.

Adjudication Institutions

"Ordinary jurisdiction extends to all civil and criminal cases. [...] Court organization [...] are governed by the Constitution of Courts Act of 27 January 1877" [David et al. 1995, p. F-3]. The other fundamental source of civil procedure is the 1879 Code of Civil Procedure as amended by respectively the 1959 Federal Attorney's Code, the 1961 German Judiciary Act, and the 1969 Court Administrator Act (David et al., 1995).

Comprehensive-Appeal-I = 1: The appeal is not only "a review of the judgment rendered in the first instance: it involves a hearing and a decision de novo. [...] [T]he parties, as in the Roman law, are entitled to the "beneficium novorum," [i.e.,] the right to introduce allegation and proof not presented in the lower court" [Engelmann 1928, p. 610].

Judgment-Law-I = 0: The BGB "tendency to emphasize the mutuality of social responsibility has lead to extremely significant legal developments even in the area of private law. This "moralization" of contractual relations was made possible by the famous "general clause" BGB of §242 [but, see also §157], which is still much relied on today. That clause [says] that everyone must perform his contract in the manner required by good faith (Treu und Glauben) in view of the general practice in commerce, yet the courts, left more or less in the lurch by the draftsmen, have had to rely on it in order to solve the extremely important economic and social problem which arose after the First World War with the collapse of the economy, inflation, and revaluation, and after the Second World War from the loss of East

Germany and the change of currency [...]. The courts long used §242 BGB in order to control the content of General Conditions of Business: standard term excluding or liming one party's liability were invalidated as inconsistent with good faith if "they are inequitable on a balancing of the interests of those who typically enter such transactions" (BGH NJW 1963, 99, 100 [...]). [T]he general clause of §242 BGB has proved a splendid device for adapting the law of contract to the changed social and moral attitudes of the society. Hedemann has called this "the flight into the general clauses" [Zweigert and Kötz 1998, p. 150].

Inquisitorial-I = 1: "The common [German] law principle of party-presentation ("Verhandlungsmaxime") has been taken over. It manifest itself in the rule that the court may use as the basis for its application of law only such facts as the parties have introduced into the cause. [Proof] is rendered unnecessary by (a) judicial admission, (b) failure to deny the particular allegation, (c) judicial notice" [Engelmann 1928, p. 606 and 607].

Written-Evidence-I = 1: Parties "shall be heard orally, but that in the causes where [representation] is required, there shall be preparatory written pleadings. [...] And what is termed "the indispensable principle of an oral procedure" is expressed in the permitting of new allegation and proof down to the close of the hearing" [Engelmann 1928, p. 606].

1.2.3 Sweden

Historical Background

"The Code of 1734 can be regarded as the basis of, or perhaps rather the starting point for the later development of Swedish and Finnish law" [David et al. 1995, p. S-163].

Lawmaking Institutions

Statute-I = 0: Although written law "is regarded as the principal source of law [...] judge-made law can be said to cover certain fields. [...] Precedents play on the whole an important role for the interpretation of statutes and for the continued development of law on the basis of the guiding ideas already expressed in legislation" [David et al. 1995, p. S-163].

Adjudication Institutions

Before the Law of Judicial Procedure was ratified in 1942, procedural rules were based on both the Code of 1734 or on customary rules [see Engelmann 1928, p. 864 to 871].

Comprehensive-Appeal-I = 0: Appellate courts "review the case solely on the basis of

the written record of the proceeding below, together with the written memorials ("inlagor") lodged by the parties" [Engelmann 1928, p. 870]. A further step of appeal against appellate courts is called revision and deals not only with questions of law but also with questions of fact. Revision is, however, admitted only under special circumstances following a special procedure of admission [see Engelmann 1928, p. 864 to 871].

Judgment-Law-I = 1: Under §36 of the "Law of Contracts and other legal transactions in the law of property and obligations," which came into force in Denmark, Norway, and Sweden between 1915 and 1918 and in Finland in 1929 "a court may invalidate or modify a term of a contract if, given the subject-matter of the transaction and the attendant or supervising circumstances, it can be held to be "unfair" [and] this applies in principle to commercial and non commercial transaction alike" [Zweigert and Kötz 1998, p. 282].

Inquisitorial-I = 1: "The judge ought carefully to examine into the competency of witness [and abide by] canons respecting proof-evaluation, legal presumption, and the like, which [continue] to find acceptance" [Engelmann 1928, p. 855; but see also p. 864 to 871].

Written-Evidence-I = 0: "An attempt was made, in 1540, to establish a purely written procedure [...], but there is no indication that the relative provisions were ever closely observed. In the ordinary lower courts, especially in the country districts, the spoken word continued to be the principal medium of communication between the parties and the court. Writing come into application only as a subsidiary means" [Engelmann 1928, p. 844].

1.2.4 Switzerland

Historical Background

"Prior to unification by the federal codes (Civil Code and Code of Obligations), each canton had its own system of private law [...]. There was the influence of the French Civil Code in French-speaking Switzerland and in Ticino, and of the Austrian code in the German-speaking cantons [...]. Unifications became possible with the enactment of the Federal Constitution of 1874. It began with the adoption in 1881 of the Federal Code of Obligations [...] [and of] the Swiss Civil Code" [David et al. 1995, p. S-185].

Lawmaking Institutions

Statute-I = 0: Codification was made in general terms, leaving large discretion to judge-

made law whose "important tradition [is] best explained by the famous [Code Civil or ZGB] art. 1 which merits to be cited in full (transl. I. Williams): "The Law must be applied in all cases which come within the letter or the spirit of any of its provisions. Where no provisions is applicable, the judge shall decide according to the existing Customary Law and, in default thereof, according to the rules which he would lay down if he had himself to act as legislator. Herein he must be guided by approved legal doctrine and case law." The consequence is that judicial decisions play an important role even in areas which have been codified at the end of the last or the beginning of this century" [David et al. 1995, p. S-184 and S-185].

Adjudication Institutions

The art. 64 par. 3 of the Constitution entails that "there are 26 cantonal codes of civil procedure" [David et al. 1995, p. S-184 and S-185]. Yet, the general provisions are:

Comprehensive-Appeal-I = 1: "All cantons do grant at least one ordinary appellate remedy against judgments rendered by a lower cantonal court: the regular appeal (Berufung). This [. . .] allows an unlimited review of a judgment or jurisdictional decision by a cantonal appellate court. Consequently, an appellate court may consider all questions of fact and/or law on the taking of a regular appeal" [Campbell and Campbell 1995, p. 697].

Judgment-Law-I = 1: "Art. 2 of the ZGB reads: "Everyone . . . in exercising his rights and in performing his duties [must] act in good faith" [. . .]. [The BGB] has similar provisions which perform the same function although their more modest location in the Code [ZGB], makes their moral significance [and impact] less striking" [Zweigert and Kötz 1998, p. 174].

Inquisitorial-I = 1: "It is the judge and not the parties who are in control of the presentation of evidence" [Campbell and Campbell 1995, p. 673]. Judges can request or take evidence that has not been offered or introduced by the parties, and can refuse to collect or admit evidence requested by the parties [see Campbell and Campbell 1995, p. 673 to 677].

Written-Evidence-I = 1: "Swiss procedural law recognizes the following means of evidence: documents, witness depositions, judicial inspection, expert opinions, written reports by public authorities or agencies and interrogation of the parties" [Campbell and Campbell 1995, p. 675]. All these pieces of evidence except the last one are written in nature or require complementary written documents [Campbell and Campbell 1995, p. 675 to 677].

1.2.5 United Kingdom

Historical Background

"English legal history begins in 1066 when the Normans under William I dealt a crushing defeat to the Anglo Saxons in the battle of Hastings [. . .]. One of the great achievements of William I and his successors was the construction of a tight, integrated, and rather simply organized feudal system, with the King as supreme feudal overload [. . .]. The tax system was put on a new basis by William I [and fiscal reasons] underlay the increasing intervention by the central royal administration in civil and criminal law [. . .]. The most important taxpayers were the biggest landowners. The Curia Regis therefore had an interest in private legal disputes over large estates [. . .]. In this way royal justice developed in the twelfth and thirteenth century from a special jurisdiction for affairs of state into a general jurisdiction of wide coverage; in consequence there gradually developed out of the Curia Regis three permanent central courts, staffed by professional judges, capable of acting in the absence of the King, and fixed at Westminster; their jurisdiction was established by 1300 and lasted unaltered into the seventeenth century" [Zweigert and Kötz 1998, p. 182 and 183].

Lawmaking Institutions

Statute-I = 0: "Local rules of law which had come down from the Anglo-Saxon times gradually faded into insignificance beside the law applied by the royal judges. Thus England very early enjoyed a unified law, called for this reason the "common law" [and] there never existed in England one of the essential motor powers behind the idea of codification [. . .]. Litigation in the middle ages was founded on "writs" [. . .], a command of the King directed to the relevant official, judge, or magistrate, containing a brief indication of a matter under dispute" [Zweigert and Kötz 1998, p. 184]. As a result of this particular way in which litigation was initiated, legal practitioners in England start "to think not so much in terms of right as in terms of types of actions, and to interest themselves more in the concrete facts which fell within the various actions or writs rather than elaborating the substantive law into a system based on some rational method" [Zweigert and Kötz 1998, p. 186]. This peculiar background has crystallized into a legal order in which "[l]egislation takes precedence over all other sources of law [but it is] subject to interpretation by the judges and here the influence

of the judiciary upon the development of enacted law may be considerable. [...] Precedents formed by earlier decided cases both define and apply the law. [A judge] is "bound" by an earlier decision only insofar as he himself considers the precedent [...] analogous to the circumstances in issue; he may "distinguish" an earlier authority, and not apply it, in cases where he finds the precedent unhelpful" [David et al. 1995, p. U-69 and U-70].

Adjudication Institutions

"Comparative lawyers tend to assume that in all developed legal systems in the world similar needs are met in ways that are similar, if not precisely the same. The assumption is hard to maintain when one turns to procedural law and compares the preparation and progress of a simple civil suit in the Common Law and the Civil Law, the way the facts are presented, especially the way witness are selected and examined, and the way the different tasks and functions are allocated to the court, the parties, and the lawyers in the different phases of the proceedings. Here the Common and Civil Law have developed quite differently" [Zweigert and Kötz 1998, p. 182 and 183]. "For a little more than a century English civil procedure substantially has been codified. Rules of the Supreme Court, originally authorized by the Judicature Acts 1873, now regulate most civil proceedings in the magistrates' courts and for criminal proceedings in the magistrates' courts" [Glendon et al. 1999, p. 236]. Along with the Judicature Acts, the statutes regulating the English Civil Procedure are: the "Civil Jurisdiction and Judgement Act" 1982 (CJJA, 1982), "The Civil Jurisdiction and Judgements Act" 1991 (CJJA, 1991) giving effects to the "Lugano Convention" and the "Civil Procedure rules" 1998 (CPR, 1998) and devoted to the Courts of Appeal, High Court of Justice, and County Courts, and the Woolf's interim and final (1996) "Reports on civil cases in England" (see Campbell and Campbell, [1995]; Ward and Wragg, [2005]).

Comprehensive-Appeal-I = 0: "In the early development of the common law, an appeal lay only to challenge an error on the record. [...] A review of the judgment, alleging that the court reached the wrong conclusion, later was recognized in the Chancery Court. Thus appeals developed as part of the common law, recognized not by statute, but in the same manner as other rights, as the "the way things ought to be" by the judiciary. When the rural courts were abolished, the right of appeal was incorporated into the statutory framework, the primary source being the Judicature Acts" [Glendon et al. 1999, p. 254] and in particular

part 4 Order 56 to 76 (see Stephen, [1875]). "An appellant now seeks to reverse the judgement as incorrect in law, which in a narrow sense constitutes a rehearing since the judgement may be reversed or some other judgement substituted. But it is not a rehearing in the manner of the rehearing of a civil law appellate court, where new evidence will be heard and there is a denovo proceeding. New evidence may be presented in the English appeal, but it very rarely is, and is accepted only where it could not have been obtained for use at the trial, suggests an important influence on the result and seems reasonably creditable" [Glendon et al. 1999, p. 254 and 255]. The last three grounds were precisely detailed in the judgement of Denning L. J. In Ladd vs. Marshall (1954) 1 W.L.R. 1489 and, also, for similar points, Crook v. Derbyshire (1961) and Mulholland vs. Mitchell (1971) A.C. 666 (see Campbell and Campbell, [1995]). The statutes implemented to reform the civil procedures established by the Judicature Acts have not affected the main principle of "appeal by way of rehearing."

Judgment-Law-I = 0: "Toward the end of the fourteenth century the legal creativity of the royal courts gradually began to wane. It became clear that the procedure of these courts was in many respects too crude and formalistic and that the applicable law was too rigid and incomplete; suits were being lost because of technical errors, because witnesses had been bribed, because of tricks of procedure, or because of the opponent's political influence. Thus as early as the fourteenth century parties who had lost a lawsuit in the King's courts on one of these grounds or who could not obtain an appropriate writ petitioned the King for an order compelling his adversary to do as morality and good conscience, if not the strict rules of the Common Law, required. The King used to transmit such petitions to his highest administrative official, the Chancellor. The Chancellor had an intimate knowledge of the Common Law and its remedies since it was he who issued the writs, and as the "keeper of the King's conscience" and a prominent churchman he was best fitted to judge whether in the particular case the petitioner ought to receive the favor he sought "for the love of God and in the way of charity." In time these petitions were addressed directly to the Chancellor and the decisions he made developed into a complex of special rules of law which are still referred to in England, as they have been ever since the fifteenth century, as equity" [Zweigert and Kötz 1998, p. 187]. "Throughout the fifteenth century the chancellor decided more or less as he thought fit; decisions were consequently much colored by the individual preferences of the

churchman then in office. But after 1529 when the first secular Lord Chancellor, Thomas More, was appointed, equity jurisdiction began to follow the model of the Common Law and developed rules and doctrines, originally in a very fluid and uncertain form, to which the chancellor had recourse when similar fact-situations arose. Regular publication of the Chancellor's decision began towards the end of the sixteenth century and before long he felt almost as bound by precedent as the judges of the common Law courts. His activity came to be seen as being more and more judicial, and his office became the separate Court of Chancery; at the outset the Chancellor was the sole judges, but from 1730 onwards he was helped by his immediate subordinate, the Master of the Rolls. In the eighteenth century it was beyond doubt that "equity" [was] fixed by the decisions and as much formed into technical legal rules as the rules of the Common Law" [Zweigert and Kötz 1998, p. 188].

Later on, order 24 of part 2 of the Judicature Acts consolidated "the area of Common Law (in the narrow sense) and equity. This means that all divisions of the High Court as well as the Court of Appeal must apply all the rules and principles of English law, regardless of whether they were developed "at law" or "in equity" [...]. Today, the Queen's Bench division itself can and must test the "equitable defence" and, if it is established, reject the plaintiff's claim. The conflict between "law" and "equity" which is theoretically possible, is resolved by the Judicature Act, s. 25 (II), which lays down that the rules of equity shall prevail" [Zweigert and Kötz 1998, p. 199]. Courts still "apply both rules of common law and equity, irrespective of their origins, and it is often unclear to which of the two historical systems a rule owes its parentage. The effect of equity upon substantive law is therefore beyond doubt, particularly in relation to matters affecting property and trust, the law of contract, and important remedies like the injunction" [Ward and Wragg 2005, p. 6].

Inquisitorial-I = 0: "When the Anglo-American trial starts, the attorneys will be carefully prepared for it, but the judge will have only the vaguest idea of what the issue and testimony may be. He is supposed to rely on the attorneys to present all the necessary facts and law by word of mouth. How the presentation is effected is a matter for the attorneys: they alone decide which witnesses to call, how many, and in what order; it is the attorneys also who question the witnesses, each witness after the examination by "his" side being "handed over" for cross-examination to the other side" [Zweigert and Kötz 1998, p. 271

and 272]. These rules were not subverted by those provisions of the Judicature Act and related Rules directed toward an increased exercise of the directive power or to the course of procedure and trials, i.e., Order 26, 33, 34, 35 and 36. The first one prescribes that "where it appears to a judge that the statement of claim or defence does not sufficiently define the issue of fact in dispute between the parties, he may direct the parties to prepare the issues; and such issues shall, if the parties differ be settled by judges" [Finlason 1877, p. 297]. However, the norm along with the other three allowing the judge to refer particularly complex matter to an official referee (Order 33), direct question of law to be raised for the opinion of the court (Order 33), order that different questions of fact arising in the case to be tried by different modes of trials (Order 33), refer only to the modes of evaluation of the evidence. They did not allow however the after-Acts judge to freely request or take evidence that was not requested, offered or introduced by the parties or to refuse to collect or admit evidence requested by the parties. In Finlanson's (1877) words, "nothing is so important in procedure as the exercise of directive power in the judicature; that is a power to direct the proper course of procedure. [Yet, in the Common Law this] principle is still not consistently or effectively carried out; and, on the contrary, under the latest measure on the subject [the Judicature Acts], there has actually been retrogression" [Finlason 1877, p. 295 and 296]. The situation remained almost the same until the Woolf's report, who stressed that "[t]he conduct of civil litigation has, historically, been in the hands of the parties, operating within a framework of rules of court, creating the potential for delay [...]. The Woolf Interim Report observed that the whole process could be much like a battleground, with the parties waging "war" often devoid of judicial intervention or control" [Ward and Wragg 2005, p. 426 and 427]. The main by-product has been a significant delay in the High and County courts processes. According to the Woolf's analysis, "in the majority of cases the delay arose from failure to progress the case efficiently, [...] excessive discovery, the use of experts in heavy demand, and the exertion of partisan pressure on experts" [Ward and Wragg 2005, p. 427]. As a result, the CPR (1998) has established that "the judge can freely request or take evidence that has not been requested, offered, or introduced by the parties [and] can refuse to collect or admit evidence requested by the parties, if she deems it irrelevant to the case" [Acartürk et al. 2005, p. 851]. Therefore, *Inquisitorial-I* switched to 1 in 1998.

Written-Evidence-I = 0: "All trials, civil as well as criminal, take the form of a single continuous oral hearing, lasting many hours or even days, if necessary [...]. This has two consequences: every attorney (in England, solicitor) must interview "his" witnesses before the trial and ascertain exactly what they will say [...]. Secondly, each party has the right under Anglo-American procedural law to make the other party disclose information and produce documents relevant for the forthcoming trial" [Zweigert and Kötz 1998, p. 271 and 272]. "When the Anglo-American trial starts, the attorneys will be carefully prepared for it, but the judge will have only the vaguest idea of what the issue and testimony may be. He is supposed to rely on the attorneys to present all the necessary facts and law by word of mouth" [Zweigert and Kötz 1998, p. 271 and 272]. These broad rules have been stated, for the first time, by the Judicature Acts: "It rather appears to be intended, and indeed, in terms, it is provided, that were the pleadings pursue their regular course and raise issues of fact, the trial, unless both parties consent (or unless it is referred) must be by jury" [Finlason 1877, p. 321] and "if there is a trial, whether before a judge with assessor [...] without a jury [...] or with a jury, as usually in the Common Law courts; or by a referee the evidence is taken orally before the court" [Finlason 1877, p. 325]. Moreover, "in modern times [the one of the Judicature Acts] the practice has been to send all case indiscriminately to a jury, and to sift them at the trial" [Finlason 1877, p. 327] and "in case tried by jury, open and oral examination of witnesses necessarily follows" [Finlason 1877, p. 333]. Thus, according to the provisions of the Judicature Acts, most of the evidence, including documentary evidence, is presented at oral hearings before the judge and even in "cases tried before a judge it would proper for him to allow such model of proof as satisfied him, whether by affidavit or deposition, with power, either at the instance of the parties [Order 32 and 36] or his own motion, to require oral examination in open court" [Finlason 1877, p. 333]. Over time, there has been a marked decline of the jury trial in civil cases. "The real turning point was the Administration of Justice [...] Act 1933, which limited the right to claim trial by jury in a civil action to cases of libel, slander, malicious prosecution, false imprisonment, seduction, breach of promise of marriage and fraud" [Ward and Wragg 2005, p. 6]. This reform dramatically increased the relevance of the written means of evidence to the point that, for the colonies whose independence is subsequent to 1933, *Written-Evidence-I* = 1.

2 Transplants

2.1 Transplants to which Statute Law was Transplanted

2.1.1 Albania

"After the First World War the newly created state soon proceeded to codify private law. Mainly under the influence of the Italian law, a Civil Code was enacted in 1928 [...] and a Commercial Code in 1932. Both remained in force until 1945, and the institutions definitely ceased to be used upon the codification of civil law in socialist Albania" [David et al. 1995, p. A-10]. Judicial decisions are not even mentioned among the sources of law in the David et al.'s (1995) report [see David et al. 1995, p. A-9]. Thus, *Statute-I* = *Statute* = 1.

2.1.2 Algeria

"By the end of the colonial period [1830-1958], French law was applicable not only as the personal law of the French community but also as the general law in many fields where the retarted development of Islamic law meant that it could not be applied, such as in criminal law, labour law, most commercial matters [...]. A great number of French laws and regulation governing the most diverse topics were in force when the country achieved its Independence. To avoid considerable lacunae a law of 31 Dec 1962 [...] provides for the continued application of existing legislation with the exception of those provisions which are contrary to national sovereignty, or are discriminatory and colonialist" [David et al. 1995, p. A-21]. Court decisions might only clarify obscure features of the Islamic law, i.e., family law [see David et al. 1995, p. A-21]. Therefore, *Statute-I* = *Statute* = 1.

2.1.3 Argentina

"Argentina [...] acquired its independence from Spain in 1816 [...]. Argentina's first civil code was modeled after the Code Napoléon and adopted in 1869 [...]. The Argentine laws of civil procedure also follow [French law] through Spanish influence [...]. The basic structure of Argentine's federal civil procedure follows the Spanish Ley de Enjuiciamiento Civil of 1855 [...]. The provinces of Cordoba and Santa Fe followed the Spanish law of civil procedure of 1882" [Acartürk et al. 2005, p. 7]. Thus, *Statute-I* = 1; *Comprehensive-Appeal-I* = 1; *Judgment-Law-I* = 1; *Inquisitorial-I* = 1; *Written-Evidence-I* = 1. "Judicial decisions

evidently play an important role as a source of law because other cases are frequently invoked in the reasons for a decision. The Supreme Court [...] has attributed [...] a value similar to that of the law [...]. The function of an appeal in Cassation to unify the case law is also fulfilled by the extraordinary appeal [...]. The same end is served by the system of plenary decisions and by the appeal of "inapplicability of the law." Plenary decisions are deliberated by the Courts of Appeal in full session in order to unify contradictory decisions and establish the interpretation of the law when the court deems it convenient on request by one of the parts; these plenary decisions are binding upon judges of lower courts and can only be modified by a new plenary decision. Appeal of "inapplicability of the law" can be brought when the judgment by the Court of Appeal contradicts a previous decision by the same court" [David et al. 1995, p. A-37 and A-38]. Therefore, *Statute* = 0.

2.1.4 Belgium

"At the outbreak of the French revolution and the conquest of the Belgian Provinces by the French armies (1975), the legal situation was extremely confusing. Only Napoléon was to succeed in imposing his codes in Belgium" [David et al. 1995, p. B-16]. "The French codes remained in force during the 15-year Dutch rule and even after Belgium's independence. Since then the Civil Code of 1804 has been considerably modified [...]. In addition, the Commercial Code of 1872 was further developed to accommodate the local conditions and at present it is an amalgam of the French code and Belgian tradition and practice. Following the French Revolution and throughout Napoleon's rule, the French Code of Civil Procedure of 1806 was imposed in Belgium. In order to meet the local needs and conditions, a new Judicial Code (Code Judiciaire) was issued in 1967 and thus, the French procedure model was considerably abandoned" [Acartürk et al. 2005, p. 63]. Thus, *Statute-I* = 1; *Comprehensive-Appeal-I* = 1; *Judgment-Law-I* = 1; *Inquisitorial-I* = 1; *Written-Evidence-I* = 1. "Court decisions and precedents are a source of law only to the extent that they can be used for the clarification or interpretation of an obscure rule of law, or for the solution of a conflict in respect of which no solution can be found in the traditional sources of law. [...] Except for [this] [...] rather exceptional instance [...] precedents are not legally binding in Belgian law" [David et al. 1995, p. B-15]. Hence, *Statute* = 1. The Belgian colonies in the

sample—i.e., Burundi, Congo, Rwanda and Zaire—gained their independence between 1960 and 1962, before the Code Judiciaire (1967) was issued. Hence, the transplanted adjudication institutions are those illustrated in the 1806 French Code of Civil Procedure.

2.1.5 Benin

"Excluding the Constitution [. . .], internal sources of law are divided into two categories. 1. Modern law attaches primary importance to written sources. A number of enactment date from the colonial epoch [1899-1960] and are the result of the French legislation as applied before independence and which have since remained in force by virtue especially of art. 76 of the Constitution of 26 Nov. 1960 [. . .]. The same is true for a number of French codes of which certain provisions have from time to time been adopted: the Civil Code, Commercial Code, Penal Code and the code of Criminal Procedure [. . .]. 2. Tradition law consists of unwritten customs" [David et al. 1995, p. D-3]. Thus, *Statute-I* = *Statute* = 1.

2.1.6 Bolivia

"During colonial times, legal relations between persons were regulated by the provisions of Spanish law, in force in America with the consequent amendments imposed by the new setting. The most important sources were Las Partidas, the New and Newest spanish Compilation (Nueva y Novissima Recopilacion) and the Compilation of the Laws of the Indies. Once independence was achieved and the Republic established in 1825, Bolivia was the first country in South America to issue its own civil code which came into force on 22 March 1831 and still governs with some important amendments. It was inspired by the French civil code of 1804 to such an extent it may be considered as a translation of it with the exception of the chapter on inheritance taken from the Spanish Newest compilation" [David et al. 1995, p. B-33]. Thus, *Statute-I* = 1; *Comprehensive-Appeal-I* = 1; *Judgment-Law-I* = 1; *Inquisitorial-I* = 1; *Written-Evidence-I* = 1. "Court decisions of national importance are those issued by the Supreme Court of Justice and although not compulsory they are used as directives by the lower courts" [David et al. 1995, p. B-33]. As a result, *Statute* = 1.

2.1.7 Brazil

Brazil "acquired its independence from Portugal in 1822 [. . .]. Portuguese law, which applied to Brazil during the colonial period, was based on Roman sources, customary law,

and medieval Castilian law [...]. The Brazilian civil code was adopted in 1917 [...]. This code is still in force until today [...]. The commercial code was first adopted in 1850 [...]. It followed Portuguese and Dutch sources alongside the usual French and Spanish commercial codes. The Brazilian laws of civil procedure also follow [French law] through the Portuguese influence" [Acartürk et al. 2005, p. 99]. Thus, *Statute-I* = 1; *Comprehensive-Appeal-I* = 1; *Judgment-Law-I* = 1; *Inquisitorial-I* = 1; *Written-Evidence-I* = 1. "The role of case law in Brazil is reduced to that of an interpretative element [and is not a] formative element of legal decisions" [David et al. 1995, p. B-46 and B-47]. Thus, *Statute* = 1.

2.1.8 Bulgaria

"After the Russo-Turkish war of 1877-1878, the Principality of Bulgaria was established north of the Balkan mountains by the treaty of Berlin [...]. The Principality adopted the so-called Turnovo Constitution (1879) which was drawn up after the Belgian model. It was also applied de facto in Eastern Rumelia when in 1885 the region was annexed to the Principality [...]. New laws were borrowed chiefly from the French Code either directly or, more often, via the Italian Civil code of 1865 [...]. The Code of Civil Procedure was adopted from Russia" [David et al. 1995, p. B-64] and so followed Roman law [Acartürk et al. 2005, p. 670]. The socialist experience did not change the basic adjudication procedures [see Acartürk et al. 2005, p. 107]. Thus, *Statute-I* = 1; *Comprehensive-Appeal-I* = 1; *Judgment-Law-I* = 1; *Inquisitorial-I* = 1; *Written-Evidence-I* = 1. "Customs is not a source of law in Bulgaria, not are court decisions" [David et al. 1995, p. B-63]. Hence, *Statute* = 1.

2.1.9 Burkina Faso

Burkina Faso acquired independence from France in 1958 (Britannica, 2015). "Internal sources [of law], apart from the Constitution [...] may be divided into [...] modern or traditional law. 1. Modern law is created by the enactment of legislative provisions, and the statutory material promulgated in this way is of a pretty varied character. Many of these enactments date from before independence; although they came into operation during the colonial period, they continue to have effect so long as they are not repealed by new provisions [...]. 2. Traditional law stems from unwritten customs which are based on the precept of the Koran [...]. Conflicts between the two regimes were resolved in favor of

modern law" [David et al. 1995, p. U-167]. As a consequence, *Statute-I* = *Statute* = 1.

2.1.10 Cameroon

"In Cameroon, [...] a fundamental distinction must be made with regard to the sources of law. Customary law continues to coexist with modern law [...]. Modern law, however, has been enriched successively by various changes in the constitutional development of the country [...]. In East Cameroon, this legislation consisted either of French laws extended to the territory, possibly with adaptations, or of laws that were peculiar to Cameroon [...]. In West Cameroon, the policy of the British government had been to establish a system of law identical to that in Nigeria [...]. In area where the law has not been unified, there exists a plurality of sources of law which creates the problem of delimitations of statutes and conflicts of jurisdictions in matters involving people of different status. There are very few, if any, rules for resolving such situations. There is also a dearth of decided cases on the matter. Thus, when situations of this nature arise, they can best be dealt with by references to the studies on the subject" [David et al. 1995, p. C-5]. Thus, *Statute-I* = 1; *Statute* = 1.

2.1.11 Central African Republic

Central African Republic was under French control from 1890 to 1960 (Britannica, 2015). "If we put aside diplomatic treaties, which constitute the principal international source of law, as well as constitutional provisions determining the organization of the government, the sources of law fall into two categories. 1. Modern law is above all based on written sources. Pursuant to the Constitution of 25 Nov. 1960 art. 45, the legislative texts dating from the colonial period have been maintained in force together with all earlier legislation [...]. 2. Customary law is based on unwritten customs [...]. In case of a conflict of customs stemming from legal relations between persons of different status the modern law prevailed" [David et al. 1995, p. C-39]. Therefore, *Statute-I* = *Statute* = 1.

2.1.12 Chad

Chad was a French colony from 1920 until 1960 (Britannica, 2015). "If the Constitution [...] is put on one side, the internal sources can be divided into two categories, depending on whether are sources of modern law or traditional law. 1. Modern laws result from a rather heterogeneous collection of enacted laws. A number of them date from the days before

independence: brought into operation during the colonial period they retain their authority in so far as they have not been repealed [...]. There is no collection of decisions given by Chad courts. 2. Traditional law results from unwritten customs [...]. In case of legal relationship involving both laws, the conflict was resolved in favor of modern law" [David et al. 1995, p. C-45 and C-46]. As a consequence, *Statute-I* = *Statute* = 1.

2.1.13 Chile

Chile "acquired its independence from Spain in 1810 [...]. After independence in 1810, a first provisional constitution was adopted in 1812. The first democratic constitution came into force in 1833 [...]. Major codifications began in the 1840s. One of the most prominent pieces of Latin American legislation is the Chilean civil code of 1855. The code followed the Roman law, the French civil code of 1804, ancient Spanish compilations, the Louisiana, Austrian, Portuguese, Dutch, and the former Sicilian and Sardinian civil codes, a draft of a Spanish civil code (never enacted), and French, Spanish and German legal doctrine. The civil code of 1855 has undergone many amendments and changes over the past 150 years and is still valid today. The Chilean laws of civil procedure also follow [French law] through the Spanish influence" [Acartürk et al. 2005, p. 143]. Thus, *Statute-I* = 1; *Comprehensive-Appeal-I* = 1; *Judgment-Law-I* = 1; *Inquisitorial-I* = 1; *Written-Evidence-I* = 1. "Judicial decisions do not constitute a source of law" [David et al. 1995, p. C-51]. Thus, *Statute* = 1.

2.1.14 Colombia

Colombia "acquired its independence from Spain in 1810 [...]. During the colonial period, Spanish legislation was in force and remained valid well into the second half of the 19th century. Under the constitution of 1886, [...] new codifications were soon adopted. Colombia's civil code was adopted in 1873 as a modification of the Chilean civil code of 1855, itself modeled after Code Napoléon of 1804 and on other Roman law sources. The civil code of 1873 was further amended in 1887. The commercial code of the former province of Panama became, with appropriate additions, the commercial code of Colombia in 1887, and followed the Spanish commercial legislation. This code was replaced by a new commercial code in 1971. The Colombian laws of civil procedure also follow [French law] through Spanish influence [...]. The current code of civil procedure was adopted in 1970 and was revised in

1989" [Acartürk et al. 2005, p. 159]. Thus, *Statute-I* = 1; *Comprehensive-Appeal-I* = 1; *Judgment-Law-I* = 1; *Inquisitorial-I* = 1; *Written-Evidence-I* = 1. "The decisions of the Supreme Court and Council of State are used as guide by judges of lower courts, but are not binding" [David et al. 1995, p. C-66]. Thus, *Statute* = 1.

2.1.15 Comoro

Comoro became independent of France in 1978 (Britannica, 2015). "Before independence French law and the law of Comoro, consisting of Muslims law and local customs, were both in use [...]. French law as applied by the French courts was largely the same as the law of metropolitan France [...]. All legislation and regulations which were in force in the islands before independence remain in force in Comoro until contrary rules are enacted" [David et al. 1995, p. C-77]. Court decisions are not mentioned among the sources of law listed in the David et al.'s (1995) report [see David et al. 1995, p. C-77]. Thus, *Statute-I* = *Statute* = 1.

2.1.16 Congo, Republic of the

The Republic of Congo gained independence from France in 1960 (Britannica, 2015). "Basic Act of 5 April 1977 art. 28 affirms the principle of continuity of the national law: existing laws remain effective, unless they conflict with the Basic Act or are abrogated or modified. The following French codes which were in force on 28 Nov, 1958 are still in effect, except in so far as they have since been modified by the Congolese legislator: the Civil Code, the Code of Civil Procedure [...]. Judicial decisions are an important source of law because they are cited as precedents" [David et al. 1995, p. C-82]. Indeed, the Supreme Court acts as a Cassation court and has "a general power of control over the legal acts of governmental and judicial authorities" [David et al. 1995, p. C-82]. So, *Statute-I* = 1; *Statute* = 0.

2.1.17 Costa Rica

Costa Rica joined other Central American provinces in 1821 in a joint declaration of independence from Spain (Britannica, 2015). Until "1841 civil relations in Costa Rica continued to be governed by the rules in force during the colonial times [...]. In 1841, a code was enacted later know as the General Code because it included civil, penal and procedural matters. The section devoted to private law was inspired by the Code Napoléon, except in matrimonial matters, which were taken from Canon law. The General Code continued to

be in force until 18 Jan 1888, when the present Civil Code came into force" [David et al. 1995, p. C-89]. "The first code of civil procedure was adopted in 1887 and was inspired by the French and Spanish codifications. The current code of civil procedure was adopted in 1989. The first commercial code was adopted in 1853, and followed closely the Spanish commercial code of 1829. This code was replaced by the current commercial code, in force since 1964" [Acartürk et al. 2005, p. 168]. Thus, *Statute-I* = 1; *Comprehensive-Appeal-I* = 1; *Judgment-Law-I* = 1; *Inquisitorial-I* = 1; *Written-Evidence-I* = 1. "Judicial decisions are not a formal source of law but the judgments of the Cassation division have a noticeable influence on court decisions" [David et al. 1995, p. C-89]. Since the binding effect of these decisions is significantly less powerful than that of the rulings of the Cassation courts of Argentina, Dominican Republic, Guatemala, Indonesia, Tunisia, and Turkey, *Statute* = 1.

2.1.18 Cote D'Ivoire

Cote d'Ivoire was a French colony between 1893 and 1960 (Britannica, 2015). "Cote d'Ivoire's legal system is based on the French [law]. Initially, the French introduced their civil and commercial codes, which were applicable only to French citizens, while the rule of African customary law and Islamic law applied to the native Africans and Muslims. After independence, Cote d'Ivoire initiated revision of the imposed French law, although it followed the French system and model of codification. Under the Constitution that was adopted in 1960, all legislation in effect during the colonial period remained in force in the new republic as long as it did not contradict the Constitution. As a result, a number of laws governing personal and family matters were adopted in 1964. These laws together with the law of obligations, which was taken from the French civil code, serve as a civil code law and in the area of commercial law. Cote d'Ivoire's Code of Civil Procedure was adopted in 1972 and it was last modified in 1993 and in 1997. The procedural code also follows [French law]" [Acartürk et al. 2005, p. 175]. So, *Statute-I* = 1; *Comprehensive-Appeal-I* = 1; *Judgment-Law-I* = 1; *Inquisitorial-I* = 1; *Written-Evidence-I* = 1. "[C]ase law and the writings of legal authors have persuasive authority only" [David et al. 1995, p. I-120]. So, *Statute* = 1.

2.1.19 Croatia

"The earliest written source of law was the Code of Vinodol [...] from 1288, which had

limited effect as Croatia fell under the legal systems of Hungary and Austria. However, the Austrian Civil Code also did not gain much prominence because of Croatia's long-standing association with Hungary. After World War I, a new Kingdom of Serbs, Croats, and Slovenes was established, which was renamed Yugoslavia in 1931. During the socialist period, the legal system fused with the family of the socialist legal system. After independence in 1991 and the ensuing military action, Croatia has started reforming its legal system. A new constitution was adopted in December 1990. A new code of civil procedure was passed in 1996" [Acartürk et al. 2005, p. 184]. Hence, *Statute-I* = 1; *Comprehensive-Appeal-I* = 1; *Judgment-Law-I* = 1; *Inquisitorial-I* = 0.5; *Written-Evidence-I* = 0; *Statute* = 1.

2.1.20 Cuba

"Since Cuba had been a Spanish colony for nearly four centuries [1511-1898], its law was largely Spanish and still remains so in part. Shortly before independence, the then new Spanish Civil Code of 1889 was extended to Cuba by a Decree of 31 July 1889. The Civil Code (CC) was maintained by the Republic by a Decree of the Military Governor on 1 Jan. 1899 and later by Regulation no. 148 of 13 May 1902. The other basic source of private law which still remains in force is the Spanish Commercial Code of 1885 [...], promulgated in that country in 1886 and in Cuba in the same year" [David et al. 1995, p. C-97]. Judicial decisions are not even mentioned among the sources of law listed in the David et al.'s (1995) report [see David et al. 1995, p. C-97]. Thus, *Statute-I* = *Statute* = 1.

2.1.21 Czech Republic

"While until the 15th century, Roman canon law had little impact over Czech legal development, the fall under Austrian rule led to the establishment of Roman Catholic influence and Roman law. During the 18th and 19th centuries, Austrian legislation was firmly established in Bohemia and Moravia, particularly with the Habsburg legislation (the Penal Code of 1707 and the Austrian General Civil Code of 1811). Thus, up until the 20th century, Czech legal development followed the Roman-Germanic [law]. The country acquired its independence from the Austrian Empire in 1918, when Czechoslovakia was formed. After the defeat of the Austro-Hungarian Empire in 1918, the Czech and Slovak parts united to form Czechoslovakia. Since Slovakia had been under Hungarian legislation and the legal traditions

of the two differed, each province continued to apply the legislation each had inherited from the Austro-Hungarian Empire. After German occupation in 1939, Austrian and German laws were applied in Czechoslovakia. After the consolidation of Communist rule in 1948 new socialist legislation was adopted. A civil code came into force in 1950, following the tradition of the Soviet Civil Code of 1922. The 1950 civil code was later replaced by a new code in 1964, which was subsequently amended. A substantial body of laws was adopted in the 1960s, covering areas of civil and criminal procedure, family law, economic, and labor law. [...] The civil code from 1964 has been amended substantially in 1991 and 1995. A new commercial code came into force in 1992. The code of civil procedure from 1963 is currently valid with 42 amendments up to 2000 inclusive" [Acartürk et al. 2005, p. 199]. Therefore, *Statute-I* = 1; *Comprehensive-Appeal-I* = 1; *Judgment-Law-I* = 1; *Inquisitorial-I* = 0.5; *Written-Evidence-I* = 0. "[T]he decisions of courts have only an effect inter partes and do not constitute binding precedents" [David et al. 1995, p. C-114]. Thus, *Statute* = 1.

2.1.22 Dominican Republic

It "acquired its independence from Haiti in 1844 [...]. The legislation of the Dominican Republic follows closely the French codes of the early 19th century [...]. From 1795 until 1844, the French and then the Haitians imposed French-inspired legislation [...]. After independence, the five main French codes of 1804-1811 were adopted in French as the law of the Dominican Republic. These amended and reformed commercial and civil codes are still in force today [...]. The Dominican laws of civil procedure follow the French legal model [...]. The current code of civil procedure was also adopted in 1884 and has been extensively reformed" [Acartürk et al. 2005, p. 215]. Hence, *Statute-I* = 1; *Comprehensive-Appeal-I* = 1; *Judgment-Law-I* = 1; *Inquisitorial-I* = 1; *Written-Evidence-I* = 1. "Decisions made by the Supreme Court of Justice are published monthly in the Boletin Judicial and these decisions become a binding precedent" [David et al. 1995, p. D-38]. Thus: *Statute* = 0.

2.1.23 Ecuador

"The Spanish discovered and conquered Ecuador in 1532-1534. After independence in 1822, Ecuador was organized as a department of the Republic of Gran Colombia, comprising also Colombia and Venezuela. In 1830, the Quito province seceded from the federation along

with Venezuela to form an independent Republic of Ecuador. According to the Constitution of 1823 Ecuador inherited all the Spanish legislation not conflicting with the Constitution. Ecuador adopted a revised version of the Chilean Civil Code of 1855 and promulgated it in 1860. The Civil Code was substantially revised in 1869 and amended further in 1871 and 1879. The Chilean civil code followed the civil code of 1860-1869, which followed the French civil code of 1804, ancient Spanish compilations, the Louisiana and Austrian civil codes, a draft for a Spanish civil code (never enacted), and French, Spanish, and German legal doctrines. The Ecuadorian laws of civil procedure also follow [French law] via Spanish influence. Five codes of civil procedure were adopted between 1825 and 1863 and copied Spanish procedural legislation. The first proper code of civil procedure entered into force in 1869 and was based on the Peruvian code of civil procedure of 1853. Subsequent re-codifications of civil procedure followed in 1879, 1938, and 1960" [Acartürk et al. 2005, p. 222]. "The Spanish Commercial Code of 1829 [...] was of great use in drawing up the earlier Ecuadorian Code of 1878" [David et al. 1995, p. E-3]. As a result, *Statute-I* = 1; *Comprehensive-Appeal-I* = 1; *Judgment-Law-I* = 1; *Inquisitorial-I* = 1; *Written-Evidence-I* = 1. "Ecuador has a system of written law [...]. The Supreme court meeting in Plenary Session has the power—in the case of contradictory decisions on the same point of law—to set forth an annulling rule to govern future cases, and it is generally compulsory unless the law provides to the contrary" [David et al. 1995, p. E-3]. Since Supreme court decisions cannot modify statutes and so are significantly less binding than the rulings of the Cassation courts of Argentina, Dominican Republic, Guatemala, Indonesia, Tunisia, and Turkey, *Statute* = 1.

2.1.24 Egypt

Egypt "acquired its independence from the United Kingdom in 1922 [...]. Even though the United Kingdom occupied the region from the 1880s to the 1920s and exercised a great influence, the Egyptian legal system was built on the Napoleonic Code with impact by Shari'a. Modern Egyptian legal history is considered to have commenced during the second half of the 19th Century, which witnessed a movement towards French Law. The civil code, commercial code, the maritime law, as well as the civil and commercial procedures laws were adopted in 1883. All were based on French Law. The current Civil Code was issued in 1948

and is principally oriented towards French law, but omits family and inheritance law. It reflects the influence of the French and Italian civil codes and many ideas developed by the French courts, as well as the principles of Shari'a. A new commercial code came into force in 1999 and replaced [that of] 1883. The current Egyptian Code of Civil and Commercial Procedures was issued in 1968 ("CCCP") and follows [French law. It] is applied except in cases of personal status, where a special law on procedures in personal status matters has been issued in 2000" [Acartürk et al. 2005, p. 230]. So, *Statute-I* = 1; *Comprehensive-Appeal-I* = 1; *Judgment-Law-I* = 1; *Inquisitorial-I* = 1; *Written-Evidence-I* = 1. "Case decisions are not at all a formal source of law. Following French Law, Egyptian law considers them to have persuasive authority only" [David et al. 1995, p. E-13]. So, *Statute* = 1.

2.1.25 El Salvador

El Salvador "acquired its independence from Spain in 1821 [...]. Spanish colonial laws however were applied during the time of the Central American Confederacy. The first Salvadoran codifications took place in mid-19th century and closely followed the Spanish codes of the previous decades. A civil code was adopted in 1858 and was substantially modified in 1880s. The civil code is still in force today following numerous amendments. Family law has been removed into a separate code in 1993. In addition, a separate labor law came into force in 1963. Commercial code came into force in 1855 and was replaced by a new compilation in 1904, using as a model the Spanish commercial code of 1882 as well as elements of the respective Chilean, Italian and Portuguese codifications. The current commercial code was adopted in 1970 and has been several amendments since then. The Salvadoran laws of civil procedure also follow the French [model]. The first code of civil procedure was adopted in 1857, [...] inspired by the earlier Spanish codification [;] replaced in 1881 and 1882 and is still in force" [Acartürk et al. 2005, p. 238]. So, *Statute-I* = *Statute* = 1; *Comprehensive-Appeal-I* = 1; *Judgment-Law-I* = 1; *Inquisitorial-I* = 1; *Written-Evidence-I* = 1.

2.1.26 Equatorial Guinea

Equatorial Guinea was under Spanish control between 1844 and 1968 (Britannica, 2015). "The Constitution provides that legislation in force at the time of independence and not in contradiction to the Constitution shall remain in force until it is repealed or modified

(Transitory Provision no. 2). Therefore Spanish legislation not contrary to the Constitution remains in force for the time being [...]. [The] main laws in force at the time of independence and therefore of Spanish origin, are the [...] Civil Code of 27 July 1889" [David et al. 1995, p. E-20 and E-21]. Court decisions are not mentioned among the sources of law listed in the David et al.'s (1995) report [see David et al. 1995, p. E-20]. So, *Statute-I* = *Statute* = 1.

2.1.27 Estonia

Estonia "was independent between 1920 until 1940, when it was occupied by the USSR. The country gained re-independence [in 1991]. [...] Soviet legislation was imposed and abrogated the pre-1940 body of laws. After the return to democracy and independence in 1991, the legal system has been reformed along modern, European civil law principles. A new constitution, based on those from 1920 and 1938, was adopted in 1992. A Law on the General Principles of Civil Law was passed in 1994, thus replacing the 1964 Soviet civil code. A new commercial code was adopted in 1995. Current legal reform has been driven by prospective EU membership [...]. The current in force Code of Civil Procedure was passed in 1998" [Acartürk et al. 2005, p. 244]. Thus, *Statute-I* = *Statute* = 1; *Comprehensive-Appeal-I* = 1; *Judgment-Law-I* = 1; *Inquisitorial-I* = 0.5; *Written-Evidence-I* = 0.

2.1.28 Gabon

Gabon was a French colony over the period 1885-1960 (Britannica, 2015). The "sources of law [...] may be divided into two categories. 1. written sources hold a primordial position in modern law. Certain texts dating from the colonial period have been maintained in force with all the previous legislation by const. art. 71; they were either borrowed from the provisions of French law rendered applicable in French Equatorial Africa, such as the Civil code, or they were peculiar to the African territories [...]. 2. The traditional law is made up of unwritten customs" [David et al. 1995, p. G-3 and G-4]. Judicial decisions are not even mentioned among the sources of law listed in the David et al.'s (1995) report [see David et al. 1995, p. G-3 and G-4]. As a consequence, *Statute-I* = *Statute* = 1.

2.1.29 Georgia

Georgia "acquired its independence from Soviet Union in 1991. [...] Soviet Union's legal system and Soviet legislation [had a huge impact] on Georgia's private law from 1921

to 1991 [...]. Georgia's legal unification tradition dates back to the feudal period with Greek-Roman law being the earliest legal influence introduced into the country. After its independence, the government initiated a legal reform to harmonize Georgian law with the European legal traditions and to provide legal conditions for transition to market economy. Georgia's first codification was the Code of Laws of Vakhtang VI. [A] new constitution was adopted in October 1995 and a new Civil Code was drafted by Georgian and foreign civil law jurists that came into force in 1997. The code was modeled after modern European codes, particularly the French, German and Dutch codes and the Swiss law of obligations [...]. Since 1924, Georgia had four procedural codes directing the civil procedure and practice of the courts. In 1997, a new Civil Procedure Code was adopted, in response to the changing legal framework. This Code aims to provide better protection against the breach or dispute of individuals' rights. The code has been frequently changed with the latest modification in 2002" [Acartürk et al. 2005, p. 272]. Therefore, *Statute-I* = 1; *Comprehensive-Appeal-I* = 1; *Judgment-Law-I* = 1; *Inquisitorial-I* = 0.5; *Written-Evidence-I* = 0; *Statute* = 1.

2.1.30 Guatemala

"Spanish colonial legislation was used in the country until its independence [1821], but soon thereafter the Spanish codes, based on the French codes of the early 19th century became the model for Guatemalan legislation [...]. The first Guatemalan codifications took place in the second half of the 19th century and closely followed the Spanish codes of the previous decades. The first civil code was adopted in 1877 and was heavily influenced by the Spanish draft of a civil code from 1851 (itself modeled on the French Code Napoléon of 1804). The civil code also carried elements of the Portuguese civil code of 1867. A commercial code also came into force in 1877 closely following the Spanish commercial codification of mid-19th century. Most Guatemalan codes of the 19th century were revised in the 1930s, and again, in the 1960s. The current civil code was adopted in 1963, but remains based on the principles of the Romanist [law]. The commercial code valid today was enacted in 1970. The Guatemalan laws of civil procedure follow a French legal model with Spanish influence [...]. The first code of civil procedure was adopted in 1877, and inspired by the earlier Spanish codification. This code was substantially revised in 1930s, and a new code of civil procedure was adopted

in 1963" [Acartürk et al. 2005, p. 314]. Thus, *Statute-I* = 1; *Comprehensive-Appeal-I* = 1; *Judgment-Law-I* = 1; *Inquisitorial-I* = 1; *Written-Evidence-I* = 1. "Court decisions on civil and commercial matters are a source of law. Whenever five decisions containing an identical opinion are emitted by the Supreme Court of Justice in Cassation appeals heard buy it, they become legal doctrine [...]. The value of the enforceable judgments of the Courts of Protection [hearing cases on constitutional rights] is also recognized and they may be cited as grounds of law" [David et al. 1995, p. G-69]. Therefore, *Statute* = 0.

2.1.31 Guinea

Guinea was a French colony from 1890 to 1958 (Britannica, 2015). "During the colonial period there existed a legal discrimination between native inhabitants and the European settlers, since the Europeans were subject to metropolitan legislation whilst the natives continued, particularly in civil matters, to be subject to special courts [...]. With independence [...] a Regulation [...] abolished the Customary Courts and made all persons amenable to the same courts applying the same texts" [David et al. 1995, p. G-79]. "[D]ecisions of the Court of Cassation, of the Court of Appeal and of other courts, are not a source of law" [David et al. 1995, p. G-79]. As a consequence, *Statute-I* = *Statute* = 1.

2.1.32 Haiti

At the end of the French rule (1804), "Haiti has adopted, with some modifications, the Civil Code of Napoleon. Voted in 1825, this Code had been in force since 1 May 1826. The other French codes have been similarly adopted, namely the Commercial Code, the Penal Code, the Code of Civil and Commercial Procedure" [David et al. 1995, p. H-4]. Judicial decisions are not even mentioned among the sources of law listed in the David et al.'s (1995) report [see David et al. 1995, p. H-4]. Thus, *Statute-I* = *Statute* = 1.

2.1.33 Honduras

Honduras "acquired its independence from Spain in 1821 [...]. Spanish colonial legislation was used in Honduras until independence, but soon thereafter the French codes of the early 19th century became the model for Honduran legislation. Spanish colonial laws however were applied during the time of the Central American Confederacy. The first Honduran codifications took place well into the second half of the 19th and the early 20th century. A

civil code was adopted in 1906 based on the French Napoleonic Code of 1804. After some amendments, this civil code is still in use today. The family law part of the 1906 civil code was removed and compiled as a Family Code in 1984. A commercial code was enacted in 1898, also following closely the French commercial code, and remained in force until 1940. The current commercial code was adopted in 1949. The Honduran laws of civil procedure also follow the French legal model through the Spanish influence [...]. The first code of civil procedure was adopted in 1906, and inspired by the French and Spanish codifications. This code is still valid today" [Acartürk et al. 2005, p. 324]. So, *Statute-I* = 1; *Comprehensive-Appeal-I* = 1; *Judgment-Law-I* = 1; *Inquisitorial-I* = 1; *Written-Evidence-I* = 1. Judicial decisions, even those of the Supreme Court in constitutional matters, affect only the involved parties and are not binding on other courts [see David et al. 1995, p. H-8]. So, *Statute* = 1.

2.1.34 Hungary

"[L]egal scholars [...] describe the Hungarian legal system prior to World War II as being a system of its own, relying on statutes, customary law, and court decisions, and similar to both the common law system and the Roman-Dutch law system of South Africa. After the war, Hungary adopted a new constitution in 1949 and embarked upon a course of legal reform along socialist legal principles [...]. Since the return to democracy [1989] Hungary has enacted new civil and commercial legislation along Western European legal principles [...]. A new code of civil procedure based on socialist legal principles was passed in 1952, thus replacing the old German-style civil procedure code from 1911. The civil procedure code from 1952 has been further amended in 1990, 1992 and 1995" [Acartürk et al. 2005, p. 340]. As a consequence, *Statute-I* = 1; *Comprehensive-Appeal-I* = 1; *Judgment-Law-I* = 1; *Inquisitorial-I* = 0.5; *Written-Evidence-I* = 0. "The function of the courts is to apply the law" [David et al. 1995, p. H-18]. As a consequence, *Statute* = 1.

2.1.35 Indonesia

Indonesia "acquired its independence from the Netherlands in 1949 [...]. During the colonial period the Dutch failed to achieve a uniform application of Dutch legislation, and allowed the indigenous population to use customary law. However, the Dutch East Indies State Regulation of 1910 established a system whereby Dutch law governed European and

Japanese residents. Foreign Asian groups were governed by their own national legislation, and native Indonesians were subject to customary law (adat). However, for these Asian groups, Dutch law applied in most civil, commercial, and criminal cases. The main codifications based on Dutch legislation, following [French law], were adopted in 1848 and are currently still in force. After attempts at codifying customary law failed, a dual legal system was accepted in 1926, giving customary law an equal status alongside Dutch civil law [. . .]. In 1945, a new constitution was adopted, and with amendments, it is still valid today. After some abrogation of Dutch law during the 1950s, Indonesia regime restored the reliance on the Dutch civil and commercial codes. The civil code has been amended since independence" [Acartürk et al. 2005, p. 358]. "The relevant law of civil procedure at present [1972] in force is the Revised Indonesian Regulation [. . .] of 1848 [. . .]. It differs from the [1806 French Civil Procedure Code insofar as] proceedings are generally carried out orally" [David et al. 1995, p. I-40]. So, *Statute-I* = 1; *Comprehensive-Appeal-I* = 1; *Judgment-Law-I* = 1; *Inquisitorial-I* = 1; *Written-Evidence-I* = 0. "In view of the significant role of unwritten law in the legal system it follows that case law is very important. It continues to be a source of law, especially in relation to customary and conflict of law issues. It is also of importance with regard to the written law [. . .]. The Supreme Court has a leading role to play in the unifying process of case law at present. In practice lower courts consider themselves bound by the decisions of this highest judicial body for otherwise they lay themselves open to being overruled in cassation" [David et al. 1995, p. I-33]. Thus, *Statute* = 0.

2.1.36 Iran

"Although Iran was one of the few Muslim countries to escape colonization, an unfortunate war with the Russian led an Iranian "capitulation" in 1828. The westernization of Iranian society began with the Constitution of 1906 which was copied from Franco-Belgian models. This reform, though profound, was only political. In 1928 the "capitulation" was abolished and the foundations for deep social reform were laid with the promulgation of a series of codes and the organization of the judicial system, along the French model. The failure of these social reforms led to the Referendum of 26 Jan.1963: "The White Revolution" [. . .] The basic source of civil procedure is the Code of Civil Procedure of 17 Sept.

1939 which is largely inspired by French law" [David et al. 1995, p. I-45 and I-52]. "Judicial decisions are not, in principle, a formal source of law. The judge must decide according to Statute and he has no power to elaborate general rules (Code of Civil Procedure art. 5)" [David et al. 1995, p. I-48]. As a consequence, *Statute* = *Statute-I* = 1.

2.1.37 Italy

Italian "civil law, concentrating on private law, owes much of its modern development to other influences, particularly canon and Germanic and feudal law, as interpreted and applied in the Middle Ages and Renaissance. As a result of French occupation in 1796 and from the French revolution, Italy received both the ideology of the revolution and the French Codes. The current civil and civil procedure codes were developed 1942. The civil code combined [...] the regulation of all aspects of private life. It abrogated both the 1865 civil and civil procedural codes and the commercial code of 1882" [Acartürk et al. 2005, p. 382]. Thus, *Stature-I* =1; *Comprehensive-Appeal-I* = 1; *Judgment-Law-I* = 1; *Inquisitorial-I* = 1; *Written-Evidence-I* = 1. "Art.1 of the [...] Civil Code [...] enumerates as sources of law (1) statutes, (2) regulations and (3) customs [...]. The decisions of the Italian courts—even those of the joint sections of the Court of Cassation—have in respect to similar cases, merely persuasive authority" [David et al. 1995, p. I-96 and I-97]. Thus, *Statute* = 1.

2.1.38 Kazakhstan

Kazakhstan "acquired its independence from the Soviet Union in 1991, having been a constituent Soviet republic since 1921 [...]. After independence, there has been a move toward Western-style compilations of law [...]. A new Civil Code of the Republic of Kazakhstan [came into force between 1995 and 1999], thus replacing the civil code of the Kazakh Soviet Socialist Republic from 1963. The new civil code follows the new Russian civil code and exhibits the considerable influence of the German and Dutch civil codes. There is no commercial code; however important laws regarding commercial activity and foreign investment have been adopted. The code of civil procedure of the Kazakh Socialist Republic from 1963 remained valid until July 1999, when the [new] Code of Civil Procedure [...] came into force" [Acartürk et al. 2005, p. 414]. Thus, *Statute-I* = 1; *Comprehensive-Appeal-I* = 1; *Judgment-Law-I* = 1; *Inquisitorial-I* = 0.5; *Written-Evidence-I* = 0; *Statute* = 1.

2.1.39 Khmer Republic

Cambodia wad a French protectorate from 1863 to 1953 (Britannica, 2015). "French laws and regulation made prior to independence are still in force unless superseded by new national legislation" [David et al. 1995, p. K-14]. "At present, there are no reports of judicial decisions" [David et al. 1995, p. K-14]. As a consequence, *Statute-I* = *Statute* = 1.

2.1.40 Laos

From the 19th century until 1954 Laos was under French control (Britannica, 2015). "French texts, promulgated before Laos became fully independent, were, for the most part, kept in force until such time as new legislation could be introduced" [David et al. 1995, p. L-3]. Judicial decisions are not even mentioned among the sources of law listed in the David et al.'s (1995) report [see David et al. 1995, p. L-3]. As a result, *Statute-I* = *Statute* = 1.

2.1.41 Latvia

Latvia gained independence from the Soviet Union in 1991 (Britannica, 2015). "During the 50 years of socialism, the legal system was re-modeled along socialist legal principles. Soviet legislation governed Latvia as a constituent republic. After the return to democracy in 1991, Latvia has moved from the socialist [law] to European civil law. The country has re-instated some of the pre-1940 [law]. Latvia restored its pre-war Constitution from 1922 in 1991. The 1937 Civil Law was also re-introduced in 1992. A code of civil procedure based on socialist legal principles was passed in 1964. A new Law on Civil Process was adopted in 1999" [Acartürk et al. 2005, p. 451]. Thus, *Statute-I* = 1; *Comprehensive-Appeal-I* = 1; *Judgment-Law-I* = 1; *Inquisitorial-I* = 0.5; *Written-Evidence-I* = 0; *Statute* = 1.

2.1.42 Libya

"Until 1951, the basis of the Libyan legal system was Italian law in conjunction with local religious law. Upon becoming an independent state, Libya adopted its own system of law [. . .]. The lawmakers followed Egyptian law, which was extensively adopted in the most important areas such as civil, commercial, and procedural law. Since Egyptian law was, in turn, influenced by French and the more modern Italian law, the Libyan civil law in essence reflects the legal philosophy of the Code Civil. If a problem cannot be solved by reference to

the Civil Code, one must resort to [...]: Islamic law, customary law, natural law, and rules of reasonableness" [David et al. 1995, p. L-35]. Court decisions are not mentioned among the sources of law in the David et al.'s (1995) report. Thus, *Statute-I* = *Statute* = 1.

2.1.43 Liechtenstein

"Austrian legislation was introduced into Liechtenstein by means of a Prince's Warrant" [David et al. 1995, p. L-42]. Case law is not mentioned among the sources of law in the David et al.'s (1995) report [see David et al. 1995, p. L-42]. So, *Statute-I* = *Statute* = 1.

2.1.44 Lithuania

Lithuania "acquired its independence in 1918 having been part of the Russian Empire since 1795 and under German occupation from 1915 until 1918. Lithuania was independent between 1918 until 1940, when it became part of the USSR. The country re-gained independence from the Soviet Union in 1991. [...] Since 1990, however, the legal system has been reformed to meet the demands of the social and economic changes brought about by a return to democracy. Thus, socialist civil code from 1964 was first amended through a series of legislation and then entirely replaced by the new Civil Code in 2001. [The] code of civil procedure [...] has undergone substantial changes since 1990" [Acartürk et al. 2005, p. 466]. As a consequence, *Statute-I* = 1; *Comprehensive-Appeal-I* = 1; *Judgment-Law-I* = 1; *Inquisitorial-I* = 0.5; *Written-Evidence-I* = 0; *Statute* = 1.

2.1.45 Luxembourg

"Luxembourg's legal system originated as a mixture of unwritten customary and local legislation. The earliest influences date back to the 16th century when Luxembourg was part of the Habsburg Empire, governed either from Brussels or Vienna. After the French revolution, the French, Dutch and Belgians successively controlled the country and imposed their respective legislation [...]. The Civil Code, adopted in 1807, was drawn up similar to the French code. Even though the code was substantially modified in 1898, its orientation remained intact. In commercial law, Luxembourg initially embraced [French law] through the French commercial code introduced in 1807. However, at present commercial and corporate law has taken a slightly different direction by incorporating elements of German law. Both the Civil Code and the Commercial Code were modified in 2000. The civil procedure and

practice of courts also follow [French law]. Luxembourg's Code of Civil Procedure of 1806 was modeled on the basis of the French code. Following recent changes it also acquired some features of the modern Belgian legislation" [Acartürk et al. 2005, p. 475]. Thus, *Statute-I* = 1; *Comprehensive-Appeal-I* = 1; *Judgment-Law-I* = 1; *Inquisitorial-I* = 1; *Written-Evidence-I* = 1. "Judicial decision only has a legal effect for the particular case in which it was made. The authority of precedents, as far as future decisions are concerned, is purely intellectual and not juridical" [David et al. 1995, p. L-50]. Thus, *Statute* = 1.

2.1.46 Madagascar

Madagascar was a French colony over the period 1883-1960 (Britannica, 2015). "Colonization brought with it deep changes in the field of private law [...]. French law was to infiltrate the Madagascan legal system on a large scale. French rules [...] were used to reform the traditional institutions [...]. Independence has put an end to this situation. Madagascar has in fact determined to set about unifying her law" [David et al. 1995, p. M-5]. "The internal sources of law are ranked in a hierarchy which includes Statute law, the general principles of law, customs and traditions [...]. As in the French system, case law and academic writing are not true sources of law but simply authorities which help the judge to interpret the law" [David et al. 1995, p. M-4 and M-5]. Thus, *Statute-I* = *Statute* = 1.

2.1.47 Mali

Mali was a French colony until 1960 (Britannica, 2015). There are two "sources of law [...]. 1. In modern law primary importance is attached to written sources of law. Certain provisions of law dating from the colonial period have been kept in force by Const. art 51 together with the whole of the earlier legislation [...]. 2. Traditional law is formed by unwritten customs" [David et al. 1995, p. M-38]. Since case law is not even mentioned among the sources of law listed in the David et al.'s (1995) report, *Statute-I* = *Statute* = 1.

2.1.48 Malta

"In 1798, the islands were conquered by Napoleon who ruled them for two years. The short period of the French rule is recognized as a beginning of the Maltese modern legal history, adopting the Napoleon concept of codification. When Malta shifted to the control of Great Britain in 1814, it kept its own legal system and traditions. Despite British rule

lasting more than a century (until 1949), Malta's legal system has remained based on the mixture of continental civil law and common law. The Maltese civil law was influenced particularly by the Roman law and Napoleonic codes. The Maltese Civil Code of 1870 was a combination of the Code Napoléon of 1804, the Italian civil code of 1865, and the Roman Catholic canon law. Since then, the code has been gradually revised until its latest version enacted in 2000. The commercial law also followed the French legal model" [Acartürk et al. 2005, p. 494]. As a consequence, *Statute-I* = 1; *Comprehensive-Appeal-I* = 1; *Judgment-Law-I* = 1; *Inquisitorial-I* = 1; *Written-Evidence-I* = 1. "Modern Maltese law is derived from the following sources: (1) Statute law [...]; (2) Commercial custom [...]; (3) Judicial decisions [...]. A judicial decision, even by the highest court in the land, does not constitute a binding precedent" [David et al. 1995, p. M-47]. Therefore, *Statute* = 1.

2.1.49 Mauritania

Mauritania was a French colony from the late 1800s until 1960 (Britannica, 2015). There are two sources of law: "1. Modern law consists of a wide variety of texts which are either a colonial inheritance or the work of the new order. Under Const. art. 60, texts of the colonial era are applicable until such time as they are replaced by new ones [...]. 2. Islamic law is traditionally applicable to nearly all the population in respect of most aspect of private law" [David et al. 1995, p. M-55]. Since judicial decisions are not even mentioned among the sources of law listed in the David et al.'s (1995) report, *Statute-I* = *Statute* = 1.

2.1.50 Mauritius

"First settled by the French in 1715, Mauritius was conquered by the British in the Napoleonic Wars and was formally ceded to Great Britain by the Treaty of Paris, 1814. On 12 March 1968 Mauritius became a sovereign democratic state within the British Commonwealth of Nations. It is a constitutional monarchy of which the monarch is Elizabeth II, Queen of Mauritius and of Her Other Realms and Territories, Head of the Commonwealth. [...] The basic laws are contained in the French Code Napoléon, Code de Procédure Civile, Code de Commerce and Code Pénal as in force at the time of the conquest by the British" [David et al. 1995, p. M-59 and M-60]. As a result, *Statute-I* = *Statute-I* = 1.

2.1.51 Mexico

Mexico "acquired its independence from Spain in 1810 [. . .]. Mexico's legislation follows closely [French law] via the Spanish heritage [. . .]. The legal system has its roots in Spanish colonial legislation as well as pre-Colombian indigenous law. After independence Mexican legal codification took place in the second half of the 19th century, and bore the mark of French, Spanish and Italian legal doctrines of this period. The Mexican revolution of 1910-1920 brought about profound changes in the organization of government, the judicial, and legal systems. The 1917 constitution is still valid today, with a number of amendments. The civil code for the federal district [. . .] was adopted after the revolution, in 1928, and serves as a model for the state civil codes. The Mexican states have their own civil codes, most of which are copies of or follow closely the federal district civil code. The latter was reformed in 1987. The commercial code of Mexico governs commercial activity throughout the country [. . .]. The current commercial code was adopted in 1889 and is federal law. Prior commercial codifications were completed in 1854 and 1884, based on Spanish commercial legislation of the 18th century [. . .]. The Mexican laws of civil procedure also follow the French legal model through its Spanish influence [. . .]. The current code of civil procedure was adopted in 1932" [Acartürk et al. 2005, p. 501]. Therefore, *Statute-I* = 1; *Comprehensive-Appeal-I* = 1; *Judgment-Law-I* = 1; *Inquisitorial-I* = 1; *Written-Evidence-I* = 1. "An institution known as "compulsory case law" has been established in Mexican law. Under this rule, the decisions pronounced by certain courts become compulsory to courts of the same standing and to lower courts. The opinion of the Supreme Court of Justice and of the Collegiate Circuit Courts on all matters within their competence are considered in some cases compulsory for the same and lower courts, and not only for the federal but also for the state courts [. . .]. In the same cases, the decisions of the following courts also become compulsory case law: (I) Plenary session of the Federal Fiscal Court [. . .] and (2) Divisions of the Contentious-Administrative Court" [David et al. 1995, p. M-68]. As a result, *Statute* = 0.

2.1.52 Monaco

"The House of Grimaldi established the Principality of Monaco in 1297. It acquired its independence from France in 1419 and since then it has been a sovereign independent state

except for the period from 1789 to 1814 when it was controlled again by the French. The Civil Code that was originally passed in 1818 was [. . .] revised by three decrees from 1880 to 1884 [. . .]. The civil procedure and practice of courts are quite similar to the French practice, as they were modeled on the French code of civil procedure" [Acartürk et al. 2005, p. 513]. Thus, *Statute-I* = 1; *Comprehensive-Appeal-I* = 1; *Judgment-Law-I* = 1; *Inquisitorial-I* = 1; *Written-Evidence-I* = 1. Since judicial decisions are not even mentioned among the sources of law in the David et al.'s (1995) report [see David et al. 1995, p. M-81], *Statute* = 1.

2.1.53 Morocco

Morocco "acquired its independence from France in 1956 [. . .]. [Its legal system] was influenced by Spanish and French civil law during the protectorate regimes prior to its independence. In 1913, the French rule promulgated a range of civil and commercial codifications. In the same year, Morocco adopted a Code of obligations and contracts which, in fact, repeated sections of the French Civil Code, but modified to accommodate local conditions. The current Moroccan legislation is considered more liberal in development compared to the legal trends in other Islamic countries of North Africa. The Shari'a, is limited to family law related questions while general commercial law remains closely tied to French [law]. The present Civil Code, as amended from time to time covers mostly contractual obligations and regulates relationships between the parties in most transactions except for commercial ones. The New Commercial Code developed on the basis of the French system and model of codification and entirely replaced the previous code of 1913. The Code which came into force in 1996 provides the legal framework for contracts, obligations, and insolvency proceedings. The first civil procedure rules were adopted in Morocco in 1913 as a separate codification during French rule. They were further modified in 1974 with a new Code of Civil Procedure [. . .]. The most recent code modification occurred in 1993" [Acartürk et al. 2005, p. 521]. So, *Statute-I* = 1; *Comprehensive-Appeal-I* = 1; *Judgment-Law-I* = 1; *Written-Evidence-I* = 1; *Inquisitorial-I* = 1. Case law is not even mentioned among the sources of law listed in the David et al.'s (1995) report [see David et al. 1995, p. M-96]. So, *Statute* = 1.

2.1.54 Netherlands

"The Netherlands' civil law history dates back to the Roman Empire, when classical

Roman law gave way to growing customary law. In the 15th century, Romanic law was restored in some European countries, including the Netherlands, though customary law was also a source of law. During the 16th and 17th centuries, the Dutch developed a new legal tradition based solely with Roman law. This initiative brought into existence a new legal system recognized as Roman-Dutch law. However, Napoleon's dominance imposed French codes on the Dutch provinces" [Acartürk et al. 2005, p. 554]. After independence (1815), "in 1838, the Netherlands revised its inherited legislation and adopted a new code of judicial organization, civil code, civil procedure code, and commercial code. The codes still had a strong French influence [...]. The need for modern civil legislation resulted in the drafting of a new Dutch civil code in the 1930s. The new code reorganized and encompassed civil and commercial law into a single code. This code is still being amended [...]. The civil procedure and practice of courts legislation also remain very close to the French civil practice and model. The original version of the Dutch Code of Civil Procedure of 1838 has been a subject of gradual revisions and amendments. The current Dutch procedure code was substantially modified in 1991 and its last changes came into force in 1997" [Acartürk et al. 2005, p. 554]. Therefore, *Statute-I* = 1; *Comprehensive-Appeal-I* = 1; *Judgment-Law-I* = 1; *Inquisitorial-I* = 1; *Written-Evidence-I* = 1. "Court decisions cannot legally be considered as a source of law" [David et al. 1995, p. N-14]. As a consequence, *Statute* = 1.

2.1.55 Nicaragua

Nicaragua was a Spanish colony from 1529 to 1838 (Britannica, 2015). "The Civil Code, in force since 1904, is more liberal than the previous 1867 Civil Code which was practically a copy of the Chilean Code" [David et al. 1995, p. N-40]. "Supreme Court decisions are published yearly in the Boletin Judicial. These decisions are not considered a binding precedent" [David et al. 1995, p. N-40]. As a result, *Statute-I* = *Statute* = 1.

2.1.56 Niger

Niger was a French colony from 1922 to 1956 (Britannica, 2015). Sources of law are "modern law and customary law. 1. [...] In modern law the Republic of Niger has inherited numerous legislative provisions which date from the colonial regime, and these have been kept in force by art. 76 of the Constitution. 2. [...] Customary law is drawn largely from

Koranic precepts" [David et al. 1995, p. N-47 and N-48]. Since case law is not mentioned among the sources of law listed in the David et al.'s (1995) report, *Statute-I* = *Statute* = 1.

2.1.57 Panama

Panama "acquired its independence from Colombia in 1903, while independence from Spain was gained in 1821 [...]. Panama's civil code was adopted in 1916 and was based on the Spanish civil code of 1889. The commercial code of 1916 as well followed closely the Spanish model, but also incorporated elements from other commercial codifications. Since then, the civil and commercial codes have been amended many times, but retain their original nature. The Panamanian laws of civil procedure also follow [French law] through Spanish influence; however the procedure (civil, criminal, and commercial) is treated as one concept [...]. The first procedural code was adopted in 1917 and amended frequently afterwards. The current procedural code was adopted in 1984 and amended in 1986, 1991, and 2001" [see Acartürk et al. 2005, p. 598]. Therefore, *Statute-I* = 1; *Comprehensive-Appeal-I* = 1; *Judgment-Law-I* = 1; *Inquisitorial-I* = 1; *Written-Evidence-I* = 1. "[C]ase law is not recognized as a source of law" [David et al. 1995, p. P-20]. As a consequence, *Statute* = 1.

2.1.58 Paraguay

Paraguay "acquired its independence from Spain in 1811 and is currently constituted as a unitary republic [...]. The majority of legislation was taken from Argentina without much attempt to adapt it to local conditions or build up new codes. The various colonial Spanish laws governed civil law in existence around 1810, and Paraguayan civil code of 1876 was essentially a copy of the Argentine civil code of 1869. This code remained effective with various amendments until 1987, when a new civil code entered into force. The new code follows the tradition of the French civil and commercial codes and also utilizes Italian, Brazilian, and Swiss sources. The civil procedure was also governed by Argentine sources, and in 1883 Paraguay adopted the Argentine code of civil procedure of 1880. Thus, civil procedure rules also follow [French law] via Spanish influence. A new code of civil procedure entered into force in 1988. Paraguay's judicial system also reflects the impact of Roman law and the Spanish and French traditions, particularly the Napoleonic Code" [Acartürk et al. 2005, p. 606]. Therefore, *Statute-I* = 1; *Comprehensive-Appeal-I* = 1; *Judgment-Law-I* =

1; *Inquisitorial-I* = 1; *Written-Evidence-I* = 1. "Judicial decisions are not recognized as a source of law. Even a decision of the Supreme Court which declares a law unconstitutional and therefore of no effect for the case before the Court, in not extended to the other cases (Const. art. 260 no. 1)" [David et al. 1995, p. P-44 and P-45]. As a result, *Statute* = 1.

2.1.59 Peru

Peru "acquired its independence from Spain in 1821 [...]. The development of Peruvian private law since independence from Spain has been strongly influenced by French law. The first Civil Code of 1852 and the second Civil Code of 1936 followed the Napoleon Code very closely. The new Civil Code, adopted in 1984, remains within the same legal tradition, although complemented by modern Italian and German elements. The 1853 and 1902 Commercial Codes closely followed the Spanish codes of 1829 and 1885, which were also based on the French model. A number of new laws have been enacted during the last three decades dealing with civil and commercial issues, such as companies, bankruptcy, commercial papers, and securities. The new legislation has substantially modernized the old codes, albeit maintaining the two-centuries-old civil legal tradition. Civil litigation in Peru since colonial times has followed closely [French law], through the influence of Spanish and later Italian institutions [...]. The 1992 code replaced the code of 1912, which replaced the Code of 1853, and draws heavily upon both. The 1993 constitution and the Organic Law of the Judiciary define the structure of the judicial system" [Acartürk et al. 2005, p. 613]. As a consequence, *Statute-I* = 1; *Comprehensive-Appeal-I* = 1; *Judgment-Law-I* = 1; *Written-Evidence-I* = 1; *Inquisitorial-I* = 1. "When the Constitutional Court declares all or part of a law unconstitutional, the judgment is published in the official journal [...]. From the day following the day of publication, the affected provisions lose their effect [...]. Resolutions of the Full Supreme Court which lay down judicial doctrines must be published in full in the official journal and are binding upon all Peruvian courts [...]. Other judicial decisions are not regarded as source of law [...], although they are recognized as significant in practice" [David et al. 1995, p. P-115]. Therefore, *Statute* = 0.

2.1.60 Philippines

The Philippines "acquired its independence from the United States in 1946 [...]. The

Philippines' legal system is primarily based on the United States model and Spain's codified legislation. During the second half of the 19th century, Spain transferred and imposed much of its codified laws. The Spanish enforced its Commercial Code in 1885 and the Civil Code in 1889. At the end of the 19th century, the Philippines was ceded by Spain to the US, and thus Anglo-American law was incorporated in the Philippine legal system. The American influence was mainly in public law [...]. In 1950, the Spanish civil code was substantially revised. The new Civil Code carried with it the civil law traditions, and it still governs property rights, obligations, and contracts matters. However, the commercial legislation, which was also modified, is now comprised of separate codes based on U.S. models. The first civil procedure rules were disseminated in the Philippines in 1853 through the Spanish Code of Civil Procedure" [Acartürk et al. 2005, p. 624]. So, *Statute-I* = 1; *Comprehensive-Appeal-I* = 1; *Judgment-Law-I* = 1; *Inquisitorial-I* = 1; *Written-Evidence-I* = 1. "[S]tare decisis has been incorporated into the legal system. CC art. 8 states: "Judicial decisions applying or interpreting the laws or the Constitution shall form part of the legal system of the Philippines." The decisions of the Supreme Court establish precedents which are binding on subordinate courts. Decisions of the Court of Appeals are not strictly binding upon inferior courts, but they [have] persuasive authority" [David et al. 1995, p. P-135]. So, *Statute* = 0.

2.1.61 Poland

Poland "acquired its independence from the Soviet Union in 1989 [...]. During the socialist period [previous] legislation was amended or replaced by a new body of laws along socialist legal principles. A new civil code was adopted in 1964, following the content and principles of the Soviet civil code, although in form it resembled the French civil code and incorporated certain unique provisions regarding property and land ownership and some types of obligations. The majority of socialist-inspired laws were passed in the 1960s. A special feature of the socialist period is that some of the pre-war legislation still provided the concepts or rules for certain legal relations. For example, sections of the 1934 commercial code were still applied. A new code of civil procedure based on socialist legal principles was passed in 1964, thus replacing the old pre-war civil procedure code from 1930. The civil procedure code from 1964 has been further amended in 1985 and 1996. The latter

amendment introduced major changes into Polish civil procedure and the judicial system" [Acartürk et al. 2005, p. 644]. Thus, *Statute-I* = 1; *Comprehensive-Appeal-I* = 1; *Judgment-Law-I* = 1; *Inquisitorial-I* = 0.5; *Written-Evidence-I* = 0. "Poland follows the Civil Law tradition according to which judicial decisions are not strictly binding as precedents and are thus not a formal source of law" [David et al. 1995, p. P-156]. Thus, *Statute* = 1.

2.1.62 Portugal

"Portugal's legal system has roots in Roman and canonic law, intervened with modern codified law and ancient customary law from several Portuguese regions. The most significant influences on Portugal private law are the French codes of the nineteenth century, the canonic law of the middle ages, and several German compilations [...]. The first Civil Code, dated 1867, remained active until 1966, when a new Code was approved. This code of 1966 is the one currently in force, though there have been subsequent alterations [...]. The first Commercial Code was published in 1833. In 1888, commercial law was codified following the French model" [Acartürk et al. 2005, p. 653]. The Civil Procedure Code first published in 1876 follows closely French law [see David et al. 1995, p. P-199 to P-200]. As a consequence, *Statute-I* = 1; *Comprehensive-Appeal-I* = 1; *Judgment-Law-I* = 1; *Inquisitorial-I* = 1; *Written-Evidence-I* = 1. "Judicial jurisprudence does not have binding force, but [...] [t]he ruling issued by the Supreme Court of Justice to resolve "conflicts of jurisprudence" (CC art. 2) are regarded as a formal source of law. [Yet] [s]hould the Constitutional Court regard this practice as unconstitutional, as appears likely, conflicts of jurisprudence may have to be dealt with in another way" [David et al. 1995, p. P-189]. Given how remote are the circumstances under which the Supreme Court's rulings become binding, *Statute* =1.

2.1.63 Rwanda

Rwanda "became a German protectorate in 1899. In 1919 it was placed under Belgian administration by the League of Nation; and in 1946 was subjected to the international trusteeship system set up by the United Nations charter [...]. On 1 July 1962 it became a sovereign independent state [...]. The principal sources of law are custom and statute [...]. [Among the latter, there] is the colonial legislation (above all the adaptations of the Napoleonic Code) still in force in so far as is consistent with the Constitution [...]. Since

the independence of Rwanda a large number of statutes have been drawn up [...]. [T]he judicial system of Rwanda does not accord a very important place to case law" [David et al. 1995, p. R-39 to R-41]. As a consequence, *Statute-I* = *Statute* = 1.

2.1.64 Senegal

"As a former French colony, Senegal obtained its legal traditions through the Civil Code of 1830 and the Commercial Code of 1850. Prior to their independence [1960], French civil law was more widely applied in Senegal than in the other colonies within French West Africa and was the main source of law. However, customary law continued governing cases of personal matters. Since 1960, the established legal system has been transformed to accommodate the needs of the inhabitants, most of which are Muslim. The Civil Code was consecutively replaced as follows: first, by a code of obligations in 1963; second, by a set of property laws in 1964; and finally, by a family code in 1972. The family code, however, which was drafted according to the Shari'a, also follows [French law]. The code of obligations replaced of the Commercial Code of 1850 and was modified with amendments until 1985, when its current version was enacted. Senegal's Code of Civil Procedure was adopted in 1964 and entirely replaced the previous code, established during the French rule. Procedural law was further amended several times from 1978 until 2001 and it remains true to the French civil practice" [Acartürk et al. 2005, p. 677]. Thus, *Statute-I* = 1; *Comprehensive-Appeal-I* = 1; *Judgment-Law-I* = 1; *Inquisitorial-I* = 1; *Written-Evidence-I* = 1. "The sources of modern law are the same as those in France" [David et al. 1995, p. S-28]. Thus, *Statute* = 1.

2.1.65 Seychelles

"The first French settlers of Seychelles introduced the French Code civil [...] and the Code de commerce [...]. These Codes continued to apply after 1814 when Great Britain took over the colonial government of the islands. However, a parallel legislation in English and in the style of the common Law grew up progressively to such an extent that there was considerable confusion and uncertainty in the law, while imposing a heavy burden upon its administration. This acted as a fetter to development and progress. In anticipation of independence, a throughout revision of civil law took place between 1972 and 1976 [year of independence]" [David et al. 1995, p. S-43]. Thus, *Statute-I* = 1. "Judicial decisions are

not binding upon other courts, but they enjoy high persuasive authority from which a court may only depart for good reason" [David et al. 1995, p. S-43]. Thus, *Statute* = 1.

2.1.66 Slovenia

Slovenia "acquired its independence from the Yugoslav Federation in 1991. Historically, Slovenia was part of the Austrian Empire from the 14th century until 1918, and became part of the Yugoslav kingdom in the interwar period [...]. Slovenia [adhered] to the principles of socialist legality as part of the Socialist Federative Republic of Yugoslavia from 1945 until 1990 [...]. Once the process of disintegration of Yugoslavia was under way during the late 1980s Slovenia was the first of the constituent republics to break away. Since 1991 a new constitution has been adopted and the current law is a mixture of Slovenian Republic laws, Austrian laws, and Yugoslav federal laws. The Austrian civil code is still applied to certain issues, although a new Code of Obligations was drafted in the mid-1990s and entered into force in 2001. At present there is no commercial code as the Code of Obligations covers the respective legal issues; however a new company law (1993) has been adopted. The 1999 Law on Civil Procedure replaced the code of civil procedure of Yugoslavia from 1977" [Acartürk et al. 2005, p. 700]. As a consequence, *Statute-I* = 1; *Comprehensive-Appeal-I* = 1; *Judgment-Law-I* = 1; *Inquisitorial-I* = 0.5; *Written-Evidence-I* = 0. *Statute* = 1.

2.1.67 Somalia

"During the colonial and post-colonial period [1889-1960], the European powers (Italy and Great Britain) as well as the Italian Trust Administration introduced in the North and in the South of Somalia various legal patterns modelled on civil and common law [...]. After independence and unification the Somali government was faced with a very heavy legislation program to unify the legal system in the North with that in the South" [David et al. 1995, p. S-70]. "Somalia abolished all traditional courts and has completely replaced them with those based on the Italian model" [David et al. 1995, p. S-68]. So, *Statute-I* = *Statute* = 1.

2.1.68 Spain

"The Spanish legal system is rooted in Roman and canon law and is also intertwined with modern codified law and the ancient customary law of various Spanish regions. In 1829 the commercial law was codified following the French model and significantly modernized in

1885. The Civil Code, enacted in 1889, continues to form the core of Spanish private law. In the area of contracts and obligations the code closely follows the letter of the Code Napoléon, while family law mostly derives from canon law. The ancient Spanish fueros (laws) of the different localities, and the thirteenth century Siete Partidas, heavily influenced by Roman law, have left an imprint on current Spanish civil litigation. Until 2001, the 1881 Code of Civil Procedure, which replaced that of 1855, constituted the main body of law on civil proceedings in Spain. The closest precedent to the 1881 Code was the codification movement that was initiated in France in 1804 and followed by the Constitution of Cadiz of 1812" [Acartürk et al. 2005, p. 721]. Thus, *Statute-I* = 1; *Comprehensive-Appeal-I* = 1; *Judgment-Law-I* = 1; *Written-Evidence-I* = 1; *Inquisitorial-I* = 1. "The civil Code establishes the legal sources of Spanish law—apart from the Constitution [...]—in the following order: Laws (statutes); customs; and the general principles of law [...]. [T]he courts have as their only function [...] that of applying the legal norms, not to create them" [David et al. 1995, p. S-98]. Thus, *Statute* = 1. The Spanish colonies in the sample gained their independence between 1810 and 1968, before the 2001 New Code of civil Procedure was issued. Hence, the transplanted adjudication institutions are those described in the 1806 French Code of Civil Procedure.

2.1.69 St. Lucia

"The basis of the civil law is mainly that of pre-revolutionary France" [David et al. 1995, p. S-7], i.e., Roman-canonic law based on centralized legislation [see Acartürk et al. 2005, p. 264]. Indeed, "the Civil code ordinance of 1876 introduced into Saint Lucia the civil law of Quebec as it then stood with slight modifications to suit local conditions" [David et al. 1995, p. S-7]. Judicial decisions are not even mentioned among the sources of law listed in the David et al.'s (1995) report [see David et al. 1995, p. S-7]. So, *Statute-I* = *Statute* = 1.

2.1.70 Swaziland

"The Kingdom of Swaziland acquired its independence from the United Kingdom in 1968 and since then has been a fully independent member of the British Commonwealth. [Despite British rule,] Swaziland's legal systems remain based on South African/Roman-Dutch legal principles" [Acartürk et al. 2005, p. 748]. To elaborate, by "proclamation of February 22, 1907, the Roman-Dutch common law, save in so far as the same has been modified by statute,

is law in Swaziland" [Lee 1915, p. 12]. Given the analysis of the Netherlands above, this implies *Statute-I* = 1; *Comprehensive-Appeal-I* = 1; *Judgment-Law-I* = 1; *Inquisitorial-I* = 1; *Written-Evidence-I* = 1. Case law is not even mentioned among the sources of law in the David et al.'s (1995) report [see David et al. 1995, p. S-154]. As a result, *Statute* = 1.

2.1.71 Syria

In the aftermath of the independence from France (1944), "in 1949 the new Civil Code was drawn up. It was based on the French, Swiss and German Codes and on the Franco-Italian draft Code of Obligations. It was a close copy of the Egyptian Civil Code which had recently appeared" [David et al. 1995, p. S-208]. Judicial decisions are not even mentioned among the sources of law listed in the David et al.'s (1995) report [see David et al. 1995, p. S-207 and S-208]. As a consequence, *Statute-I* = *Statute* = 1.

2.1.72 Togo

"The national sources of law relate partly to the modern law and partly to the traditional law. In modern law Togo has inherited the legislation dating from the previous [French] regime [ended in 1960]" [David et al. 1995, p. T-20 and T-21]. So, *Statute-I* = *Statute* = 1.

2.1.73 Tunisia

"Tunisia became an autonomous province of the Ottoman Empire in 1574. Then, the country became a French Protectorate in 1881 [and] gained its independence in 1956 [...]. The Civil Code consists of a code of personal status, a code of real property law, and a code of obligations and contracts, all of which are based on French Law, with the exception of the code of personal status which was inspired by Islamic law [...]. The Commercial Code adopted in 1959 is a combination of French civil and commercial law [...]. [C]ommercial law also includes the Code of Obligations and Contracts of 1906. The civil procedure law and practice of the courts originate from the French [Law]. The Code of Civil and Commercial Procedure was adopted in 1959" [Acartürk et al. 2005, p. 807]. So, *Statute-I* = 1; *Comprehensive-Appeal-I* = 1; *Judgment-Law-I* = 1; *Inquisitorial-I* = 1; *Written-Evidence-I* = 1. "A judge may recognize no other authority than that of the law. In one case, however, he must accept the interpretation of the law which is given by another court: an interpretation given in judgment of the full court [...] de Cassation is binding upon the court to

which a case is sent back after second appeal. In fact, however, courts rarely depart from the interpretation of the law given by higher courts, and in particular by the Cour de Cassation. This court, which has the task of ensuring the uniform interpretation of the law, both controls and plays the major part in developing case law. It should be added that the courts, and particular the Cour de Cassation, must in the course of their task of interpreting the law also fill in any lacunas in it. This means that a considerable number of rules of substantive law have been created by case law" [David et al. 1995, p. T-35]. So, *Statute* = 0.

2.1.74 Ukraine

Ukraine "acquired its independence from the Soviet Union in 1991, having been a constituent Soviet republic since 1921 [. . .]. Since independence Ukraine has adopted a substantial body of new legislation, a lot of which is still under review [. . .]. A new constitution was adopted in 1996. [A] new civil code [has been promulgated] in late 2001. The code of civil procedure of the Ukrainian Socialist Republic from 1963 is still valid with appropriate amendments" [Acartürk et al. 2005, p. 859]. Thus, *Statute-I* = 1; *Comprehensive-Appeal-I* = 1; *Judgment-Law-I* = 1; *Inquisitorial-I* = 0.5; *Written-Evidence-I* = 0; *Statute* = 1.

2.1.75 Uruguay

Uruguay "acquired its independence from Spain in 1825 [. . .]. After independence, a new constitution was adopted in 1830, but most of the Spanish colonial legislation remained in force for several decades thereafter. Commercial law was codified first in Uruguay in 1866 by adopting the commercial code of the Province of Buenos Aires from 1858, with appropriate revisions. The Argentine code itself followed both the French commercial code of 1807 and the Spanish code from 1829. The commercial code was substantially reformed, introducing Italian and German elements, in 1900 and 1989. The civil code of Uruguay was adopted in 1868 and also closely followed the French Code of Napoleon from 1804. It was developed in a systematic manner in the 1860s and combined elements from relevant civil codification drafts in Argentina, Brazil and Spain. It was revised along modern civil law principles in 1893, 1914 and 1994. Civil litigation in Uruguay has also followed closely [French law], through the influence of Spanish and later Italian institutions [. . .]. The current code came into force in 1989. The 1967 constitution, with its amendments from 1996, and the Organic

Law on Judicial Power and Organization of Courts [...], further amended in 1987, define the structure of the judicial system" [Acartürk et al. 2005, p. 866]. Thus, *Statute-I* = 1; *Comprehensive-Appeal-I* = 1; *Judgment-Law-I* = 1; *Inquisitorial-I* = 1; *Written-Evidence-I* = 1. "Case law is not a source of law" [David et al. 1995, p. U-175]. Hence, *Statute* = 1.

2.1.76 Vatican

The Vatican was part of Italy between 1870 and 1929 (David et al., 1995). "The principal sources are (1) the Codex Juris Canonici and apostolic constitutions; (2) Laws issued for the Vatican by the Pontiff or authorities empowered by him (art. 1). Supplementary sources, in matters on which the principal sources do not make provision, are the Italian civil, commercial and penal law in force, together with the general regulations of the province and the municipality of Rome" [David et al. 1995, p. V-2]. So, *Statute-I* = *Statute* = 1.

2.1.77 Venezuela

Venezuela "acquired its independence from Spain in 1811 [and] was made part of the vice-royalty of New Granada [...] until independence in 1811. It became part of the new confederate Republic of Gran Colombia, comprising also Ecuador and Colombia. In 1830 this federation ceased to exist, and three separate states were created: Venezuela, New Granada and Ecuador. Venezuela adopted its first constitution in 1830. During the colonial period, Spanish legislation was in force and remained valid until 1863. In 1862 the first civil code was adopted, which was inspired by the Chilean civil code of 1855, itself the work of the Venezuelan jurist Andres Bello [...]. In 1867 a new civil code came into force, based on Napoleon's Code and the Spanish Code enacted in the same year. In 1873 another civil code was adopted, following [French law] but also encompassing modern elements from the Italian code at that time. The current code was adopted in 1942, was modified in 1982, and follows the same tradition. Commercial legislation also follows the French codes. The first commercial code was passed in 1862 and was replaced by a new code in 1873. The latter was modeled on the French commercial code, but also incorporated elements from Spanish, Italian, German and English sources. A new commercial code came into existence in 1909, was reformed in 1919, and amended last in 1955. The Venezuelan laws of civil procedure also follow [French law] through the Spanish influence [...]. Procedural codification preceded

civil law codification. The first code of judicial procedure was adopted in 1836 and included civil, commercial and criminal procedure. A new separate civil procedure code was drafted in 1863 and came into force after the civil war, in 1873. The current code of civil procedure was adopted in 1987" [Acartürk et al. 2005, p. 881]. Thus, *Statute-I* = 1; *Comprehensive-Appeal-I* = 1; *Judgment-Law-I* = 1; *Inquisitorial-I* = 1; *Written-Evidence-I* = 1. "Court decisions are not a source of law" [David et al. 1995, p. V-9]. Thus, *Statute* = 1.

2.1.78 Vietnam

"French colonial rule [on Vietnam] continued until 1954, when communist forces took control of North Vietnam. After the end of the war with the U.S. in 1973, the Democratic Republic of Vietnam (North) assumed control of the former Republic of Vietnam (South) in 1975, and the Socialist Republic of Vietnam was formed in 1976 [...]. Having been an independent vassal state of China since the 10th century, the kingdom of Nam-Viet had come to control most of the territory of Indo-China by the 18th century. The region fell under French influence in mid-19th century and was administered as three separate regions with a varying degree of autonomy. The Vietnamese legal system, which was in place before colonization, was influenced by medieval Chinese legal doctrine and local tradition, and the Code of Dynasty was one of the sources of customary law in the areas of property, family and inheritance from the 15th to the late 18th centuries. After the French colonized Indo-China, French codifications influenced the local legal system. Until 1975 French law was still largely valid in the South. After 1975 old laws were abrogated and new, socialist legislation was put in place. While the 19th century civil codes were influenced by the French civil code, a new civil code was adopted in 1996. It is a comprehensive body of laws, excluding only family law. A separate commercial code was never enacted despite a draft in the 1960s, but in 1997 a commercial law [...] was adopted. A code on civil procedure based on the French code of civil procedure was drafted in 1967, but never came into force. A decree governing civil procedure from 1989 serves as a guide for procedure" [Acartürk et al. 2005, p. 893]. Thus, *Statute-I* = 1; *Comprehensive-Appeal-I* = 1; *Judgment-Law-I* = 1; *Inquisitorial-I* = 1; *Written-Evidence-I* = 1. Case law is not mentioned among the sources of law in the David et al.'s (1995) report [David et al. 1995, p. D-15 to D-21]. Thus, *Statute* = 1.

2.1.79 Zaire

Belgium gained control over Zaire at the Conference of Berlin in 1885 and accepted its independence in 1960 (Britannica, 2015). "Taking into account the particular problems posed by textual interpretation, and the incompleteness of the texts in various subject matters, the directives contained in the Ordinance of 14 May 1886, and the necessary adaption to local situations, case law has played a decisive role: the judges have often had to look for original solutions which, even in cases where they have not afterwards been embodied in statute law, have often become traditional" [David et al. 1995, p. Z-4]. So, *Statute-I* = 1; *Statute* = 0.

2.2 Transplants to which Case Law was Transplanted

2.2.1 Anguilla

"Anguilla became a separate British Crown Colony in 1980 [...]. There was no legal system in existence when the British first settled the islands in the 17th century, and English law was introduced. The current law is the English common law and legal principles of 1623-1624, as amended by subsequent local or U.K. legislation. The current constitution was adopted in 1982 and was amended in 1990. Private law has been substantially developed by case law, and unified through appeals to the Judicial Committee of the Privy Council in London. Anguilla's civil procedure and practice of the courts follow very closely the English civil procedure and practice. Common law applies, unless superseded by a statute" [Acartürk et al. 2005, p. 1]. I consider Anguilla and the remaining territories under direct control of the UK as recipient of the UK adjudication institutions prevailing in the year of colonization. As a consequence, *Statute-I* = 0; *Comprehensive-Appeal-I* = 0; *Judgment-Law-I* = 0; *Inquisitorial-I* = 0; *Written-Evidence-I* = 0; *Statute* = 0.

2.2.2 Antigua

Antigua is a protected state, colonized by English settlers in 1632 and then administered by England until the 1981 independence (David et al., 1995). The law "consists of the English law in force in 1632" [David et al. 1995, p. U-115]. So, *Statute-I* = 0; *Statute* = 0.

2.2.3 Australia

Australia gained independence from the United Kingdom in 1900 (Britannica, 2015). "The English law was first officially incorporated in Australia in 1828, and the constitution was adopted in 1900. Private law has been substantially developed by case law and unified through appeals to the Judicial Committee of the Privy Council in England. The decisions of the House of Lords enjoyed precedential value within Australia until 1963. Appeals to the Privy Council were abolished in 1986. During the last two decades legislation has emerged as a prime source of law. Australia's civil procedure and practice of the courts also follow very closely the English civil procedure and practice, both at the federal and state levels [...]. The common law also applies, unless overridden by statute" [Acartürk et al. 2005, p. 17]. I

consider Australia and the remaining ex-British colonies as recipient of the UK adjudication institutions prevailing at independence. So, *Statute-I* = 0; *Comprehensive-Appeal-I* = 0; *Judgment-Law-I* = 0; *Inquisitorial-I* = 0; *Written-Evidence-I* = 0. "The general theory of the sources of law in Australia is derived from and resembles that applicable in the United Kingdom. The two main sources, in order of authority, are statutes of the parliament, and the (unenacted) Common Law" [David et al. 1995, p. A-55]. So, *Statute* = 0.

2.2.4 Bahrain

Bahrain "acquired its independence from the United Kingdom in 1971 [...]. In 1892, following several treaties between the Sheikh of Bahrain and the United Kingdom, Great Britain was recognized as a protecting power. [...] The English common law influence found its way in Bahrain through the application of the British legislation, The Bahrain Order in Council of 1913, issued in Buckingham Palace in 1913 under the authority of the British Government of India. In 1973, Bahrain adopted a Constitution, which declared Shari'a as a principle source of law. Although the preexisting laws were replaced by locally drafted legislation, they followed the format and style of Egyptian codes and laws which themselves originated from the French legal system. The first procedure rules were introduced in Bahrain through the Code of Civil and Commercial Procedure of 1971, which was influenced by French [law]" [Acartürk et al. 2005, p. 35]. Thus, *Statute-I* = 0; *Comprehensive-Appeal-I* = 0; *Judgment-Law-I* = 0; *Inquisitorial-I* = 0; *Written-Evidence-I* = 1. "In contrast to the pre-1970 legislation, the present trend reflects the influence of the Egyptian legal system, based on the Code Napoléon" [David et al. 1995, p. B-4]. Accordingly, case law is not even mentioned among the sources of law in the David et al.'s (1995) report. Thus, *Statute* = 1.

2.2.5 Bangladesh

"The first common law influences in the territory date back to the 18th century during the expansion of the administrative control and commercial contracts of the British Crown further than India. In the mid-19th century, the British dominion was extended and as a consequence Bengal became a region of India. The English common law was first introduced in Bengal through acts initially passed for India and adopted by Pakistan after its independence in 1947. The Constitution of Bangladesh owes its origin to the Proclamation of Indepen-

dence of April 10, 1971. The legislation in the private and commercial sphere is subject of revision through amendments of the existing law. The judiciary also follows the English common law concept of judicial precedent. The civil procedure and practice of the courts are similar to the English model. The Code of Civil Procedure of 1908, originally enacted in India, was adopted by Bangladesh after the independence and still utilizes the 1992 version" [Acartürk et al. 2005, p. 45]. Hence, *Statute-I* = *Statute* = 0; *Comprehensive-Appeal-I* = 0; *Judgment-Law-I* = 0; *Inquisitorial-I* = 0; *Written-Evidence-I* = 1.

2.2.6 Barbados

"Barbados was only settled after British sailors landed on it in 1627. For the next 300 years the island was a British colony, but enjoyed a large degree of local autonomy. The British established a type of representative government in 1661 alongside the local House of Assembly, a legislative organ that began its existence in 1639. From 1958 until 1962 Barbados was part of the West Indies Federation, and when the latter was dissolved it returned to its former status as a self-governing British colony. In 1966 Barbados became an independent country within the British Commonwealth [...]. Private law has been substantially developed by case law, and unified through appeals to the Judicial Committee of the Privy Council in London. Barbados still closely follows the English legal model both in drafting of new legislation and in its interpretation. The civil procedure and practice of the Barbadian courts also follow very closely the English civil procedure and practice" [Acartürk et al. 2005, p. 55]. As a result, *Statute-I* = *Statute* = 0; *Comprehensive-Appeal-I* = 0; *Judgment-Law-I* = 0; *Inquisitorial-I* = 0; *Written-Evidence-I* = 1 [see also David et al. 1995, p. B-8].

2.2.7 Belize

"Belize is a member of the British Commonwealth with the British monarch as the head of state. Belize acquired its independence from the United Kingdom in 1981 [...]. Belize's legal system is based on English common law and has remained very closely linked to the laws of the United Kingdom since colonial times. The British first settled Belize in 1638. Belize became a self-governing British colony in 1964. The English common law and all British statutes as of January 1, 1899 form the basic law of Belize. The current Belizean constitution was adopted in 1981 and amended in 1988. Belize's civil procedure and practice of the courts

also follow very closely the English civil procedure and practice. Common law also applies, unless overridden by statute" [Acartürk et al. 2005, p. 72]. As a consequence, *Statute-I* = 0; *Comprehensive-Appeal-I* = 0; *Judgment-Law-I* = 0; *Inquisitorial-I* = 0; *Written-Evidence-I* = 1; *Statute* = 0 [see also David et al. 1995, p. U-119 and U-120].

2.2.8 Bermuda

Bermuda is "a self-governing overseas territory of the [UK]. Current law is the English common law and statutes of 1612, as amended by subsequent local or U.K. legislation. Bermuda has developed its own legislation in finance, insurance, and corporate law. Private law has been substantially developed by case law and unified through appeals to the Judicial Committee of the Privy Council in London. Bermuda's civil procedure and practice of the courts also follow very closely the English civil procedure and practice. Common law applies, unless overridden by statute" [Acartürk et al. 2005, p. 77]. As a consequence, *Statute-I* = 0; *Comprehensive-Appeal-I* = 0; *Judgment-Law-I* = 0; *Inquisitorial-I* = 0; *Written-Evidence-I* = 0. Furthermore, *Statute* = 0 [see also David et al. 1995, p. U-119].

2.2.9 Botswana

Botswana "acquired its independence from the United Kingdom in 1966 and is a fully independent member of the British Commonwealth [...]. Botswana's legal system is a mixture of Roman-Dutch and English common law, and also incorporates features of customary tribal law. Roman-Dutch law was introduced in 1891 from the Colony of the Cape of Good Hope. The first statutory enactment to incorporate Roman-Dutch law into Botswana's law was the Proclamation of 1909, which established that the common and statutory law of the Colony of the Cape of Good Hope as of 1891 became the law of [Botswana]. The application of the Roman-Dutch law was initially intended only for European settlers, customary law applied to the local tribes. Thus, the legal system that developed was mainly based on the principles of the Roman-Dutch law rather than the English common law. However, English common law affected the Roman-Dutch law as applied in the Union of South Africa, and although rules of evidence, procedural law in both civil and criminal matters were based on Roman-Dutch law, the English common law has still influenced its' development. Constitutional, administrative, and commercial law and practice has also evolved to bear the imprint

of some aspects of the English common law system. These features were carried through to Botswana, and therefore, while recognizing that the legal system is of Roman-Dutch and some aspects of English Law have been adopted into the Roman-Dutch Law family. As in the English common law system precedent of a higher court is binding on lower courts in the Roman-Dutch legal system. Since independence, Botswana has followed Roman-Dutch legal principles. In 1973, the right of appeal before the Judicial Committee of the Privy Council in London was abolished [...]. The rules of procedure and evidence, the methods of proof and the treatment of precedents are within the Roman-Dutch [law]" [Acartürk et al. 2005, p. 92]. So, *Statute-I* = 0; *Comprehensive-Appeal-I* = 1; *Judgment-Law-I* = 1; *Inquisitorial-I* = 1; *Written-Evidence-I* = 1; *Statute* = 0 [see also David et al. 1995, p. B-39 and B-40].

2.2.10 British Virgin Island

"The British Virgin Islands are constituted as a common law overseas territory of the [UK]. There was no legal system in existence when the British first settled the islands in 1666, and English law was introduced. The current law is the English common law, and the legal principles of 1666, as amended by subsequent local or U.K. legislation. The doctrine of legal precedent governs the legal system, and decisions of a higher court are binding on lower courts. Thus, decisions made by the Privy Council are binding on the Court of Appeal and the Supreme Court, and decisions of the Court of Appeal are binding on the Supreme Court. The current constitution was adopted in 1977. The British Virgin Islands' civil procedure and practice of the courts also follow very closely the English civil procedure and practice. Common law applies, unless overridden by statute" [Acartürk et al. 2005, p. 117]. Hence, *Statute-I* = 0; *Comprehensive-Appeal-I* = 0; *Judgment-Law-I* = 0; *Inquisitorial-I* = 0; *Written-Evidence-I* = 0; *Statute* = 0 [see also David et al. 1995, p. U-118].

2.2.11 Brunei

Brunei was a British protectorate from 1888 to 1984, when it acquired its independence as constitutional monarchy (see Britannica, [2015]). "The basic law is the common law of England on 25 April 1951, [and] certain applied United Kingdom enactments" [David et al. 1995, p. U-113]. As a consequence, *Statute-I* = *Statute* = 0.

2.2.12 Burundi

"The lacunae in the law are, as in Zaire, filled by virtue of the [Order] of 14 May 1886 [imposed by the Belgium on Zaire while Burundi was under German rule] which requires the courts to devote themselves to the development of customary law, to apply general principles of law and to reach their decisions on the basis of equity. The role of case law has thus been [...] decisive" [David et al. 1995, p. B-93 and B-94]. Belgium occupied the country during the First World War leaving however untouched the existing settings that, therefore, survived even after the 1962 independence (see David et al., [1995]). Thus, *Statute-I* = *Statute* = 0.

2.2.13 Canada

"Canada is a constitutional monarchy and became a self-governing Dominion in 1867 while retaining ties to the British Crown [...]. The legal system of Canada and nine of its ten provinces, other than Quebec, is common law, having been strongly influenced in its development by the British common law" [Acartürk et al. 2005, p. 125]. Thus, *Statute-I* = 0; *Comprehensive-Appeal-I* = 0; *Judgment-Law-I* = 0; *Inquisitorial-I* = 0; *Written-Evidence-I* = 0. "In the Common Law provinces and at the federal level case law enjoys the same prominent position as a source of law as in other Common law jurisdictions, buttressed as it is by the principle of stare decisis" [David et al. 1995, p. C-18]. Thus, *Statute* = 0.

2.2.14 Cayman

"The Cayman Islands are constituted as an overseas territory of the [UK]. No legal system existed when the British first settled the islands in the early 18th century, and English law was introduced. Over the next 300 years, the islands were administered by Britain and from 1863 were a dependency of Jamaica. After Jamaica's independence in 1962, the islands opted to become a self-governing British Crown Colony. The current law is English common law, and the statutes and legal principles. The civil procedure and practice of the courts of the Cayman Islands are similar but not identical to the former English civil procedure and practice. Common law applies, unless overridden by statute" [Acartürk et al. 2005, p. 134]. Thus, *Statute-I* = 0; *Comprehensive-Appeal-I* = 0; *Judgment-Law-I* = 0; *Inquisitorial-I* = 0; *Written-Evidence-I* = 0. "The basic law consists of the laws in force in Jamaica on 22 June 1863" [David et al. 1995, p. U-118]. As a consequence, *Statute* = 0.

2.2.15 China

"Much of the Chinese legal system has been shaped by the debate between two opposing schools of thought: the Confucian and the Legalist. For most of China's history the Confucian legal philosophy has been dominant. However, influenced by the Europeanization at the end of the 19th and the beginning of the 20th centuries, Chinese legal system followed the civil law tradition of the Roman-Germanic legal systems" [Acartürk et al. 2005, p. 151] and, indeed, the 1929-1931 Civil code, the 1929 laws on company, and the 1935 civil procedure code were all shaped on "existing Japanese and German legislation" [David et al. 1995, p. P-68]. Therefore, *Statute-I* = 0; *Comprehensive-Appeal-I* = 1; *Judgment-Law-I* = 0; *Inquisitorial-I* = 1; *Written-Evidence-I* = 1. "Case law is of little importance. Precedents are not legally binding" [David et al. 1995, p. P-66]. As a consequence, *Statute* = 1.

2.2.16 Cyprus

Cyprus "acquired independence from the United Kingdom in 1960. In 1974 Cyprus was invaded by Turkey and one third of its territory was occupied by Turkey and is still under occupation [...]. During the period of British rule on Cyprus, common law was proclaimed in force with certain ethnic accords. The Greek population had its own family and domestic relations legislation based on the Greek law and the canon law of the Greek Orthodox Church. Meanwhile, the Turkish community was governed by the "Turkish family law" based on Islamic Shari'a. Since 1960, French and Greek legal doctrines have influenced the development of Cypriot public and administrative law. Even so, the Cypriot legal system continues to be affected by changes in the English common law. The current Commercial Code was enacted in 1959, which is based on Indian (colonial) legislation. The Code of Civil Procedure of 1968 follows the English [law]. The English doctrine of precedent and controlling jurisprudence is generally adopted in Cyprus. Usually, precedent is taken from decisions of Cypriot courts, but when Cypriot jurisprudence is silent, access is made to other United Kingdom and Commonwealth jurisdictions" [Acartürk et al. 2005, p. 191]. As a consequence, *Statute-I* = 0; *Comprehensive-Appeal-I* = 0; *Judgment-Law-I* = 0; *Inquisitorial-I* = 0; *Written-Evidence-I* = 1; *Statute* = 0 [see also David et al. 1995, p. C-105].

2.2.17 Dominica

"The Basic Law [...] consists of the common law and equity and statutes of England in force [in] 1763" [David et al. 1995, p. U-115]. Therefore, *Statute-I* = *Statute* = 0.

2.2.18 Ethiopia

"Up to the middle of the twentieth century [...] Ethiopian law was made up of the numerous customs by which the ethnic groups within the empire were ruled [...]. [T]he only source of law which ensured uniformity [...] was a book drawing its inspiration from Graeco-Roman and Middle Eastern sources [...]. With the reign of Menelik II which begins in 1889 [from this period until 1962, Ethiopia was subjected to political pressure from both Italy and England], we enter a phase of legislative activity [...]. Further developments occurred in the field of case law, as orders were then issued to keep records of cases decided in the imperial courts of appeal established all over the country [...]. After the interval of the Italian occupation, the process resumed in 1942. Since then the developments have been constant in all fields, culminating in the promulgation of the various codes of the 1950's and 1960's" [David et al. 1995, p. E-27]. Thus, *Statute-I* = 0. "Case law has no special role to play in the Ethiopian legal system; there are no rules as to value of precedent or the existence of stare decisis" [David et al. 1995, p. E-26]. Therefore, *Statute* = 1.

2.2.19 Falkland Islands

The UK took control of the islands in 1833 (Britannica, 2015). "The basic law consists of the common law, the rules of equity and the general statutes in force in England on 22 May 1900" [David et al. 1995, p. U-121]. As a consequence, *Statute-I* = 0; *Statute* = 0.

2.2.20 Fiji

"The Constitution is contained in the Fiji Independence [from England] Act, 1970 [...]. The basic law consists of the Common Law, the rules of equity and the statutes of general application which were in force in England at the date when Fiji obtained a local legislature [1875]" [David et al. 1995, p. F-30]. As a result, *Statute-I* = *Statute* = 0.

2.2.21 Finland

In "the mid-12th century [...] the Swedish conquered Finland and the Swedish codes

were entirely promulgated by the mid-14th century. Even though the Finnish provinces had a certain degree of autonomy, the Swedish law was mainly imposed on them. The General Code of 1734 was also adopted in Finland as in Sweden. After the Russian conquest in 1809, Finland became an autonomous grand duchy within the Russian empire. However, the Swedish law remained in force and it was greatly reformed in the 1860s and later on with respect to the private, family, commercial, and trade laws. The Finnish legal system stayed closely tied to the Swedish and other Nordic countries despite achieving independence in 1917. Legal reform that was accomplished in the 1950s came after the Swedish legal practice and examples. [...] There are no codifications governing the civil and commercial laws but a variety of laws are adopted to regulate these areas [...]. The civil procedure and practice of the courts are also inherited from the Swedish. A chapter of the General Swedish Code of 1734, concerning the rules for judges, is still in force" [Acartürk et al. 2005, p. 251]. Hence, *Statute-I* = 0, *Comprehensive-Appeal-I* = 0, *Judgment-Law-I* = 0, *Inquisitorial-I* = 1, *Written-Evidence-I* = 0."There have been a great number of statutory amendments within the framework of the Code of the 1734 [...;] however [...] there are wide gaps without any enactments, outside these areas covered by statutes and, even, within these areas, the statutes do not attempt to answer all the questions. In these circumstances the role of the court is very important [...]. In conclusion, it can be said that the principal sources of law consist of statutes as interpreted in the light of judicial practice, and to a more limited extent, customary law" [David et al. 1995, p. F-39]. Therefore, *Statute* = 0.

2.2.22 Gambia

"In the Gambia apply the Common Law, the doctrines of equity and the statutes of general application in force in England on 1 Nov. 1888 [...]. Written law is applied by the Supreme and Subordinate Courts, but they are not deprived of the right to enforce customary laws not incompatible with other laws and not repugnant to natural justice or good conscience" [David et al. 1995, p. G-10]. Thus, *Statute-I* = *Statute* = 0.

2.2.23 Ghana

Ghana "acquired its independence from the United Kingdom in 1957 [...Its] legal system is based on English common law, although it also incorporates elements of Islamic law

and customary law. The English common law and statutes of 1844 were adopted as the governing law upon colonization and were supplemented by local customary law [. . .]. Commercial legislation in Ghana follows the English [law] and has been recently modernized. The Ghanaian civil procedure was influenced by English law and follows closely the English civil procedure and practice. Ghanaian courts apply common law, statutory law, and customary law. Common law applies, unless overridden by statute [. . .]. Appeals to the Judicial Committee of the Privy Council in London were abolished in 1960" [Acartürk et al. 2005, p. 289]. So, *Statute-I* = 0; *Comprehensive-Appeal-I* = 0; *Judgment-Law-I* = 0; *Inquisitorial-I* = 0; *Written-Evidence-I* = 1. "The doctrine of judicial precedent [. . .] follows the British pattern. It is expressly provided that the Court of Appeal is bound in principle to follow its own previous decisions" [David et al. 1995, p. G-36]. So, *Statute* = 0.

2.2.24 Gibraltar

At the end of the War of the Spanish Succession (1701-1714), the British sovereignty over Gibraltar was recognized by the 1713 Treaty of Utrecht and thus England administered Gibraltar as a British Overseas territory until today (see Britannica, [2015]). "The basic law consists of the common law and the rules of equity from time to time in force in England and the enactments specified in the Application of English Law Ordinance" [David et al. 1995, p. U-110]. As a consequence, *Statute-I* = 0; *Comprehensive-Appeal-I* = 0; *Judgment-Law-I* = 0; *Inquisitorial-I* = 0; *Written-Evidence-I* = 0. Furthermore, *Statute* = 0.

2.2.25 Greece

"The Greek legal tradition has five major periods [. . .]: Greek period, up to the time when Greece became a Roman providence; a Hellenistic period, up to the foundation of the Byzantine empire; a Byzantine period, up to the fall of Constantinople; a post-Byzantine period, until the Greek revolution of 1821 [. . .]; and the modern period [afterwards]. Modern Greek civil law is contained in the Greek Civil Code of 1940 [. . .]. Prior to this code, the applicable civil law was Byzantine and Roman. After Greece's independence, the Civil law from the Byzantine period was introduced. In drafting a new civil law between 1821 and 1940, Greece also drafted its code based on Byzantine law. The commercial code is greatly influenced by French [law.] Civil Procedure is contained in the modern Code of Civil

Procedure that came in to force in 1968 [and] replaced the old Code of Civil procedure of 1834" [Acartürk et al. 2005, p. 298], which was mainly influenced by German law because drafted by Georg Ludwig von Maurer, a member of the Bavarian Regency in Greece [see David et al. 1995, p. G-49]. So, *Statute-I* = 0; *Comprehensive-Appeal-I* = 1; *Judgment-Law-I* = 0; *Inquisitorial-I* = 1; *Written-Evidence-I* = 1. "Greece has no rule regarding the binding effect of judicial precedents" [David et al. 1995, p. G-51]. So, *Statute* = 1.

2.2.26 Grenada

Grenada "acquired its independence from the United Kingdom in 1974, and is at present a member of the British Commonwealth [...]. Despite having been under French rule for a century, the civil law system was quickly dismantled, and the British managed to swiftly impose common law. From the late 18th century until Grenada's independence, the legal system and a body of laws developed closely in line with English common law. The current Grenadian constitution was adopted in 1975, and after some of its sections were repealed during the 1979-1983 period, it was restored in full in 1985 [...]. Grenada still closely follows the English legal model both in drafting of new legislation and in its interpretation, and currently its civil and commercial laws are undergoing reform and modernization. The civil procedure and practice of Grenada's courts follow very closely the English civil procedure and practice. Common law also applies, unless overridden by statute" [Acartürk et al. 2005, p. 307]. Thus, *Statute-I* = 0; *Comprehensive-Appeal-I* = 0; *Judgment-Law-I* = 0; *Inquisitorial-I* = 0; *Written-Evidence-I* = 1. "The Basic Law is probably the law of England in force in 1783. Local legislation follows largely analogous English laws in the fields of private and commercial law [...] and procedure" [David et al. 1995, p. G-65]. Hence, *Statute* = 0.

2.2.27 Guyana

"The Constitution is the supreme law [...] [l]egislative enactments, subordinate legislation and the decisions of the courts are also sources of the law. When the territory was ceded by the Dutch to the British in 1803, the common law then applicable was Roman-Dutch [...]. In 1917, the Civil Law of British Guiana Ordinance was enacted whereby Roman-Dutch law was [...] replaced by English law [...]. English statutes which had the force of law [at independence in 1966] continue to be part of the law of Guyana until abrogated by Act of

the Parliament" [David et al. 1995, p. G-90]. Thus, *Statute-I* = *Statute* = 0.

2.2.28 Hong Kong

"Hong Kong's legal system is based on the English common law and it was inherited during the British colonial rule. The British ended their administration in 1997 when Hong Kong was proclaimed a special administrative region of China. This was achieved through adoption of the Basic Law of 1990. The Basic Law serves as a mini-constitution assuring Hong Kong of its own government and legislature. It also provides for application of the pre-existing British laws, except for those that contravene the Basic Law and are subject to amendments. Thus, Hong Kong's legal system remains closely tied to the English common law and traditions. Chinese customary law, particularly probate law and land law, is considered as a second source of Hong Kong law and the courts may enforce the Chinese customary rights relating to land of the New Territories. [...] Hong Kong's civil laws, commercial laws and civil procedure rules, are regulated by a number of separate acts and ordinances. Common law applies, unless overridden by statute [...]. The judicial system in Hong Kong follows the common law tradition of binding precedent. [...] [T]he courts may refer to precedents of all common law jurisdictions" [Acartürk et al. 2005, p. 333]. Thus, *Statute-I* = *Statute* = 0; *Comprehensive-Appeal-I* = 0; *Judgment-Law-I* = 0; *Inquisitorial-I* = 0; *Written-Evidence-I* = 1 [see also David et al. 1995, p. U-113].

2.2.29 Iceland

"Iceland became a sovereign state under the Danish Crown in 1918 and acquired full independence in 1944 [...]. Norwegian and Celtic settlers in the late 9th and the early 10th centuries discovered Iceland. This marked the beginning of the Free Commonwealth period and the Icelandic legal system. Its main feature was the establishment of the General Assembly called the Althingi. The legislative body is still in force today. In the late 14th century, while under the Danish Crown, Iceland joined the Nordic union, including Denmark, Norway, Finland, Sweden, and Greenland [...]. Since then, Danish law has remained the major legislative source of the Icelandic legal system. After independence, Iceland developed its own legislation. Iceland has no codifications governing the civil and commercial laws. Instead, separate acts are adopted as regulation. In the 1990's, a series of acts were issued,

particularly in the area of company and competition law, capital movement and other. The Civil Procedure Law, adopted in December 1991, governs the civil procedure and practice of the courts" [Acartürk et al. 2005, p. 333]. Thus, *Statute-I* = 0, *Comprehensive-Appeal-I* = 1; *Judgment-Law-I* = 0; *Inquisitorial-I* = 0.5; *Written-Evidence-I* = 0. "Sources of law generally recognized in Icelandic legal theory are custom, precedent, the general principles of law and "the nature of the case." Icelandic courts have much the same attitudes towards these sources as Danish or Norwegian courts" [David et al. 1995, p. I-2]. Thus, *Statute* = 0.

2.2.30 India

"Prior to independence, India comprised British India, which was under the sovereignty of the United Kingdom, and Indian States, which were under British paramountcy. The independence Act [...] divested His Majesty's Government of responsibility for the government of British India as from 15 Aug. 1947, created two Dominions of India and Pakistan, made provision for a constituent assembly in each Dominion and provided for the lapse of British paramountcy over the Indian states. These states ultimately merged with India or Pakistan. The Constituent Assembly of India adopted a Constitutions which came into force on 26 Jan. 1950 [...]. The constitution continues in effect all the law in force in India immediately before its commencement until such law is altered, repealed or amended [...]. The main sources of law in India are legislation [...] and judicial precedents [...]. Judicial decisions in India have the force of precedents. Judgments of the Supreme Court are binding on all courts in India and judgments of the High Courts are binding on all subordinate courts in the State. A High Court is bound by its own judgment but not so the Supreme Court" [David et al. 1995, p. I-8 to I-11]. Thus, *Statute-I* = *Statute* = 0; *Comprehensive-Appeal-I* = 0; *Judgment-Law-I* = 0; *Inquisitorial-I* = 0; *Written-Evidence-I* = 1.

2.2.31 Ireland

"Prior to the 19th century, Irish law was mainly customary law despite the jurisdictional boundaries between the separate kingdoms. At that time, Irish law functioned together with English law. During the 19th century and earlier, the English Parliament at Westminster approved the entire English legislation for Ireland and all legal reforms performed thereafter were prolonged to the Irish legal system. In that way, English common law model was

generally introduced in Ireland. Since Ireland's independence [1921], the Irish Parliament (or Oireachtas) modernized the existing legal framework using the English model. Also the Irish legal system is greatly influenced by Brussels, since joining the European Union in 1973 and was originally influenced by early Brehon law and to a certain extent by Roman law [...]. Private law still follows the English common law [...]. [Since] the concept of precedent is followed, Ireland does not have major codifications. Civil and commercial laws are regulated by judicial interpretations [...]. Decisions of higher courts are binding on lower level courts. Ireland's civil procedure and practice of courts is generally similar to English civil procedure and practice. Common law applies, unless overridden by statute" [Acartürk et al. 2005, p. 364]. Thus, *Statute-I* = 0; *Comprehensive-Appeal-I* = 0; *Judgment-Law-I* = 0; *Inquisitorial-I* = 0; *Written-Evidence-I* = 0. "Law-making authorities are principally the people [...] and the courts" [David et al. 1995, p. I-66]. As a result, *Statute* = 0.

2.2.32 Israel

Israel "acquired its independence from the League of Nations mandate in 1948 [...]. From the 16th century until 1915, the area known as Palestine was part of the Ottoman Empire and was governed by Turkish authorities. During this period the law was a mixture of traditional Islamic and modern European laws, including German, French, and Swiss. A British Mandate was established in 1922 following a resolution by the League of Nations. Following the British Mandate, English principles of law were slowly brought into the region, replacing the existing common law. Since [independence in] 1948, Israel has adopted new legislation and completely transformed the previous legal system constituting the principal institutions of civil law. Although the common law system applies as a basis, the law has been influenced by the legal traditions of continental Europe and a number of laws influenced by the civil law have been enacted. Certain matters pertaining to family law (such as marriage and divorce), remain governed by Jewish law for Jews, by Islamic law (the Shari'a) for Muslims, and by Canon law for Catholics. Israeli procedural law tends to follow the British model of common law. The rules of civil procedure adopted in 1963 and amended in 1984 are closely based on British rules of 1938 established by the British Mandate" [Acartürk et al. 2005, p. 374]. Thus: *Statute-I* = 0; *Comprehensive-Appeal-I* = 0; *Judgment-Law-I*

= 0; *Inquisitorial-I* = 0; *Written-Evidence-I* = 1. "The doctrine of stare decisis came to the country as part of the English Common Law [...] but underwent certain modifications which received statutory expression in s. 33 of the Courts Law [...]. That section provides that every court shall be guided by a rule laid down by a higher court while a rule laid down by Supreme Court will bind every court except itself. Judgments always indicate the names of the judges by whom they are delivered; concurrent separate opinions are frequent; and so are dissenting opinions" [David et al. 1995, p. I-77]. As a consequence, *Statute* = 0.

2.2.33 Jamaica

Jamaica acquired its independence from the UK "in 1962 and is a member of the British Commonwealth [...]. [I]n 1728 the Crown agreed that English common law and all British statutes as of October 1, 1724 already in use on the island would form the basic law of Jamaica. The current Jamaican constitution was adopted in 1962 and amended in 1999. Private law has been substantially developed by case law and continues to be developed through local decisions and, ultimately, appeals to the Judicial Committee of the Privy Council in England. Jamaica still closely follows the English legal model both in drafting of new legislation and in its interpretation. Jamaica's civil procedure and practice of the courts also follow very closely the English civil procedure and practice. Common law also applies, unless overridden by statute" [Acartürk et al. 2005, p. 391]. "The 1962 Order in Council expressly preserves all laws which were in force in Jamaica immediately before independence" [David et al. 1995, p. J-1 and J-2]. Thus, *Statute-I* = *Statute* = 0; *Comprehensive-Appeal-I* = 0; *Judgment-Law-I* = 0; *Inquisitorial-I* = 0; *Written-Evidence-I* = 1.

2.2.34 Japan

"Japan legal history dates back as far as the 7th century when Buddhism and the Chinese law were introduced. During the Edo era (from 1573 to 1867), legal institutions were developed, consolidated, and unified by the Shogunate-domain law, which replaced the fragmented customary law that was previously in practice. As a result of opening society and the economy to western cultures, in 1868, the Meiji Restoration marked the beginning of Japan's modern legal history. The new legal reform embraced European legal tradition by incorporating French and German codification into its legal framework. French and German jurists

drafted the first civil and commercial codes in 1889 and 1890, respectively [...]. Since then, the two codes have been modified, but their form and structure have remained generally intact [...]. The Japanese Code of Civil Procedure of 1890 was modeled after the German procedural code of 1877, and it was steadily amended up to 1992. The code was then entirely revised" [Acartürk et al. 2005, p. 398]. Thus, *Statute-I* = 0; *Comprehensive-Appeal-I* = 1; *Judgment-Law-I* = 0; *Inquisitorial-I* = 1; *Written-Evidence-I* = 1. "Japanese law does not recognize the principle of stare decisis. Every court is theoretically free not to follow the judicial decisions even of court superior to it. But in practice, precedents have a very strong authority, especially as concerns the decisions of the Supreme Court. The judge who does not follow them must expect that his judgment will be reversed. The judge is not legally bound by a judgment of a superior court except within the limits of the case with which he is concerned (Court Law art. 4). This case law is not a formal source of law, but in reality it plays an extremely important role as a material source. By means of interpretation which in fact is nothing but a veiled form of legislation, judges make main rules. So it could be said that in Japanese law judge-made law is a very important source" [David et al. 1995, p. J-11]. Therefore, *Statute* = 0. The only Japanese colony in the sample—i.e., the Republic of Korea—became independent in 1945, which is after the transplantation of the European codes but before the drafting and promulgation of the new ones.

2.2.35 Jordan

Its "legal history originates back to the 19th century when Jordan was part of the Ottoman Empire. Until World War I, the Ottoman rule imposed on these territories its codification of the civil law aspects of Shari'a and Majallah rules [which] was drafted on the basis of European traditions. Commercial law, introduced during the rule, was a mixture of French, Turkish, and Swiss law. After the collapse of the Ottoman Empire, the League of Nations established British Mandate rule, which inevitably brought the influence of the common law system over commercial and company law in Jordan. After independence [1946], the Jordanian legal system was significantly changed. In 1976, Jordan replaced the Ottoman civil code of 1876 with a new codification based on Syrian and Egyptian civil codes, and since then it has remained intact [...]. The current Commercial Code was enacted in 1966.

The Code transformed the British model of commercial law. French civil procedure rules were first promulgated in Jordan by the Ottoman rule and later replaced by a local code in 1952. The present Code of Civil Procedure in turn, repealed this code in 1988. Jordan's civil procedure rules continue to follow the French legal model" [Acartürk et al. 2005, p. 406]. Thus, *Statute-I* = 0; *Comprehensive-Appeal-I* = 1; *Judgment-Law-I* = 1; *Inquisitorial-I* = 1; *Written-Evidence-I* = 1 (see the report on Turkey). "Main sources of law are its Constitution, legislation, and judicial decisions [...]. Judgments of the Supreme Court are published in the monthly Journals of the Bar Association. They constitute persuasive authority on the interpretation of law" [David et al. 1995, p. J-28]. Since the binding effect of these decisions is significantly less powerful than that of the rulings of the Cassation courts of Argentina, Dominican Republic, Guatemala, Indonesia, Tunisia, and Turkey, *Statute* = 1.

2.2.36 Kenya

Kenya became independent of the UK "in 1963 [...]. [Its private law] has directly followed the common law of England or has been heavily affected by it [...]. Kenya became a protectorate of the United Kingdom in 1895, and a colony in 1920. The first statutory enactment to incorporate English law into Kenyan law was Section 4 of the Kenya Order in Council of 1921, which established that the substance of the common law, the doctrines of equity, and the English statutes of general application passed before August 12th of 1897 were internal law unless repealed by a Kenyan Statute or a later English Statute applicable to Kenya. Indian law has also influenced Kenyan civil procedure [...] During the twentieth century the Kenyan civil procedure has basically followed the nineteenth century procedure in England. The Order of Council of 1921 incorporated as Kenyan law the procedure and practice of the courts of justice entered in force in England on August 12th, 1897" [Acartürk et al. 2005, p. 424]. Thus, *Statute-I* = 0; *Comprehensive-Appeal-I* = 0; *Judgment-Law-I* = 0; *Inquisitorial-I* = 0; *Written-Evidence-I* = 1; *Statute* = 0 [see David et al. 1995, p. K-3].

2.2.37 Korea Republic

"It acquired its independence from Japan in 1945 [...]. Its modern legal history is considered to have commenced in the mid-19th century. The legal system imposed by Japanese rule between 1910 and 1945 reflected many European influences, though it is considered

fragmented and incomplete. The Japanese commercial code was based on the 1899 German codification, and it was applied in Korea until 1945. During the U.S. military occupation from 1945 until 1948, the Korean legal system was also strongly affected by the American legal system. Regulations, such as securities and antitrust law, still reflect the influence of the American law. In the 1960s, Korea tried to eliminate the Japanese legislation and redirected its legal development to follow European models [. . .]. The current Civil Code was enacted in 1960 followed by the Commercial Code of 1962. The 1962 Code was further amended in 1995. [. . .] Korea also adopted a Code of Civil Procedure that came into force in 1960" [Acartürk et al. 2005, p. 432]. Thus, *Statute-I* = 0; *Comprehensive-Appeal-I* = 1; *Judgment-Law-I* = 0; *Inquisitorial-I* = 1; *Written-Evidence-I* = 1. "Korea belongs to the group of countries adhering to the Roman-Civil Law system in which judicial decisions are not generally regarded as binding authority" [David et al. 1995, p. R-6]. Thus, *Statute* = 1.

2.2.38 Kuwait

Kuwait acquired its independence from the UK "in 1961 [. . .]. As a part of the Ottoman Empire until the late 19th century, Kuwait received strong influence of the French civil law [. . .]. During the British protectorate rule between 1899 and 1961, Kuwaiti legislation was not affected by the principles of the common law. Moreover, the Ottoman Majallah, which is considered as the imperial Ottoman version of a civil code, was officially adopted in 1938. After independence, Kuwait initiated revision of the existing legal framework towards codification. The new Civil Code of 1980 that replaced the Ottoman Majallah was developed on the bases of the Egyptian code of 1948 and the Iraqi code of 1951, both of which are derived from French civil law. In the area of commercial law, there is no commercial code, although the 1980 Law of Commerce is regarded as such, and it also follows French and Swiss legal models [. . .]. Kuwait's civil procedural rules are incorporated in the Code of Civil and Commercial Procedure. The Code was adopted in 1980 and was last amended in 2002. It was drafted in accordance with [French law]" [Acartürk et al. 2005, p. 439]. So, *Statute-I* = 0; *Comprehensive-Appeal-I* = 1; *Judgment-Law-I* = 1; *Inquisitorial-I* = 1; *Written-Evidence-I* = 1 (see the report on Turkey). Since judicial decisions are not mentioned among the sources of law in the David et al.'s (1995) report, *Statute* = 1 [David et al. 1995, p. K-20 and K-21].

2.2.39 Lebanon

Lebanon "acquired its independence from the League of Nations mandate in 1943 [...]. Lebanon's modern legal system is based on French [law] combined with Islamic and Ottoman legal principles. Initially, it adopted elements of the Islamic and canon law during the Ottoman rule. At that time, the Ottoman law had already received French, Italian, and Austrian influences and thus, the Lebanese civil and the commercial codes consequently inherited them. As opposed to [French law] where a single civil code is typical, the Majallah [...] served as a civil code in Lebanon. Under the French mandate imposed by the League of Nations from 1920 to 1943, French civil law was introduced through the adoption of a series of laws that replaced parts of the Majallah regulating contracts and property matters. [Indeed] French lawyers drafted the Code of Obligations and Contracts of 1932 and the Code of Commerce of 1942 [...]. After its independence, Lebanon remained very close to [French law]. Since 1993, Lebanon has revised its mercantile legislation modifying the laws on contracts, copyright, obligations, and real property. The recent changes have confirmed that the French legal model continues to influence Lebanese [law]. The civil procedure rules were first established as a separate codification during the French mandate. The Code of Procedure of 1933 was developed on the basis of the French procedure code. The code was entirely replaced by a new Code [in 1983]" [Acartürk et al. 2005, p. 458]. So, *Statute-I* = 0; *Comprehensive-Appeal-I* = 1; *Judgment-Law-I* = 1; *Inquisitorial-I* = 1; *Written-Evidence-I* = 1 (see the report on Turkey). "The application and interpretation of legislation has given rise to a mass of case law. This [...] does not constitute a source of law in the technical sense of the term. Decided cases give rise to no ratio decidendi having validity in other cases; nor do they have the force of precedent" [David et al. 1995, p. L-11]. So, *Statute* = 1.

2.2.40 Lesotho

"The territory which was formerly the British Colony of Batuseland became on 4 Oct. 1966 the sovereign democratic Kingdom of Lesotho [...]. The Common Law, received on 29 May 1884, is "the law for the time being in force in the Colony of the Cape of Good Hope" [David et al. 1995, p. L-23]. "The courts are often guided by South African and [...] English decisions" [David et al. 1995, p. L-23]. Thus, *Statute-I* = *Statute* = 0.

2.2.41 Liberia

"The Constitution of Liberia is patterned after that of the United States [...]. There is no general codification in Liberia; the statutory system is qualitatively similar to that of the United States [...]. In addition to statutory law, Anglo American Common Law may be modified by the Liberian Common Law which is found in the decision of the Liberian courts" [David et al. 1995, p. L-27 and L-28]. Hence, *Statute-I* = *Statute* = 0.

2.2.42 Malawi

"Malawi became a Republic on 6 July 1966, exactly two years after the grant of the independence [by the United Kingdom] [...]. The general legal system of the Protectorate was founded upon the British Central Africa Order in Council, 1902, which provided for the reception of English law, and the survival of African customary law subject to certain limitations. The received English law was expressed in the Order in Council to be "the substance of the Common Law, doctrines of equity, and statutes of general application in force in England on the eleventh day of August, 1902 [and] other applied Acts of the [UK] Parliament and Orders in Council which were in force immediately before [independence]" [David et al. 1995, p. M-11 to M-13]. Hence, *Statute-I* = 0; *Comprehensive-Appeal-I* = 0; *Judgment-Law-I* = 0; *Inquisitorial-I* = 0; *Written-Evidence-I* = 1. "The Republic of Malawi (constitution) Act provides for the continued application of "the substance of the common law and doctrines of equity" [David et al. 1995, p. M-13]. Thus, *Statute* = 0.

2.2.43 Malaysia

Malaysia "gained its independence from the United Kingdom in 1957 [...]. The strong impact of English common law on the Malaysian legal system dates back as far as the end of the 18th century. During this colonial period, British legislation was in force and remained valid until Japanese occupation in 1942. The Federation of Malaysia was established in 1957, and, at present, it consists of 13 states. Each state reserved its own constitution and legislative powers. Islamic and customary laws have had great influence over the Malaysian law with variations across states [...]. Malaysia [...] still follows the English common law [which] covers areas such as contracts, sales, company, securities and tax law. Civil and commercial laws are regulated by judicial interpretations or a number of separate acts. The

Civil Law Act of 1956 provides that in the absence of a specific statute, common law is to be followed. The last amendment to the civil act was in 1984. The British also introduced commercial law during the colonial period. Malaysia's civil procedure and practice of the courts remain close to the English civil procedure and practice" [Acartürk et al. 2005, p. 484]. Thus, *Statute-I* = 0; *Comprehensive-Appeal-I* = 0; *Judgment-Law-I* = 0; *Inquisitorial-I* = 0; *Written-Evidence-I* = 1; *Statute* = 0 [see David et al. 1995, p. M-22].

2.2.44 Montserrat

It get independent from the UK in 1962 (Britannica, 2015). "[B]asic law is the English law [of] 1642" [David et al. 1995, p. U-117]. So, *Statute-I* = *Statute* = 0.

2.2.45 Myanmar

"When Burma was annexed by the British and became a province of India, the law was administered there on principles similar to those adopted in India. The statutory laws which were designed on the English Common Law models for use in India, the portion of law introduced into India without legislation and the rules of law resting upon the authority of the courts were made also applicable to Burma, with the exception of the areas inhabited by national minorities [...]. The British courts gave Hindus and Muslim the benefit of the personal law in religious and family matters and applied the Burmese Law to Buddhists in the same field. Under the Burma Laws Act, 1895 [...] the scope of the Burmese Buddhist Law was confined to questions regarding succession, inheritance, marriage, caste, religious, usage and institution, where the parties were Buddhists [...]. Many of the conflicts, traceable to [...] the Burma Laws Act [...] have been resolved by judicial decision and legislation [...]. The legal system of the British colonial period has been retained, to a considerable extent, in independent Burma [1948]" [David et al. 1995, p. B-81]. Thus, *Statute-I* = *Statute* = 0.

2.2.46 Namibia

It "acquired its independence from the South African mandate in 1990 [...]. Namibia's legal system is premised on constitutional supremacy with a mixture of Roman-Dutch and English common law and also incorporates features of customary law. Roman-Dutch law was introduced in 1920 from the Colony of the Cape of Good Hope. The legal system that developed was mainly based on the principles of the Roman-Dutch law rather than the English

common law. However, English common law affected the Roman-Dutch law as applied in the Union of South Africa. Rules of evidence, procedural law in both civil and criminal matters, and the organization of the courts closely followed the English model. In addition administrative and commercial law and practice also evolved to bear the imprint of the English common law system. As in the English common law system precedent of a higher court is binding on lower courts in the Roman-Dutch legal system. Since independence, Namibia has continued to apply the Roman-Dutch laws in force immediately before independence. The Constitution of 1990 specifies that all laws in force immediately before independence remain in force until repealed or amended, or declared unconstitutional. Namibia's civil procedure and practice of the courts also follow very closely the English civil procedure and practice via the South African influence [...]. The rules of procedure and evidence, the methods of proof and the treatment of precedents are clearly within the English common law tradition. The constitution retained all in-force procedural laws, and courts still use pre-independence South African court decisions as precedent for interpreting current South African laws" [Acartürk et al. 2005, p. 536]. Thus, *Statute-I* = *Statute* = 0; *Comprehensive-Appeal-I* = 0; *Judgment-Law-I* = 0; *Inquisitorial-I* = 0; *Written-Evidence-I* = 1.

2.2.47 Nauru

Nauru was under the joint control of Australia, UK, and New Zealand from 1914 to 1966 (Britannica, 2015). "The Constitution is "the supreme law of Nauru" [...]. It also provides for the continuance, subject to the Constitution itself, of the law in force immediately before independence [and] [...] the principles and rules of the Common law and Equity in force in England" [David et al. 1995, p. N-2]. Thus, *Statute-I* = *Statute* = 0.

2.2.48 Nepal

"In 1854, Jung Bahadur, the first Rana prime minister, promulgated a code called the muluki ain. This code represented a considerable reform of the existing law and showed traces of British influence [...]. Both the present muluki ain and the statutes in the ain sanghra are considerably influenced by English and Indian laws" [David et al. 1995, p. N-6]. As a consequence, *Statute-I* = 0. "The source of ordinary law are legislation, judicial precedents and custom" [David et al. 1995, p. N-6]. As a result, *Statute* = 0.

2.2.49 New Zealand

New Zealand is "a fully independent member of the British Commonwealth [...]. There was no legal system in existence when the British first settled the islands in the early 19th century, and the whole body of English common law and principles of equity was introduced in 1840. The current sources of New Zealand law consist of about 70 basic U.K statutes as well as a large body of local statutes, many of which were modeled on earlier English legislation. New Zealand, like the United Kingdom, does not have a written constitution, but rather a series of acts composed by the U.K and New Zealand parliaments, including the Constitution Act of 1986 and the New Zealand Bill of Rights Act 1990. Private law has been substantially developed by case law, and unified through appeals to the Judicial Committee of the Privy Council in London. New Zealand closely follows the English civil procedure and practice" [Acartürk et al. 2005, p. 564]. Thus, *Statute-I* = 0; *Comprehensive-Appeal-I* = 0; *Judgment-Law-I* = 0; *Inquisitorial-I* = 0; *Written-Evidence-I* = 0. "Common law and of Equity remain distinct as they do in England, but where there is conflict between the two the rules of Equity are to prevail. The relevant principles have been developed by the New Zealand Courts (including the Privy Council) by precedent. The Magistrates' Courts are bound by the decisions of all superior courts, the Supreme Court by the decisions of the Court of Appeal and the Privy Council, the court of Appeal by the decisions of the Privy Council and, to some extent, by its own decisions. The New Zealand courts adhere to view that it is desirable that the Common Law should so far as possible be kept uniform throughout the Commonwealth" [David et al. 1995, p. N-32]. So, *Statute* = 0.

2.2.50 Nigeria

Nigeria "gained independence from the United Kingdom in 1960 [...]. Nigerian legal system is based on the English common law that was imposed during English colonial rule from 1861 to 1960 [...]. After 1966, the Nigerian legal system became more complex with military interference in the government. The Military Decree superceded both the unsuspended portion of the Constitution of the Nation and every preceding Decree inconsistent with its provisions. The Federal laws codified the Common Laws, in a set of books known as the Laws of Federation 1958. In 1988, the Armed Forces Ruling Council announced its program

to reform the legal system by replacing inherited English statutes or clarifying their position within the system. Thus, the Nigerian legal system has undergone a transition from military to civilian rule. The resultant consequence is mere codification of the Laws of the Federation of 1958 and subsisting Decrees" [Acartürk et al. 2005, p. 572]. Therefore, *Statute-I* = 0; *Comprehensive-Appeal-I* = 0; *Judgment-Law-I* = 0; *Inquisitorial-I* = 0; *Written-Evidence-I* = 1. "Inevitably a body of case law has grown around the three types of law administered by the courts, i.e., the received English law, local legislation and customary law. Nigerian decisions as well as relevant English ones are cited by the courts. The English doctrine of judicial precedent applies in Nigeria. Accordingly, the decision of a superior court is binding on an inferior court" [David et al. 1995, p. N-61]. So, *Statute* = 0.

2.2.51 Norway

"During the 400 years Norway was united to Denmark (1380-1814) Norwegian law was gradually influenced by Danish [law]. The judges were to large extent Danes [...]. The codification of Norwegian law in King Christian V's Norwegian code of 1687 is also of major importance in this respect. [...] The works of the great Danish Lawyer Orsted, which to a large extent were published after Norway had become united with Sweden—a union which lasted from 1814 to 1905—are also considered the foundation of modern Norwegian [law]" [David et al. 1995, p. N-77 and N-78]. Thus, *Statute-I* = 0; *Comprehensive-Appeal-I* = 1; *Judgment-Law-I* = 0; *Inquisitorial-I* = 0.5; *Written-Evidence-I* = 0. "Norwegian law is not based upon general codifications [...] [and] there are vast fields of law which might be classified as customary law or judge-made law [...]. The decisions of the courts, especially the Supreme Court have a strong persuasive force [...]. Statute law is considered to be equivalent to [...] judge-made law" [David et al. 1995, p. N-76]. As a result, *Statute* = 0

2.2.52 Pakistan

Pakistan "gained independence from Great Britain in 1947 [...]. The courts of British India applied a combination of native customary and English common law until the latter 19th century, when British India began adopting statutes that codified the main areas of English substantive and procedural law [...]. It was at this stage that colonial law became in principle independent of English law, although in practice, the interpretations of English

courts remained very persuasive. Pakistan's contemporary legal framework does not depart significantly from the colonial framework. [...] [T]he major statutes of the British era were adopted by the legislature. In interpreting statutory provisions, Pakistani courts adhere to the doctrine of stare decisis, and, moreover, accord persuasive value to Indian and English case law" [Acartürk et al. 2005, p. 589]. Thus, *Statute-I* = 0; *Comprehensive-Appeal-I* = 0; *Judgment-Law-I* = 0; *Inquisitorial-I* = 0; *Written-Evidence-I* = 1. "The doctrine of stare decisis is followed in Pakistan. Decisions of the Supreme Court, in so far as they decide question of law, are binding on all other courts in the particular province [...]. Privy Council appeals are no longer available, but the judgments of the Privy Council are still of the highest persuasive value" [David et al. 1995, p. P-6]. Hence, *Statute* = 0.

2.2.53 Papua New Guinea

Papua New Guinea was under the control of Australia between 1904 and 1975 (Britannica, 2015). "According to the [1975] Constitution s. 9 the laws of Papua New Guinea consist of (1) the Constitution, (2) organic laws made under special constitutional authority (s.12) [...], (7) the underlying law. [...] Underlying law is intended to play a role similar to that of the common law in common law countries, viz. to apply in the absence of statute law. It has three components (a) Custom [...]. (b) The principles and rules of common law and equity of England as they existed prior to 16 Sept. 1975, provided that they are not inconsistent with statute law or custom [...]. (c) The law developed by the courts [...]. To assists with this, the precedent system has been codified and includes such modern devices as provisions for prospective overruling, for the resolution of precedential conflicts of authority and for the reconsideration of outdated precedents (Sch. 2.8-2.12). The courts have been given a mandate to engage in purposive interpretation of statutes (s. 109 (4))" [David et al. 1995, p. P-34 and P-35]. As a consequence, *Statute-I* = *Statute* = 0.

2.2.54 Quatar

Quatar acquired independence from the UK in 1971 (Britannica, 2015). "Before this proclamation [...], Qatar was [...] a British protected state" [David et al. 1995, p. Q-1]. With the intention of reforming the legal system toward the civil law tradition, " [s]ince 1961, the Amir has issued a substantive number of Western-inspired laws regulating such matters

as administration, finance, labour, trade, and commercial business" [David et al. 1995, p. Q-1 to Q-4]. Accordingly, case law is not mentioned among the sources of law listed in the David et al.'s (1995) report [see David et al. 1995, p. Q-4]. So, *Statute-I* = 0; *Statute* = 1.

2.2.55 Romania

Romania "acquired its independence from the Ottoman Empire in 1859 [. . .]. Separate comprehensive codifications of civil and criminal law based on Byzantine models appeared in Moldavia and Wallachia in mid-17th century and together with customary law were applied in the ensuing two centuries. [After independence], its legal system was modernized and a new civil and commercial legislation was adopted on the basis of the early 19th century French legislation. A civil code (Codul civil roman) was instituted in 1865 after the French Civil Code of 1804. The commercial code of 1839 [was a translation] of the Napoleonic codes. After the expansion of the Romanian territory in the aftermath of World War I, Austrian civil code [. . .] and parts of the Russian legislation from Bessarabia influenced Romanian civil [. . .] law. In the late 1930s, Romanian legislation was unified and combined with French, Austrian and other Western European influences. The French codifications became a dominant source for the treatment of contracts and obligations. During the socialist period between 1945 until 1989, socialist laws intermingled with pre-war legislation. Since the collapse of the socialist regime in 1989, a considerable part of the presocialist legislation has been reinstated in 1991 to include much of the old civil code. [T]he 19th century Code of Civil procedure is still largely valid [. . .]. The civil procedure code also follows [French law]" [Acartürk et al. 2005, p. 660]. As a consequence, *Statute-I* = 0; *Comprehensive-Appeal-I* = 1; *Judgment-Law-I* = 1; *Inquisitorial-I* = 1; *Written-Evidence-I* = 1 (see the report on Turkey). "Case law is not a source of law. [The] decisions of the Supreme Court are of particular importance. These decisions [. . .] have an interpretative function and ensure the uniform judicial application of the laws" [David et al. 1995, p. R-29]. Thus, *Statute* = 1 .

2.2.56 Sierra Leone

Sierra Leone was a British Crown colony between 1896 and 1961 (Britannica, 2015). The sources of law are "1. The Constitution of 14 June 1978. 2. Legislation consists of Acts adopted since independence and of adopted English statutes [. . .]. 3. Customary law [. . .].

4. Islamic law [...]. 5. English common law and equity, together with customary law, form part of the common law" [David et al. 1995, p. S-48]. So, *Statute-I* = *Statute* = 0.

2.2.57 Singapore

"Singapore became a separate kingdom of the United Kingdom in 1946 and a self-governing state in 1959. It joined Malaysia in 1963 for two years, though it became an independent nation of the Commonwealth in 1965. As a result, Singapore's legal system is largely based on the English common law with customary influences, though the legal system also remains closely tied to the development of the Malaysian legal system. The Application of English Law act (Chapter 7A, 1994 Edition) provides for the continuation in force in Singapore of common law (including equity) so far as it is applicable to the circumstances of Singapore and its inhabitants. Singapore's commercial law is developed on the basis of the English common law model, while customary, and Islamic law governs personal relations. Singapore's civil procedure and practice of the courts also follow the English legal model. English civil procedure tradition is adopted through Rules of Court. The rules came into force in 1996" [Acartürk et al. 2005, p. 689]. Thus, *Statute-I* = 0; *Comprehensive-Appeal-I* = 0; *Judgment-Law-I* = 0; *Inquisitorial-I* = 0; *Written-Evidence-I* = 1. "The English doctrine of precedent applies in Singapore" [David et al. 1995, p. S-57]. Thus, *Statute* = 0.

2.2.58 Solomon Island

"The general law is provided for in Const. s. 76 and Sched. 3. It consists of (1) the Acts of Parliament of the United Kingdom of general application and in force on 1 Jan. 1961, with necessary adaptations; (2) the principles and rules of the common law and equity except insofar as they are inconsistent with the Constitution or an Act of the Parliament [...]; and (3) customary law" [David et al. 1995, p. S-65]. Thus, *Statute-I* = *Statute* = 0.

2.2.59 South Africa

South Africa "acquired its independence from England in 1910 [...]. South Africa's legislation is based on Roman-Dutch law and English common law. The Roman-Dutch law was roughly inherited from the Netherlands during the 18th and 19th centuries. The Cape of Good Hope was under British rule in 1795 and 1806. Common law replaced the Roman-Dutch legislation after the British occupation in 1806. Under British rule English

law made its influence via legislation including the administration of justice (the judicial precedents in particular). Some statutes expressly stipulate that English law was applicable to certain matters like the commercial laws (including negotiable instruments and company law) and the civil and evidence procedure. The South African legal system generally follows the common law, which is judge-made law. South Africa's civil procedure and practice of the courts follow the English civil procedure and practice. Common law applies, unless overridden by statute" [Acartürk et al. 2005, p. 711]. Thus, *Statute-I* = 0; *Comprehensive-Appeal-I* = 0; *Judgment-Law-I* = 0; *Inquisitorial-I* = 0; *Written-Evidence-I* = 1. "The two main sources of modern South African law are legislation and case law [...]. In accordance with stare decisis, judicial precedents is a source of law. Every court is bound by the decisions of a court superior to it. The Appellate Division is bound by its own decisions, but may depart from an earlier decision if it is satisfied that it was wrong. The decisions of Magistrates' Courts do not create precedents" [David et al. 1995, p. S-79]. So, *Statute* = 0.

2.2.60 Sri Lanka

Sri Lanka "gained independence from the British in 1948 and is now an independent member of the British Commonwealth [...]. The Portuguese, who conquered and ruled the maritime provinces of the island in 1505, surrendered to the Dutch in 1656, who in turn ceded control of the maritime provinces to the British in 1796. Upon the Kandyan Kingdom's accession to the British Crown by the Treaty of 1815, the entire island fell under British rule and continued as a British colony until 1948. The legal system of the country has been influenced by the colonial rulers that governed the island during these periods. The Dutch established an elaborate system of courts in the areas they ruled and administered the law as set out in the Statutes of Batavia. During the British era, Roman-Dutch Law continued to be the law administered by the courts. However, the English, in administering the law, departed from and modified the Roman-Dutch principles by applying English legal principles or by giving effect to local customs and practices. The application of Roman-Dutch Law was further restricted by the enactment of statutes based on English Law. Today, Roman-Dutch law remains the residuary law (i.e., the courts may have recourse to it where it has not been superceded by statute or judicial precedent). Sri Lanka's constitutional and

administrative law is predominantly Anglo-American in origin and its commercial/corporate law is almost wholly based on the principles of English commercial law. Procedural [...] law is also derived from English law" [Acartürk et al. 2005, p. 730]. Thus, *Statute-I* = 0; *Comprehensive-Appeal-I* = 0; *Judgment-Law-I* = 0; *Inquisitorial-I* = 0; *Written-Evidence-I* = 1. "[S]tare decisis applies in Sri Lanka. A decision of the Supreme Court is binding on all other courts. A decision of the Court of Appeal is binding on the courts of first instance, if it is not in conflict with a decision of the Supreme Court. Since the Constitution of 1978 entered into force earlier decisions of the English Privy Council and of the former Supreme Court (before 1978) are no longer binding" [David et al. 1995, p. S-129]. Thus, *Statute* = 0.

2.2.61 St. Kitts and Nevis

The island of Nevis was colonized in 1623 by British settlers from Saint Kitts and Saint Kitts and Nevis became an associated state in 1967 and an independent one in 1983 (Britannica, 2015). "The basic law probably consists of the law in force in 1623 [...]. The Common Law (Declaration of Application) Act provides that the common law of England so far as it stands unaltered by any written laws of the Islands is in force there. Local legislation follows largely analogous English laws in the fields of private and commercial law, [...] and procedure" [David et al. 1995, p. S-3]. As a result, *Statute-I* = *Statute* = 0.

2.2.62 St. Vincent

"During the 16th century the French established control over the island. France ceded St. Vincent to the British Empire by the Treaty of Paris in 1763, and re-claimed it back in 1779. St. Vincent was again returned to Britain in 1783 under the Treaty of Versailles, and remained under British control over the next 200 years. From 1958 until 1962, St. Vincent was part of the West Indies Federation and in 1969 it adopted an associated state status with full internal self-government. In 1979, St. Vincent and the Grenadines became an independent country within the British Commonwealth. St. Vincent's legal system is based on English common law and has remained very closely linked to the laws of the United Kingdom since colonial times [...]. The current Vincentian constitution was adopted in 1979. The civil procedure and practice of St. Vincent's courts also follow very closely the English civil procedure and practice [...]. The judicial organization of St. Vincent is based on the

English common law system" [Acartürk et al. 2005, p. 741]. As a consequence, *Statute-I* = 0; *Comprehensive-Appeal-I* = 0; *Judgment-Law-I* = 0; *Inquisitorial-I* = 0; *Written-Evidence-I* = 1. *Statute* = 0 [see also David et al. 1995, p. U-117].

2.2.63 Sudan

Sudan was under the English control between 1882 and 1956 (Britannica, 2015). "The transitional Constitution of the Sudan was adopted by the Transitional Military Council on 10 Oct. 1985. There has been an attempt at codification of large areas of the law [...]. Although the bulk of these new legislations introduced some terms and concepts derived from Islamic jurisprudence, yet the majority of the provisions of these legislations are a re-enactment of the Sudanese legislation previously in force. As a result of recent codification, the scope for judicial legislation has been greatly narrowed. But the courts are still allowed in the area of civil law to resort to sources other than legislation. The Civ. Proc. A s. 6 (2) provides: "In case not provided for by any law, the courts shall act according to Sharia law, Sudanese judicial precedents, customs, justice and good conscience." Despite the striking resemblances of the terms of this provision to those of previous law under which the courts have previously applied the English common law, it seems that this possibility is too remote under new circumstances" [David et al. 1995, p. S-141]. So, *Statute-I* = 0 and *Statute* = 1.

2.2.64 Taiwan

"As a part of China until 1895, Taiwan's legal history [...] followed Chinese legal traditions. During the first decades of the 20th century, Taiwan was ceded to Japan. It reverted back to Chinese control in 1945 when the Nationalist Chinese administration established a capital in Taipei, where they ruled under the Chinese Constitution of 1946. Modern Chinese law and consequently the Taiwanese legal system are strongly influenced by the European civil law through Swiss and German codifications. As a part of the reform, the Chinese civil code of 1929 was slightly amended in 1982. Political reform along with legal reform took place from the late 1980s to the early 1990s. The process of modification of the civil law brought that particular part of Taiwanese legal system close to American models, while the rest of the codified legislation remains closer to the European sources [...]. The Code of Civil Procedure follows the European legal tradition since Taiwan adopted the Chinese

procedural law and revised it in 1968" [Acartürk et al. 2005, p. 773]. Thus, *Statute-I* = 0; *Comprehensive-Appeal-I* = 1; *Judgment-Law-I* = 0; *Inquisitorial-I* = 1; *Written-Evidence-I* = 1. "[C]ourts are not bound by precedent. However, resolutions of the Supreme Court or the Supreme Administrative Court may become binding precedents when they are final judgments and are selected as precedents based on decisions of those two courts" [Acartürk et al. 2005, p. 773]. Since appeals to the Supreme Court are made very difficult by the relevant statutes [see David et al. 1995, p. R-1 and R-2], the binding effect of these decisions is significantly less powerful than that of the rulings of the Cassation courts of Argentina, Dominican Republic, Guatemala, Indonesia, Tunisia, and Turkey. Thus, *Statute* = 1.

2.2.65 Tanzania

"After gaining independence from the United Kingdom in 1961 and 1963, respectively, the former colonies of Tanganyika and Zanzibar merged in 1964 to form the United Republic of Tanzania [...]. [M]ost of its private and public law has directly followed the common law of England or has been heavily influenced by it. Initially, German imperial law was introduced upon colonization and it co-existed with local Customary and Sharia law. The first statutory enactment to incorporate English law in Tanganyika established that Common Law, the Doctrines of Equity and the English Statutes of General Application in force in England on July 22nd, 1920 were internal law unless repealed by a local statute or a later English statute applicable to Tanganyika. The Tanzanian civil procedure and practice of the courts also follow very closely the English Civil procedure and practice. Common law also applies, unless overridden by statute [...]. A Civil Procedure Code was enacted in 1966, which is largely in pari-materia with the Indian Civil Procedure Code, 1908, the latter being a codification of English Common Law Civil Procedure development as part of the Common Law and Rules of Equity" [Acartürk et al. 2005, p. 780]. Thus, *Statute-I* = 0; *Comprehensive-Appeal-I* = 0; *Judgment-Law-I* = 0; *Inquisitorial-I* = 0; *Written-Evidence-I* = 1. "The jurisdiction of the court [is] exercised in conformity with the substance of the Common Law, the doctrines of Equity and the statutes of general application in force in England on 22 July 1920" [David et al. 1995, p. T-4]. Thus, *Statute* = 0.

2.2.66 Thailand

"The Thai legal system developed in the 14th century and was based on the ancient Hindu Code of Manu and traditional Thai customs. The main source of law at that time was the Dhammasattham and remained the cornerstone of Thai law until the late 18th century. At the beginning of the subsequent rule, Thailand gave way to some European legal influences" [Acartürk et al. 2005, p. 789]. "The Civil Procedure Code, promulgated in B.E. 2478 (1935) [...], applies equally to both civil and commercial cases. [It is] similar to those of the Chinese, German and Japanese Civil Procedure Codes" [David et al. 1995, p. T-15]. So, *Statute-I* = 0; *Comprehensive-Appeal-I* = 1; *Judgment-Law-I* = 0; *Inquisitorial-I* = 1; *Written-Evidence-I* = 1. Judicial decisions are not mentioned among the sources of law in the David et al.'s (1995) report [David et al. 1995, p. T-11 and T-12]. So, *Statute* = 1.

2.2.67 Tonga

Tonga was part of the British Western Pacific Territories from 1901 until 1952 (Britannica, 2015). "Tonga customary law has been codified and none remains unwritten. The Common Law of England and the rules of Equity together with the statutes of general application in force in England of 18 Oct. 1966, are applied (1) only so far as no other provision has been or may therefore be made by or under any Act or Ordinance in force in the Kingdom of Tonga; and (2) only so far as the circumstances of the Kingdom of Tonga or of its inhabitants permit and subject to such qualification as local circumstances render necessary (Civil Law Act, cap. 14)" [David et al. 1995, p. T-26]. Thus, *Statute-I* = 0; *Statute* = 1.

2.2.68 Trinidad and Tobago

It "acquired its independence from the United Kingdom in 1962, and is at present a member of the British Commonwealth [...]. Trinidad's legal system was based on Spanish civil law while it was under Spanish colonial rule and Spanish law continued to apply after it was ceded to Britain in 1802. Gradually English common law was introduced and replaced the Spanish legislation. In 1848, all existing Spanish laws were officially abrogated and the English common law, statutes and principles of equity, of that time were adopted in Trinidad and Tobago. After having been under Dutch, French and English rule, common law was adopted in Tobago following formal succession to Britain in 1814. Trinidad and Tobago's

legal system is based on English common law and has remained very closely linked to the laws of the United Kingdom and other British Commonwealth countries since colonial times. The current constitution was adopted in 1976. Trinidad and Tobago still closely follows the English legal model both in drafting of new legislation and in its interpretation though there is substantive input from other British Commonwealth countries. Private law has been substantially developed by case law, and unified through appeals to the Judicial Committee of the Privy Council in England. Trinidad and Tobago's civil procedure and practice of the courts also follow very closely the English civil procedure and practice" [Acartürk et al. 2005, p. 798]. Thus, *Statute-I* = 0; *Comprehensive-Appeal-I* = 0; *Judgment-Law-I* = 0; *Inquisitorial-I* = 0; *Written-Evidence-I* = 1. "[D]ecisions of the House of Lords and the Judicial Committee in England are binding on, and of its Court of Appeal are normally followed by, the courts" [David et al. 1995, p. T-29 and T-30]. Thus, *Statute* = 0.

2.2.69 Turkey

"The Republic of Turkey was established in October 29, 1923 after the collapse of the Ottoman empire" [Acartürk et al. 2005, p. 814]. "When the decline of the Ottoman empire became all too evident, at the beginning of the nineteen century, Turkey turned to the West. It was hoped that the adoption of Western legal institutions might bring in its train an upsurge in the life of the community [...]. This led to the reception of foreign codes: [...] the Commercial Code (1850); the Code of Maritime Commerce (1864); [...] the Code of Civil Procedure (1880). Most of these codes were based upon French models [...]. In consequence of this reception of foreign law, the court system became a dual one: Islamic law was applied by the sharia courts, the law received from abroad, in the ordinary courts. This dualism formed a remarkable parallel to the division of the Common law and Equity courts in England before 1875. [In] 1926, the grand National Assembly enacted the Turkish Civil code, based upon a reception of the Swiss Civil Code. The Turkish translation is based upon the French version of the Code. It came into force [in] 1926. [...] The Code of Civil Procedure is contained in Law no. 1086 of 18 June 1927, a reception of the Code of Civil Procedure of the Swiss Canton Neuchâtel" [David et al. 1995, p. T-48 to T-51] Thus, *Statute-I* = 0; *Comprehensive-Appeal-I* = 1; *Judgment-Law-I* = 1; *Inquisitorial-I* = 1; *Written-Evidence-I*

= 1. David et al. (1995) rank the sources of law as follows: "1. the Constitution; 2. Statute Law; [...] 3. The decisions of the Court of Cassation ensuring uniform judicial practice are also to be regarded as sources of law, since they must be followed by the courts" [David et al. 1995, p. T-47 and T-48], i.e.., "they are binding on all the courts (cf. Law no. 1730 of 16 May 1973 on the Court of Cassation)" [David et al. 1995, p. T-47]. Thus, *Statute* = 0.

2.2.70 Turks and Caicos

It is "an overseas territory of the United Kingdom. The British monarch is the head of state, represented by a governor, and the people elect the Legislative Council [...] British colonial rule was established in 1766. After colonization by the British the Turks and Caicos Islands were administered as part of the Bahamas until 1848. In 1874 they became a Jamaican dependency. After Jamaica's independence in 1962 the Islands became a separate British dependency and were made a separate British Crown Colony in 1973. The Turks and Caicos Island's legal system is based on English common law. There was no legal system in existence when the British first settled the Islands in 1766, and only then was English law introduced. The current law is the English common law, and the statutes and legal principles of 1766, as amended by subsequent local or U.K. legislation. Due to the Islands' relationship with Jamaica, a significant amount of legislation through the post-war period was of Jamaican origin [...]. The current constitution was adopted in 1976, suspended in 1986 and revised and amended in 1988. Private law has been substantially developed by case law, and unified through appeals to the Judicial Committee of the Privy Council in London. The civil procedure and practice of the courts of the Turks and Caicos Islands also follow very closely the English civil procedure and practice" [Acartürk et al. 2005, p. 825]. Thus, *Statute-I* = 0; *Comprehensive-Appeal-I* = 0; *Judgment-Law-I* = 0; *Inquisitorial-I* = 0; *Written-Evidence-I* = 0; *Statute* = 0 [see David et al. 1995, p. U-118 and U-119].

2.2.71 Uganda

Uganda "acquired its independence from the United Kingdom in 1962 and succeeded from the British Protectorate [...]. In the 1860s, British explorers arrived in the country and were soon followed by Protestant and Catholic missionaries. In the 1880s, Uganda's territory came under increasingly British influence and was assigned by royal charter to the British East

Africa Company. After the British East Africa Company ceased to administer the territory in 1893, a British commissioner took over administrative functions, and from 1894 until 1961 Uganda existed as a British Protectorate. In 1961, Britain granted internal self-government and the first local elections were held. Independence followed in 1962. Uganda was declared a republic in 1967. Uganda's legal system is based on English common law, although it also incorporates elements of customary law. The English common law, statutes and principles of equity of 1902 were adopted as the governing law by the 1902 "Order in Council." In 1961, a new Order in Council was passed amending the 1902 Order. The Judicature Act 1996 now provides for the applicability of common law and doctrines of equity, any established and current custom or usage and customary law which is not repugnant to natural justice, equity and good conscience, all subject to the written law. Currently, the applicable laws in Uganda are written law, common law, the doctrines of equity, custom, principles of natural justice, and equity and good conscience. Commercial legislation in Uganda follows the English tradition and has been modernized since independence. The current Constitution was adopted in 1995. The Ugandan civil procedure was influenced by English law as applied in India and follows closely the English civil procedure and practice. Ugandan courts apply common law, statutory law and customary law" [Acartürk et al. 2005, p. 840]. Thus, *Statute-I* = 0; *Comprehensive-Appeal-I* = 0; *Judgment-Law-I* = 0; *Inquisitorial-I* = 0; *Written-Evidence-I* = 1; *Statute* = 0 [see also David et al. 1995, p. U-3 and U-4].

2.2.72 United Arab Emirates

United Arab Emirates's (UAE) "acquired its independence from the United Kingdom in 1971 [...]. The first source in the UAE's legal system was the treaties that the British concluded with the seven emirates of the Trucial Coast in the 19th century. After the withdrawal of British military forces in 1971, the Emirates adopted a provisional constitution that was extended for five-year period increments until 1996, when the permanent document was enacted [...]. The UAE Constitution affirms the Islamic Shari'a as a principal source of legislation and gives each Emirate a considerable degree of local autonomy. Recent legal development is trending towards enacting legislation at the federal level, mainly governing civil and commercial transactions, business enterprises, banking, and foreign trade. As a

result, in 1985, the UAE adopted its Civil Code, which recognizes the Shari'a as a separate legal system governing personal relations. In addition, the UAE developed a new Federal law on commercial transactions that came into force in 1993 and serves as a Commercial Code. The Federal Code of Civil Procedure, which was adopted in 1992, follows the [...] expansion of federal authority" [Acartürk et al. 2005, p. 832]. As a consequence, *Statute-I* = 0; *Comprehensive-Appeal-I* = 0; *Judgment-Law-I* = 0; *Inquisitorial-I* = 0; *Written-Evidence-I* = 1. Case law is not mentioned among the sources of law in the David et al.'s (1995) report [see David et al. 1995, p. U-53 to U-57]. Therefore, *Statute* = 1.

2.2.73 United States of America

The US "acquired its independence from England in 1776 [...]. British common law forms the historical and conceptual foundation of the American legal system. American and English laws, however, have been independent since the declaration of American independence. As the United States expanded westward, nearly every state adopted the common law system with the exception of Louisiana, which has a legal system based on French civil law. American law is both judge-made and statutory" [Acartürk et al. 2005, p. 873]. Thus, *Statute-I* = 0. The Federal Rules of Civil Procedure (FRCP) established in 1938, replaced the earlier Field Code, which resembled the system of civil procedure used in England under the Rules of the Supreme Court [see David et al. 1995, p. U-153 and U-158]. Thus, *Comprehensive-Appeal* = 0; *Judgment-Law-I* = 0; *Inquisitorial-I* = 0; *Written-Evidence-I* = 0. "Although the body of statute law has grown steadily, the role of the courts as law creators has not diminished. The courts exercise this function in three ways: (1) through control of the constitutionality of legislation; (2) through the interpretation of the written law; and (3) through the constant reformulation of the unwritten law" [David et al. 1995, p. U-141]. Thus, *Statute* = 0.

2.2.74 Western Samoa

"New Zealand legislative action to severe the tie with Western Samoa was taken by the Western Samoa Act 1961 (no. 68 of 1961); and the General Assembly agreed by Resolution 1626 [...]. The law in Samoa consists of [...] the New Zealand legislation in force in Western Samoa immediately before 1 Jan. 1962 [which was, as the related report shows, the one prevailing in the UK], legislation of the Parliament of Western Samoa and of its

predecessor, "the English common law and equity for the time being in so far as they are not excluded by any other law in force in Western Samoa," and custom or usage which acquired force of law" [David et al. 1995, p. W-1 and W-2]. Hence, *Statute-I* = *Statute* = 0.

2.2.75 Zambia

"Zambia became an independent republic within the Commonwealth on the date on which it attained independence, 24 Oct. 1964. Prior to this it was administered as a British protectorate. It first came under the British rule in 1899, when it was placed under the authority of the British South Africa Company by the Barotseland-North western Rhodesia Order in Council of 1899, which was supplemented a year later by the North Eastern Rhodesia Order in Council. The two areas were explicitly amalgamated into the Protectorate of Northern Rohodesia in 1922, although the British South Africa Company continued to be the administering authority on behalf of the British government until 1924. [Next] direct responsibility [...] was assumed by the United Kingdom government through the Colonial Office [...]. The English Law [...] Act (Laws of Zambia Cap. II), originally passed on 8 March 1963, emphasizes what is laid down in the High Court Act s. 13, that in civil matters common law and equity for the time being in force shall be applied concurrently in Zambia" [David et al. 1995, p. Z-16 to Z-19]. Thus, *Statute-I* = *Statute* = 0; *Comprehensive-Appeal-I* = 0; *Judgment-Law-I* = 0; *Inquisitorial-I* = 0; *Written-Evidence-I* = 1.

2.2.76 Zimbabwe

Zimbabwe "acquired its independence from the United Kingdom in 1980 [...]. In the 1880s Zimbabwe's territory came under increasingly British influence. Northern and Southern Rhodesia (Zambia and Zimbabwe, respectively) were declared a British sphere of influence [...]. In 1898 Southern Rhodesia was organized as a separate colony, and [in 1923] was formally annexed by the United Kingdom and granted internal self-government. After the federation's dissolution in 1963, Southern Rhodesia unilaterally declared its independence under the name of Rhodesia. However, the unilateral declaration of independence did not immediately receive recognition from the United Kingdom or the international community. Only after a period of economic sanctions imposed by the United Nations Security Council and an armed uprising were free elections held in 1979 and was independence negotiated

with the U.K. In 1980 Rhodesia became independent under the name of Zimbabwe. Zimbabwe's legal system is a combination of Roman-Dutch and English common law and also incorporates features of customary law. Roman-Dutch law was introduced in 1891 from the Colony of the Cape of Good Hope. By importing the law of South Africa, the legal system that developed was primarily based on the principles of the Roman-Dutch law rather than the English common law. However, constitutional, administrative and commercial law and practice were also based on the English models. As in the English common law system precedent of a higher court is binding on lower courts in the Roman-Dutch legal system [...]. The Zimbabwean civil procedure and practice of the courts also follow very closely the English civil procedure and practice. [All in all, the] rules of procedure and evidence, the methods of proof and the treatment of precedents are clearly within the English [law]" [Acartürk et al. 2005, p. 905 and 906]. Thus, *Statute-I* = *Statute* = 0; *Comprehensive-Appeal-I* = 0; *Judgment-Law-I* = 0; *Inquisitorial-I* = 0; *Written-Evidence-I* = 1.

3 References

Acartürk, Burcu, et al. 2005. *Courts: The Lex Mundi Project. Civil Procedures Around the World. Volume 2: Country Appendix of Data for Eviction of Non-Paying Tenant and Collection of a Bounced Check.* Harvard University Press.

Britannica (Encyclopedia). 2015. *Encyclopedia Britannica.* http://www.britannica.com

Campbell, Christian, and Dennis Campbell. 1995. *International Civil Procedures.* Lloyd's of London Press Ltd., Inc., London. H. Nev.

David, René, et al., ed. 1995. *International Encyclopedia of Comparative Law.* Volume 1, *National Reports.* New York: Oceana Publications Incorporation.

Engelmann, Arthur. 1927. *A History of Continental Civil Procedure.* Boston: Little, Brown and Company.

Finlason, William F., 1877. *An Exposition of Our Judicial System and Civil Procedure as Reconstructed Under the Judicature Acts.* London: Longmans, Green, and Company.

Glendon, Mary Ann, Michael W. Gordon, and Paolo G. Carozza. 1999. *Comparative Legal Traditions in a Nutshell.* St. Paul: West Group.

Lee, Robert W. 1915. *An Introduction to Roman-Dutch Law.* Oxford-New York: Oxford University Press.

Stephen, James Fitzjames, 1875. *Supreme Court of Judicature Acts.* Stevens and Sons, London.

Ward, Richard, and Amanda Wragg. 2005. *English Legal System.* 9th ed. Oxford-New York: Oxford University Press.

Zweigert, Konrad, and Hein Kötz. 1998. *Introduction to Comparative Law.* 3rd ed. Oxford-New York: Oxford University Press.
