# Supplementary material for: A novel dataset on legal traditions, their determinants, and their economic role in 155 transplants
Source: Data Brief. 2016 May 31;8:394–8. doi: 10.1016/j.dib.2016.05.049 (PMC4908268; doi:10.1016/j.dib.2016.05.049)
Supplement: Supplementary file 4 — Supplementary material [file mmc4.pdf]

From:
Carmine Guerriero,
Assistant Professor,
ACLE, University of Amsterdam.
Plantage Muidergracht, 12,
1018 TV Amsterdam, the Netherlands.

To:
Hao-Ran Wang,
Novartis Institutes for BioMedical Research,
250 Massachusetts Avenue,
Cambridge, MA 02139, USA.

April 27th, 2016

Dear Professor Wang,

this is to declare that I have no relevant or material financial interests that relate to the research described in the paper I am submitting.

Yours Sincerely,
Carmine Guerriero.
